# Gypenosides exert cardioprotective effects by promoting mitophagy and activating PI3K/Akt/GSK-3*β*/Mcl-1 signaling

**DOI:** 10.7717/peerj.17538

**Published:** 2024-06-20

**Authors:** Yizhe Zheng, Wei Wei, Yukun Wang, Tingting Li, Yundong Wei, Si Gao

**Affiliations:** 1 Department of Pharmacy, School of Medicine, Guangxi University of Science and Technology, Liuzhou, Guangxi, China; 2 Department of Pharmacy, Shaanxi Provincial People’s Hospital, Xi’an, Shaanxi, China; 3 School of Science, Guangxi University of Science and Technology, Liuzhou, Guangxi, China

**Keywords:** Gypenosides, GSK-3β, Heart failure, Mcl-1, Mitochondria, Mitophagy, PI3K/Akt pathway

## Abstract

**Background:**

*Gynostemma pentaphyllum* (Thunb.) Makino, a well-known edible and medicinal plant, has anti-aging properties and is used to treataging-associated conditions such as diabetes, metabolic syndrome, and cardiovascular diseases. Gypenosides (GYPs) are the primary constituents of *G. pentaphyllum*. Increasing evidence indicates that GYPs are effective at preserving mitochondrial homeostasis and preventing heart failure (HF). This study aimed to uncover the cardioprotective mechanisms of GYPs related to mitochondrial regulation.

**Methods:**

The bioactive components in GYPs and the potential targets in treating HF were obtained and screened using the network pharmacology approach, followed by drug-disease target prediction and enrichment analyses. The pharmacological effects of GYPs in cardioprotection, mitochondrial function, mitochondrial quality control, and underlying mechanisms were further investigated in Doxorubicin (Dox)-stimulated H9c2 cardiomyocytes.

**Results:**

A total of 88 bioactive compounds of GYPs and their respective 71 drug-disease targets were identified. The hub targets covered MAPK, EGFR, PI3KCA, and Mcl-1. Enrichment analysis revealed that the pathways primarily contained PI3K/Akt, MAPK, and FoxO signalings, as well as calcium regulation, protein phosphorylation, apoptosis, and mitophagy process. In Dox-stimulated H9c2 rat cardiomyocytes, pretreatment with GYPs increased cell viability, enhanced cellular ATP content, restored basal oxygen consumption rate (OCR), and improved mitochondrial membrane potential (MMP). Furthermore, GYPs improved PINK1/parkin-mediated mitophagy without influencing mitochondrial fission/fusion proteins and the autophagic LC3 levels. Mechanistically, the phosphorylation of PI3K, Akt, GSK-3β, and the protein level of Mcl-1 was upregulated by GYP treatment.

**Conclusion:**

Our findings reveal that GYPs exert cardioprotective effects by rescuing the defective mitophagy, and PI3K/Akt/GSK-3*β*/Mcl-1 signaling is potentially involved in this process.

## Introduction

Heart failure (HF), a multi-faceted syndrome, is characterized by systolic or diastolic cardiac malfunction, as well as significant morbidity and mortality worldwide. Because of HF’s phenotypic heterogeneity and mechanistic diversity, its therapeutic options in the clinic setting are limited (Mascolo et al., 2022). Increasing evidence suggests that mitochondrial dysfunction is a mutual hallmark of heterogeneous HF caused by various risk factors, including pressure overload, metabolic stress, aging, ischemia, and cardiotoxic drugs (Zhou & Tian, 2018). Mitochondria are highly active organelles in cardiomyocytes that direct energy metabolism and cellular fate. They are essential for cardiovascular function and defense against pathological stressors. Under pathological conditions, the disruption of mitochondrial homeostasis results in reactive oxygen species (ROS) overproduction and energy insufficiency, which further disrupts cellular and mitochondrial homeostasis in a vicious circle (Zhou & Tian, 2018). Improving mitochondrial homeostasis has been preferred as a novel therapeutic approach in HF (Sabbah, 2020).

Doxorubicin (Dox) is an effective chemotherapeutic agent with severe cardiotoxicity. The mechanisms underlying Dox-induced cardiotoxicity are intricate, multifactorial, and involve processes such as the generation of free radicals, disruption of calcium homeostasis, induction of apoptosis, and impairment of mitochondria. Interruptions with different mitochondrial processes is chief among the cellular and molecular determinants of Dox-induced cardiotoxicity and HF (Wallace, Sardao & Oliveira, 2020; Zhou et al., 2023). Due to its cationic nature and the presence of hydrophilic and hydrophobic regions, Dox can easily traverse the cytoplasmic organelle membrane. Studies have shown that the concentration of Dox in mitochondria is approximately 100 times higher than in plasma (Tokarska-Schlattner et al., 2007). Accumulation of Dox within mitochondria leads to oxidative stress, perturbation of permeability transition pore opening, disruption of metabolic pathways and redox circuits, leading to disturbances in autophagy/mitophagy dynamics and an imbalance between cell survival and death (Maneechote, Chattipakorn & Chattipakorn, 2023; Wu, Leung & Poon, 2022).

Traditional Chinese medicine (TCM) and natural products have a long history of use in treating cardiovascular diseases. Natural compounds derived from TCM have been extensively used in the development of cardioprotective drugs (Guo et al., 2020; Xu et al., 2022). *Gynostemma pentaphyllum* (Thunb.) Makino, which belongs to the Cucurbitaceae family, is a well-known edible and medicinal plant in China, other East Asian countries, and Southeast Asia. The plant is used to make tea and other herbal concoctions. It has also traditionally been used to slow aging and treat aging-associated conditions (Phu et al., 2020; Shaito et al., 2020; Su et al., 2021). Gypenosides (GYPs) are the primary active constituents of *G. pentaphyllum* and contain more than 200 dammarane-type triterpenoid saponins with well-defined chemical structures. Other investigations, focusing on different cell lines or animals, indicated that GYPs possess pleiotropic properties, including anti-cancer, lipid-regulating, neuroprotective, and cardioprotective activities (Chen et al., 2022; Huang et al., 2022; Liu et al., 2021; Wang et al., 2022; Xie et al., 2024; Zhang et al., 2018; Zhi et al., 2023). Recent studies further revealed the potency of GYPs in preserving mitochondrial function when faced with cardiovascular risk factors (Song et al., 2020; Su et al., 2022; Yu et al., 2016a; Yu et al., 2016b). However, the cardioprotective mechanisms of GYPs, especially in controlling mitochondrial homeostasis, still need to be clarified.

**Figure 1:**
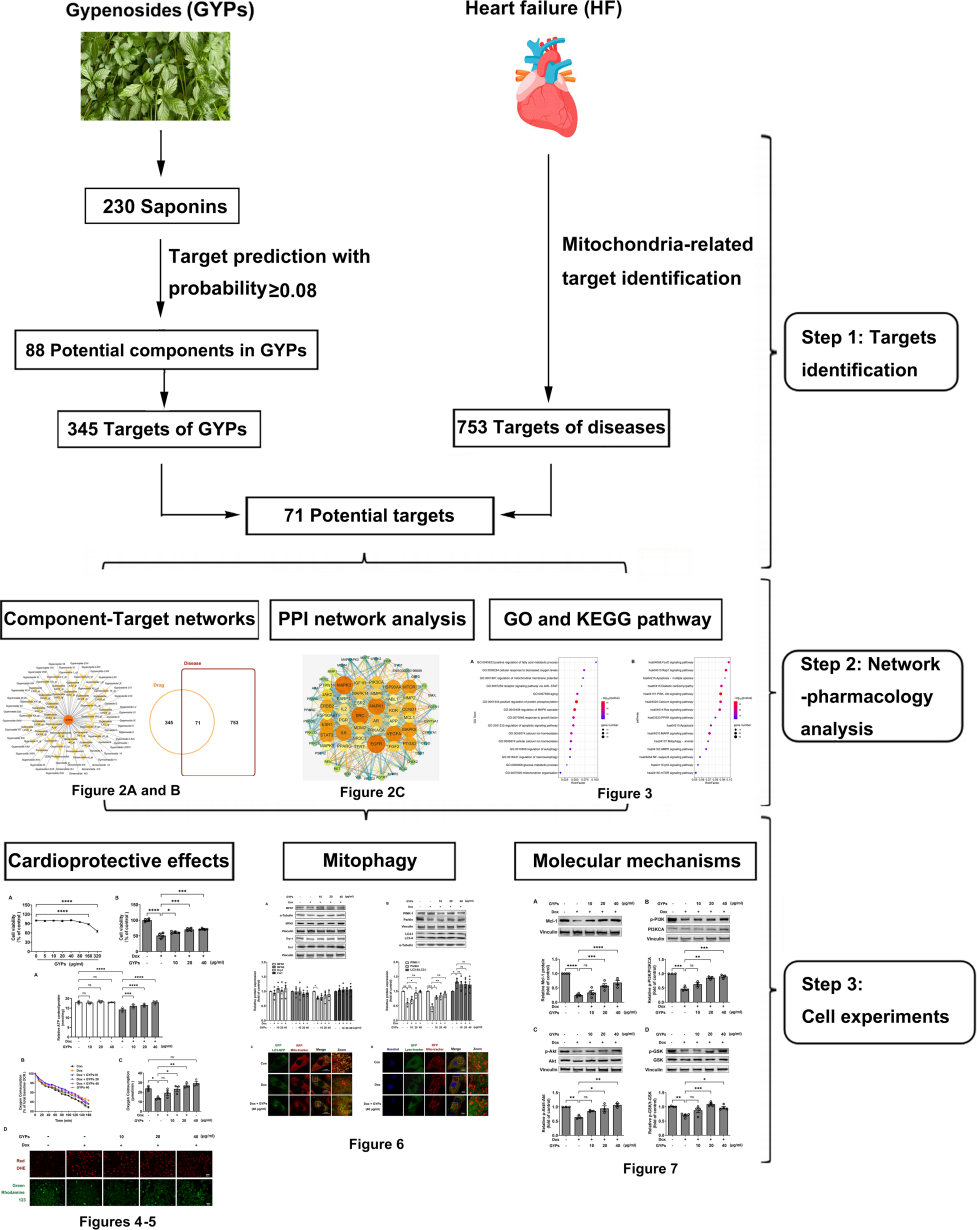
Schematic diagram of this study. *G. pentaphyllum* and heart. Image source credit: Freepik (https://www.freepik.com/).

This study integrated network pharmacology and cell research to explore the cardioprotective mechanisms of GYPs related to mitochondrial regulation. The current work revealed that GYPs exerted cardioprotective effects by rescuing the impaired mitophagy in cardiomyocytes, and the phosphatidylinositol-4,5-bisphosphate 3-kinase (PI3K)/Akt/glycogen synthase kinase 3β (GSK-3β)/myeloid cell leukemia-1 (Mcl-1) signaling might be a potential mechanism involved. The flowchart of this study is shown in Fig. 1.

## Materials & Methods

### Materials and reagents

Rat (*Rattus norvegicus*) heart/myocardium cells (H9c2; #CRL-1446, ATCC) were purchased from Hunan Fenghui Biotechnology Co., Ltd. (Changsha, China). GYPs (lot number J0423AS, specification ≥ 98%) were purchased from Meilunbio Biotechnology Co., Ltd. (Dalian, China). Polyvinylidene difluoride (PVDF) membrane was purchased from Millipore Corporation (Billerica, MA, USA). The extracellular oxygen consumption rate (OCR) plate assay kit was purchased from Dongren Chemical Technology Co., Ltd. (Shanghai, China). Dox, CCK-8 assay kit, luciferase-based ATP detection kit, adenovirus (Ad)-LC3-GFP, fluorescent dyes including rhodamine 123, dihydroethidium (DHE), MitoTracker Red CMXRos, and LysoTracker Green, as well as antibodies including anti-dynamin-related protein-1 (Drp-1), anti-fission protein 1 (Fis1), anti-mitofusin 1 (MFN1), anti-mitofusin 2 (MFN2), anti-PTEN-induced putative kinase 1 (PINK-1), anti-parkin, anti-myeloid cell leukemia-1 (Mcl-1), and anti-LC3II/I, were purchased from Beyotime Biotechnology Co., Ltd. (Shanghai, China). Anti-phosphatidylinositol-4,5-bisphosphate 3-kinase catalytic subunit alpha (PI3KCA), anti-p-PI3K, anti-Akt, anti-p-Akt (Ser473), and anti-vinculin polyclonal antibodies were purchased from ABclonal Technology Co., Ltd (Wuhan, China). Anti-GSK-3β and anti-p-GSK-3β (Ser9) monoclonal antibodies were purchased from Proteintech Group, Inc. (Wuhan, China). Additionally, anti-α-tubulin monoclonal antibodies were purchased from Cell Signaling Technology, Inc. (Danvers, MA, USA).

### Identifying constituents in GYPs and prediction of targets

Information on the monomeric saponins in GYPs was gathered from the indicated databases. The TCM systems pharmacology database (TCMSP, https://www.tcmsp-e.com/load_intro.php?id=43) was used primarily for screening bioactive compounds, while PubMed (https://pubmed.ncbi.nlm.nih.gov/) and China National Knowledge Infrastructure (CNKI, https://www.cnki.net/) databases were used for manual verification and supplementation. Potential targets of GYPs were predicted by importing the SDF files of the collected components into the SwissTargetPrediction database (http://www.swisstargetprediction.ch/) and limiting the research object to homo sapiens. Targets with a probability of ≥ 0.08 were reserved.

### Network construction and enrichment analyses

The term “heart failure and mitochondria” was used to build the search query in two databases, Genecards (https://www.genecards.org/) and Online Mendelian Inheritance in Man (OMIM, https://omim.org/), in order to retrieve mitochondria-related targets in HF. The protein names were converted to the corresponding gene names using the Universal Protein database (UniProt, https://www.uniprot.org/). The disease targets were then crossed with the drug targets to screen out the potential targets of GYPs in regulating mitochondrial homeostasis during HF progression. To better understand the molecular determinants, we used the String 11.0 database (https://cn.string-db.org/) to analyze protein-protein interaction (PPI) relationships. The PPI network was built using Cytoscape 3.8.1.

Gene ontology (GO) and Kyoto Encyclopedia of Genes and Genomes (KEGG) pathway enrichment analyses of the drug-disease targets were conducted using Metascape’s functional annotation tool (https://metascape.org/gp/index.html#/main/step1), with thresholds of *P* < 0.01. 138 The target-pathway network map was constructed using Cytoscape 3.8. 1.

### Cell culture, drug treatment, and sample collection

H9c2 rat cardiomyocytes were cultured in Dulbecco’s modified Eagle’s medium (DMEM) supplemented with 10% fetal bovine serum (FBS) under 5% CO_2_ at 37 °C. After pretreatment with GYPs for 1 h, the cells were treated with 0.5 µmol/L of Dox. The following experiments were conducted after 12 h: ATP measurement, assessment of mitochondrial membrane potential (MMP) and reactive oxygen species (ROS), determination of OCR, staining for mitochondria/lysosomes, and western blot analysis. Cell viability was determined after 24 h using the CCK-8 assay kit and adjusted to the control group.

### Quantification of ATP content

ATP concentration was determined using a firefly luciferase-based kit and measured on a multimode microplate reader (PerkinElmer, Waltham, MA, USA). The total ATP level was expressed as nmol/mg protein.

### High content screening determination of MMP and intracellular ROS

H9c2 cells were loaded with 0.5 µmol/L of rhodamine 123 at 37 °C for 30 min to indicate the MMP. In addition, the DHE fluorescent dye was used to measure the intracellular ROS levels. After drug treatment, the H9c2 cells were incubated with 10 µmol/L of DHE for 30 min. Images of fluorescently labeled cells were then captured and analyzed using the CELENA^®^ X High Content Image System (Logos Biosystems, Anyang-Si, Korea).

### Measurement of oxygen consumption

H9c2 cells were cultured in a 96-well black bottom plate (10^4^ cells/well). After 1 h of pretreatment with GYPs, the cells were treated with 0.5 µmol/L of Dox for 12 h. Basal oxygen consumption was determined according to the instruction manual of the extracellular OCR plate assay kit (Dojindo E297). The fluorescent signals (Ex: 500 nm, Em: 650 nm) were recorded on a multimode microplate reader every ∼10 min (PerkinElmer, Waltham, MA, USA). OCR was calculated using an analysis of the kinetic profiles acquired from measurements (Saito et al., 2023).

### Detection of mitophagy

Co-localization of autophagosomes and mitochondria serves as an indicator of mitophagy. In this study, Ad-LC3-GFP was applied 24 h before the drug administration, MOI = 5. Cells were then stained with 40 nmol/L of MitoTracker Red CMXRos at 37 °C for 30 min. In addition, MitoTracker Red CMXRos and LysoTracker Green (50 nmol/L) were co-loaded into H9c2 cells for 30 min to diagnose mitochondria-lysosome co-localization. The images were taken using a laser scanning confocal microscope (Nikon, Tokyo, Japan).

### Western blot analysis

Total protein was extracted from H9c2 cells and quantitated by bicinchoninic acid assay. A fixed amount of the extracted protein was separated by 10–12% SDS-PAGE gel electrophoresis (120 V, 60 min) and then transferred to PVDF membranes (220 mA, 70 min). Following blocking at room temperature with 5% skimmed milk for 1 h, the membranes were incubated with primary antibodies at 4 °C overnight and then labeled with horseradish peroxidase-conjugated secondary antibodies for 1 h at room temperature. Immunoreactive bands were visualized using the enhanced chemiluminescent substrate. Rabbit anti-Drp-1, anti-Fis1, anti-MFN1, anti-MFN2, anti-PINK-1, anti-parkin, anti-Mcl-1, anti-LC3II/I, anti-PI3KCA, anti-p-PI3K, anti-Akt, anti-p-Akt anti-GSK-3β, and anti-p-GSK-3β antibodies were used as primary antibodies. Mouse anti- *α*-tubulin or anti-vinculin antibody served as the loading control for the whole cell lysis.

### Statistical analysis

Data are presented as mean ± SEM. Statistical analysis was performed in SPSS (version 13.0, IBM) using a one-way analysis of variance (ANOVA), followed by the Bonferroni *post hoc* test. Differences were considered statistically significant at *P* < 0.05. Graphs were generated using Graph Pad Prism software 9.5.1.

## Results

### Identifying the bioactive compounds in GYPs

Using the TCMSP, CNKI, and PubMed databases, we identified 230 saponins in *G. pentaphyllum*. Potential targets of GYPs were predicted using the SwissTargetPrediction database with a probability of ≥ 0.08. Of these, 142 components were excluded when matching targets were not found in the SwissTargetPrediction database. Finally, 88 potential components were selected for further analysis (Table 1). The SwissTargetPrediction database predicted 345 potential targets of GYPs.

**Table 1:** Information of the bioactive compounds in GYPs.

Number	Molecule name	OB/%	DL	Number	Molecule name	OB/%	DL
1	Gypenoside XXXVI_qt	37.85	0.78	43	Ginsenoside rd_qt	12.23	0.77
2	Gypenoside XXXV_qt	37.73	0.78	44	Gypenoside LV_qt	12.14	0.78
3	Gypentonoside A_qt	36.13	0.80	45	Gypenoside LII_qt	11.23	0.80
4	Panaxadiol	33.09	0.79	46	Gypenoside XIX	9.13	0.29
5	Gypenoside XXVIII_qt	32.08	0.74	47	Gypenoside VIII	8.08	0.22
6	Gypenoside XXVII_qt	30.21	0.74	48	Gypenoside IV	7.81	0.04
7	Gypenoside LVIII_qt	29.69	0.79	49	Gypenoside LXIX	7.73	0.04
8	Gypenoside LXXIII_qt	29.69	0.78	50	Gypinoside LXVII	7.65	0.04
9	Gynsenoside rd_qt	29.69	0.77	51	Gypenoside I	7.36	0.02
10	Gypnoside V_qt	29.69	0.77	52	Gypenoside II	7.27	0.03
11	Gypenoside I_qt	29.69	0.77	53	Gypenoside XXIV	6.52	0.10
12	Gypenoside XIV_qt	29.69	0.77	54	Ginsenoside rb1	6.29	0.04
13	Malonylginsenoside rd_qt	29.69	0.77	55	Gypenoside VI	6.23	0.04
14	Ginsenoside rb3_qt	29.65	0.81	56	Gypenoside LXII	6.18	0.04
15	Gypenoside XLI_qt	29.65	0.78	57	Gypnoside V	6.07	0.04
16	Gypenoside L_qt	29.61	0.78	58	Ginsenoside rb2	6.02	0.04
17	Gypenoside XXX_qt	29.40	0.78	59	Ginsenoside rb3	6.02	0.04
18	Gypenoside XVIII_qt	29.40	0.77	60	Gypenoside XVIII	6.00	0.10
19	Gypenoside XL_qt	29.29	0.79	61	Gypenoside XLVII	6.00	0.04
20	Gypenoside LXXI_qt	29.16	0.78	62	Gypenoside LVI	5.96	0.04
21	Gypenoside XXV_qt	28.89	0.80	63	Gypinoside III	5.95	0.04
22	Gypsogenin	26.77	0.75	64	Gypenoside LXIII	5.92	0.04
23	Gypenoside LXVIII_qt	20.59	0.80	65	Gypenoside XXII	5.86	0.04
24	Gypenoside XXIX_qt	23.90	0.78	66	Gypenoside XVI	5.81	0.05
25	Gypinoside LXVII_qt	20.18	0.79	67	Gynsenoside rd	5.60	0.09
26	Gynosaponin TN-1_qt	20.13	0.79	68	Gypenoside XI	4.89	0.10
27	Gypenoside LI_qt	20.13	0.79	69	Gypenoside LXIV	4.79	0.11
28	Gypenoside LX_qt	20.13	0.79	70	Gypenoside IX	4.76	0.11
29	Gypenoside XXXIX_qt	20.13	0.79	71	Gypenoside LV	4.75	0.11
30	Gypenoside XXXII_qt	19.80	0.79	72	Gypenoside LXXVI	4.15	0.60
31	Gypenoside XX_qt	19.49	0.80	73	Gypenoside LII	3.78	0.10
32	Gypenoside LXI_qt	19.27	0.81	74	Gypenoside VII	3.78	0.09
33	Gypenoside LX	18.78	0.29	75	Gypenoside XXV	3.59	0.10
34	Gypenoside LXV	18.14	0.29	76	Gypenoside XVII	3.51	0.10
35	Gypenoside XV	17.74	0.05	77	Gypinoside VII	3.01	0.04
36	Gypenoside LVII	17.74	0.12	78	Ginsenoside rh2	–	–
37	Gypenoside XLVI	17.74	0.10	79	Ginsenoside rd	–	–
38	Gypenoside XXI	17.63	0.30	80	Gymnemaside VI	–	–
39	Gypenoside XLVIII_qt	13.63	0.80	81–85	Gynoside A-E	–	–
40	Gypenoside VIII_qt	13.42	0.79	86	Gypenoside VN2	–	–
41	Gypenoside LXV_qt	13.03	0.78	87	Protopanaxatriol	–	–
42	Gypenoside III_qt	12.81	0.80	88	(20S)-Protopanaxadiol	–	–

**Notes.**

GYPs, Gypenosides; OB, Oral bioavailability; DL, Drug-likeness.

**Figure 2:**
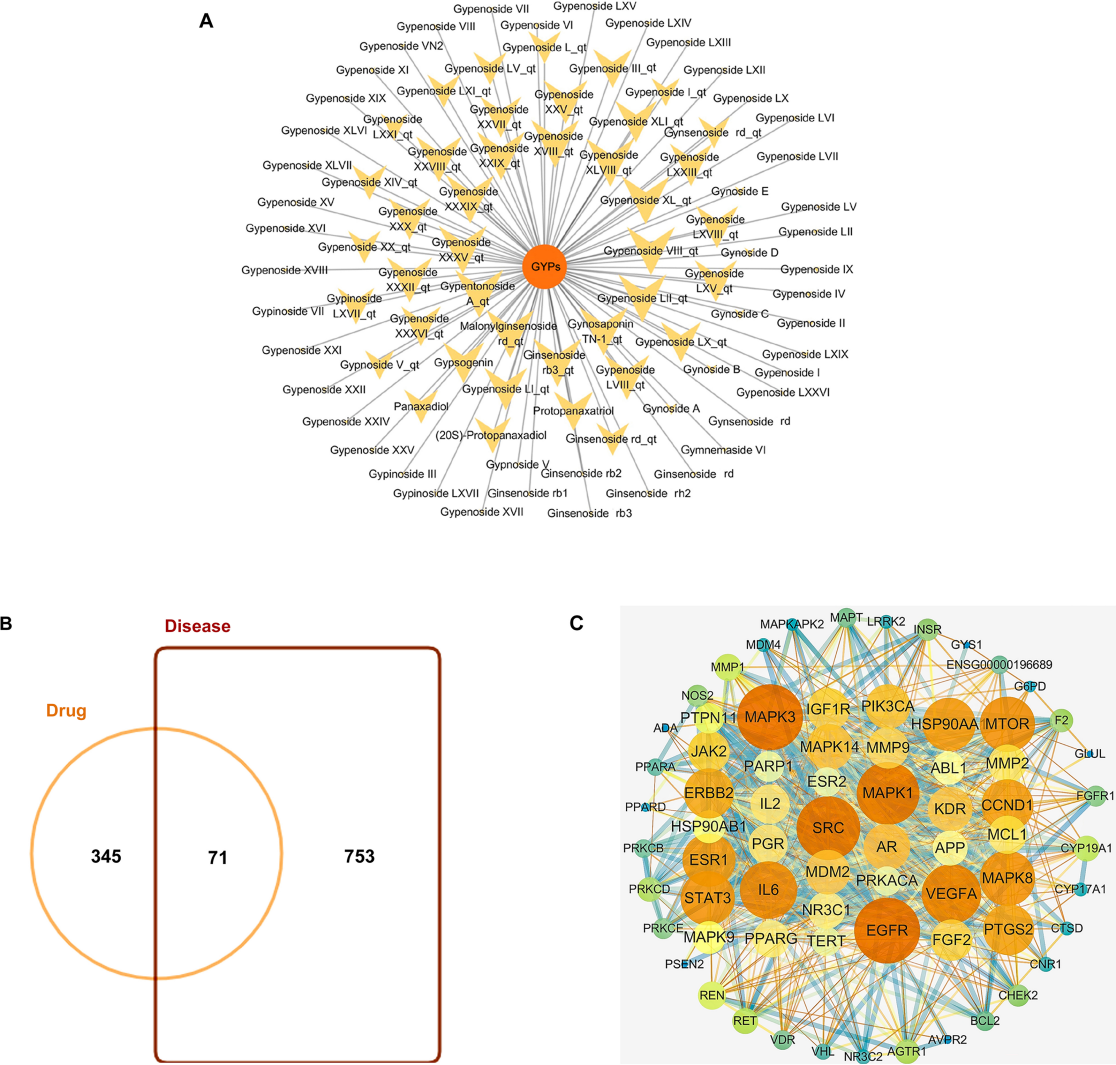
Bioactive compounds in GYPs, and Venn diagram of the intersecting drug-disease targets and PPI networks. (A) The bioactive compounds in GYPs. The size of the yellow dots represents the number of the predicted drug targets. (B) Venn diagram of intersecting targets of GYP on mitochondria against HF. (C) PPI network of compound-disease targets.The nodes vary in dimension and color, displayed in descending order of degree values from large to small and warm to cool, respectively.

GYPs have high molecular weights and polarity due to the presence of one or more monosaccharide molecules, which negatively affect their oral bioavailability (OB) and drug-likeness (DL) scores. Some components, such as Gypnoside V_qt, Gypenoside I_qt, Ginsenoside rb3_qt, and Gypenoside XVIII_qt, shown in Table 1 and Fig. 2A, are derived from native saponins that release monosaccharides through the hydrolysis of glycosidic bonds. These sapogenins exhibit better oral OB and DL than their corresponding saponins while targeting more potential targets.

### Identifying drug-disease targets and PPI networks

HF-targeted genes related to mitochondria were retrieved from Genecards and OMIM. Based on the correlation scores, 753 targets were recognized as disease targets. The 753 disease targets were mapped to the 345 potential targets of GYPs to obtain the overlapping targets. As shown in Fig. 2B, 71 targets were confirmed as drug-disease candidates for GYPs.

The PPI network revealed 886 interactions between the 71 drug-disease targets, with 38 nodes in the circle’s center (Fig. 2C). Some targets reflected higher degrees of involvement, including mitogen-activated protein kinase (MAPK), epidermal growth factor receptor (EGFR), PI3KCA, Mcl-1, vascular endothelial growth factor (VEGF), and peroxisome proliferator-activated receptor gamma (PPAR *γ*), *etc*. These protein targets may account for the mitochondrial actions of GYPs in treating HF.

### GO enrichment and KEGG pathway analyses

**Figure 3:**
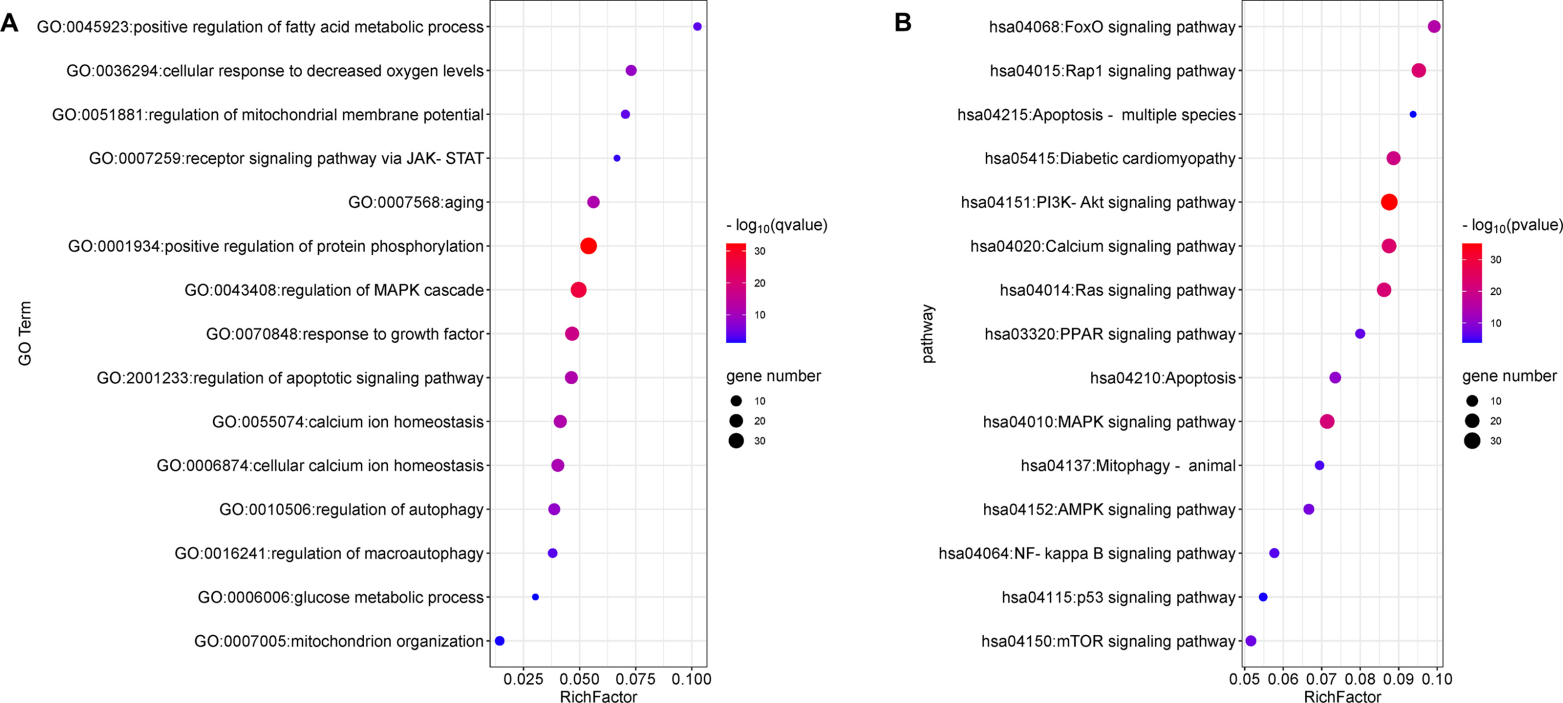
Enrichment analyses of the potential targets. GO enrichment (A) and KEGG pathway analyses (B) of the drug-disease targets. The *Q* value is an adjusted *P* value representing enrichment significance, and the size of the nodes represent the counts of genes. The rich factor represents the ratio of the differentially expressed genes to the total number of all annotated genes located in the pathway.

The top 15 significant terms of GO enrichment analysis are shown in Fig. 3A, and indicate that the regulation of protein phosphorylation, cellular calcium ion homeostasis, MMP, autophagy, and response to growth factors were involved in the cardioprotective activity of GYPs. In addition, the top 15 signal pathways of KEGG analysis are depicted in Fig. 3B. The majority were involved with PI3K/Akt, MAPK, Ras, and transcription factor forkhead box O (FoxO) signaling pathways, as well as signaling pathways regulating calcium, apoptosis, and mitophagy. Accordingly, the following experiments verify the specific mechanisms of GYPs on HF, mainly from the perspective of the indicated process and pathways. The effects of GYPs on mitochondrial function, membrane potential, dynamics, and mitophagy were also evaluated to identify pivotal processes, followed by an investigation into the associated targets and signaling pathways.

### GYPs protect H9c2 rat cardiomyocytes from Dox-induced cytotoxicity

**Figure 4:**
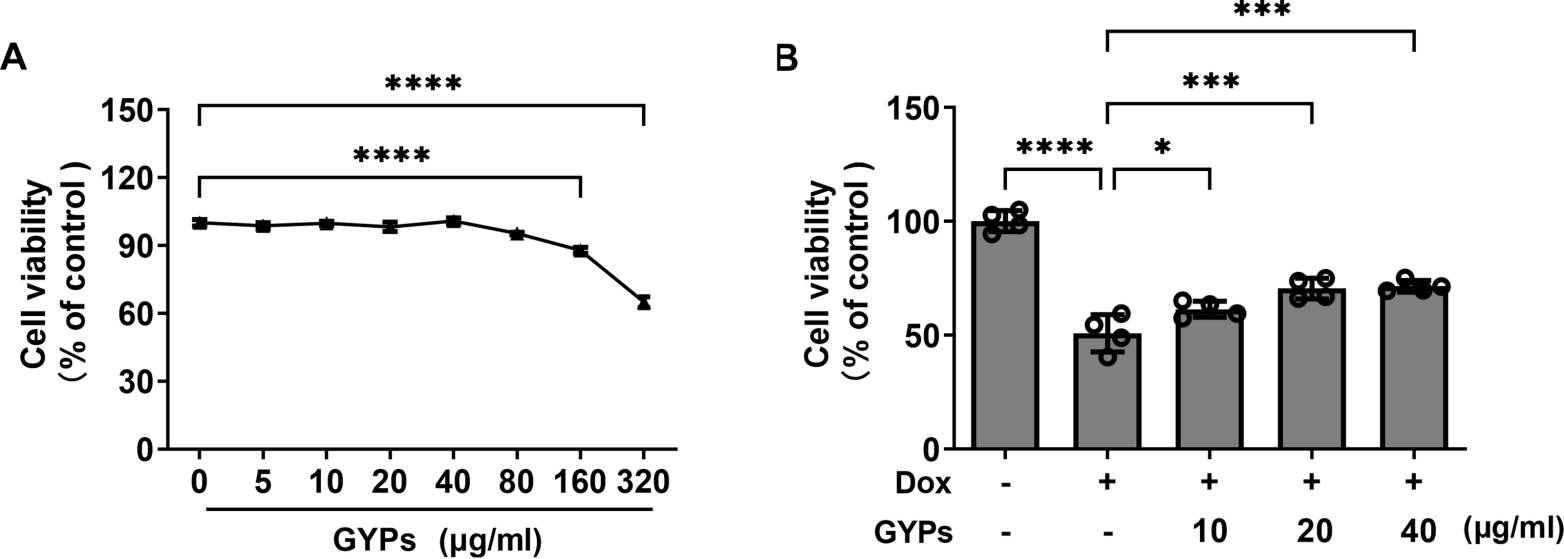
GYPs decrease Dox-induced cytotoxicity in H9c2 rat cardiomyocytes. (A) Cultured H9c2 rat cardiomyocytes were treated with GYPs of 5, 10, 20, 40, 80, 160, and 320 *μ*g/ml for 24 h, respectively. Cell viability was analyzed by CCK8 assay, *n* = 6. (B) H9c2 cells were pretreated with 10, 20, and 40 *μ*g/ml GYPs for 1 h and then exposed to 0.5µmol/L of Dox. After 24 h, cell viability was analyzed by the CCK8 assay, *n* = 4. Data were represented in means ± SEM.*P* values were calculated by one-way ANOVA, *P < 0.05, **P < 0.01, ***P < 0.001, ****P < 0.0001.

Cell experiments were performed to evaluate the effects of GYPs on cardioprotection and mitochondrial regulation. The drug concentrations of GYPs typically range from five to 75 µg/ml according to previous research (Hu et al., 2022; Tu et al., 2021). Consistently, the CCK8 assay (Fig. 4A) showed that GYPs did not reduce the viability of H9c2 rat cardiomyocytes at concentrations below 80 µg/ml. Therefore, the non-toxic concentrations of GYPs at 10–40 µg/mL were applied in this study.

It is well-known that the anthracycline antitumor agent Dox can induce cardiotoxicity, leading to cardiomyocyte death and HF (Kitakata et al., 2022). Compared to the control group, Dox reduced cell viability by approximately 50%, which was alleviated by GYP pretreatment at 10, 20, and 40 µg/ml, respectively (Fig. 4B).

### GYPs ameliorate Dox-induced mitochondrial dysfunction in H9c2 rat cardiomyocytes

**Figure 5:**
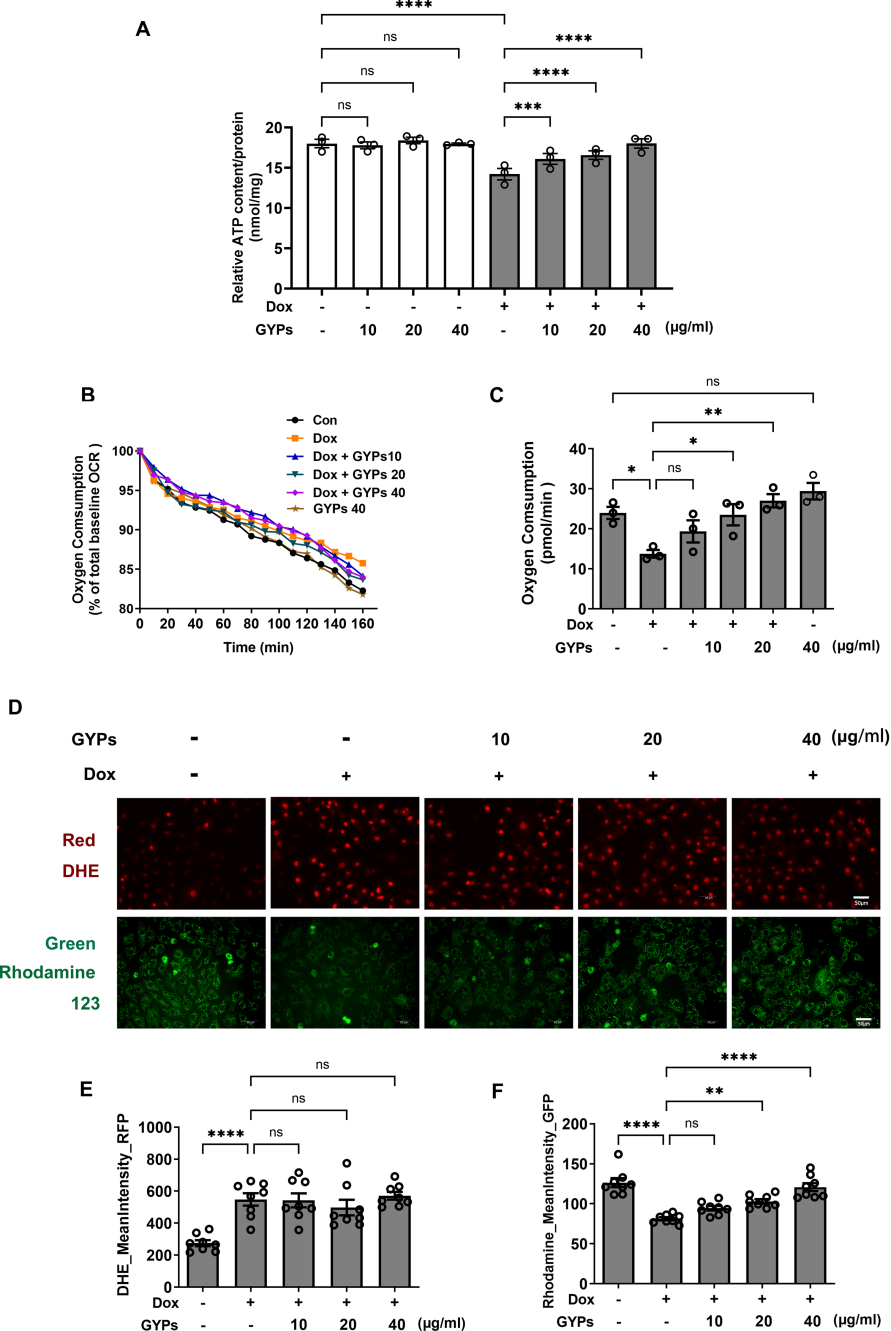
GYPs attenuate Dox-induced mitochondrial dysfunction without affecting the oxidative stress state. Cultured H9c2 rat cardiomyocytes were exposed to Dox for 12 h with and without GYP pretreatment. (A) ATP content was detected and adjusted to nmol/mg protein, *n* = 3. (B) Basal oxygen consumption was measured on a multimode microplate reader every 10 min. (C) OCR of 10^4^ cells was calculated using an analysis of the kinetic profiles acquired from the measurements, *n* = 3. (D) Cells were stained with DHE (red) or rhodamine 123 (green), respectively, and photographed by CELENA^®^ X High Content Image System (200 ×). The scale bars represent 50 µm. The mean DHE fluorescence intensities (E) and MMP (F) of 15 fields in each well were analyzed automatically, *n* = 8. Data were represented in means±SEM. *P* values were calculated by one-way ANOVA, *P < 0.05, **P < 0.01, ***P < 0.001, ****P < 0.0001.

Mitochondria integrate fuel metabolism to generate energy in the form of ATP. Mitochondrial dysfunction and oxidative stress have been implicated in Dox cardiomyopathy (Wu, Leung & Poon, 2022). In this study, we observed significant loss of ATP production, basal oxygen consumption, and MMP in Dox-treated H9c2 cells with increased production of ROS. GYPs did not affect mitochondrial function under basal conditions but could improve the cellular ATP, OCR, and MMP in Dox-stimulated H9c2 cells. Interestingly, GYPs showed no effects on Dox-induced ROS overproduction, indicating that GYPs had no anti-oxidative potency in cultured rat cardiomyocytes (Fig. 5).

### GYPs alleviate the mitophagy block when facing Dox stimulation

**Figure 6:**
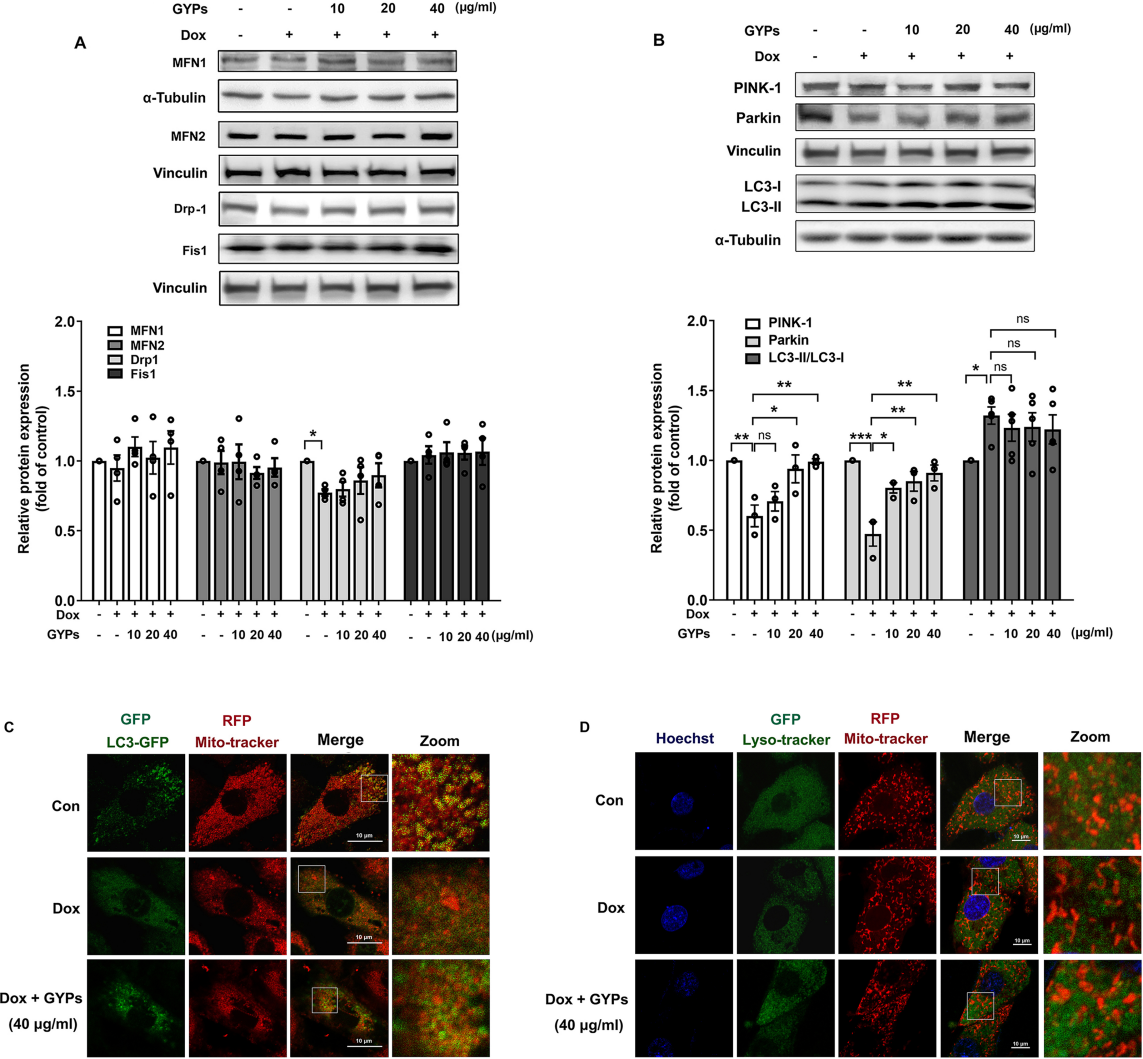
GYPs ameliorate mitophagy block in Dox-stimulated H9c2 cells. Cultured H9c2 cells were exposed to Dox with and without GYP pretreatment. After 12 h, the mitochondrial fusion and fission proteins MFN1, MFN2, DRP-1, and Fis1 (A), as well as mitophagy- and autophagy-related proteins PINK-1, parkin, and LC3 II / I (B), were detected by Western blot analysis. Mitophagy was inspected by a confocal microscope, analyzing the co-localization of autophagosomes (C) and lysosomes (D) with mitochondria, respectively. The scale bars represent 10 µm. Experiments were repeated three or four times. The values were represented in means ±SEM. *P* values were calculated by one-way ANOVA, *P < 0.05, **P < 0.01, ***P < 0.001.

Mitochondria are highly dynamic organelles. Appropriate management of mitochondrial biogenesis, fission/fusion, and mitophagy is essential for preserving mitochondrial integrity and function (Fajardo et al., 2022). In our current work, we detected the expression of critical proteins in mitochondrial quality control using western blot analysis. As shown in Figs. 6A and 6B, no significant changes were observed in MFN 1/2, Drp-1, or Fis-1 after GYP administration, indicating that GYPs do not affect mitochondrial fission and fusion. Notably, pretreatment with GYPs did not affect Dox-induced LC3 II/I, but instead enhanced the protein levels of PINK-1 and parkin, which are central mitophagy priming factors. Therefore, we further detected mitophagy in Dox-stimulated H9c2 cells with and without GYP pretreatment. Fluorescence images show the co-localization of mitochondria with autophagosomes (Fig. 6C) and lysosomes (Fig. 6D). Compared to the control group, co-localizations of both mitochondria/autophagosomes and mitochondria/lysosomes were decreased by Dox administration, whereas pretreatment with GYPs elevated these co-localizations. According to these results, GYPs can alleviate the mitophagy block induced by Dox in H9c2 rat cardiomyocytes.

### GYPs increase PI3K/Akt/GSK-3β/Mcl-1 transduction in Dox-stimulated H9c2 rat cardiomyocytes

**Figure 7:**
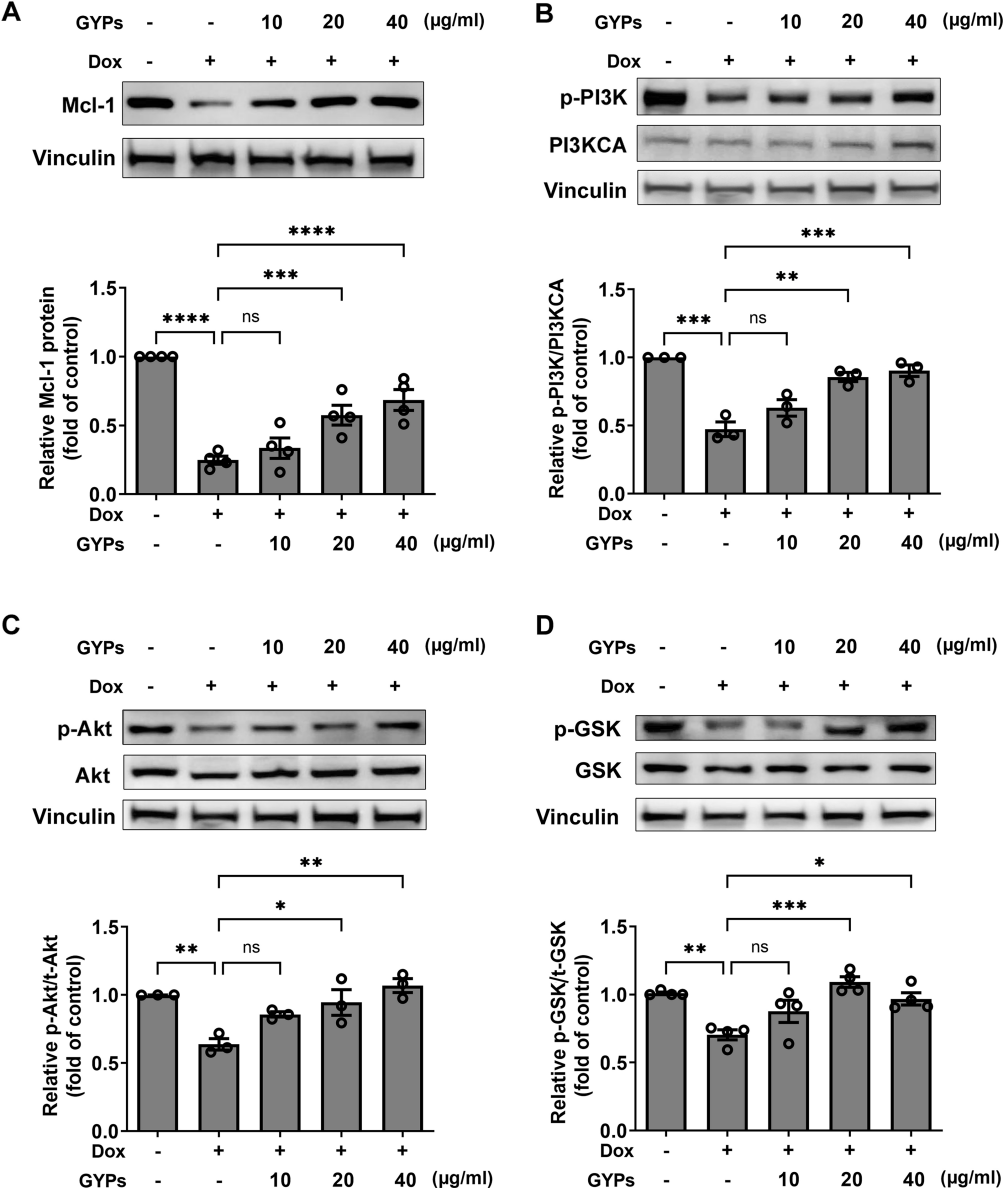
GYPs increase the Mcl-1 level and PI3K/Akt/GSK-3 *β* signaling in Dox-stimulated H9c2 cells. Cultured H9c2 cells were exposed to Dox with and without GYP pretreatment. After 12 h, the Mcl-1 protein level (A), phosphorylated PI3K and PI3KCA (B), phosphorylated and total Akt (C), as well as phosphorylated and total GSK-3 *β* (D), were detected by western blot analysis. Experiments were repeated three or four times. The values were represented in means ± SEM. *P* values were calculated by one-way ANOVA, *P < 0.05, **P < 0.01, ***P < 0.001, ****P < 0.0001.

According to our network pharmacological work, multiple molecular targets and signaling pathways may be implicated in managing the effects of GYPs. The current cell study explored whether GYPs could regulate PI3K/Akt transduction, the corresponding GSK-3β phosphorylation, and Mcl-1 decrease in Dox-stimulated cardiomyocytes. Western blot analysis showed that Dox reduced the p-PI3K and p-Akt levels, which were elevated by GYPs. In addition, GYPs pretreatment also increased p-GSK-3β and the protein level of Mcl-1 compared to the Dox group (Fig. 7). These findings, consistent with the key results from network pharmacology analysis, provide evidence that GYPs exert their effects on mitophagy and cardioprotection, probably through the PI3K/Akt/GSK-3β/Mcl-1 signaling pathway. The current data did not include other potential mechanisms, such as MAPK and EGFR, due to insignificant changes in the cell experiments or lack of research basis.

## Discussion

As the main active components of *G. pentaphyllum*, GYPs possess biological activities against various cardiovascular diseases, including myocardial infarction, atherosclerosis, diabetic cardiomyopathy, and HF. Treatment with GYPs can significantly reduce myocardial damage and alleviate cardiac dysfunction (Huang et al., 2022; Song et al., 2020; Yu et al., 2016a; Yu et al., 2016b; Zhang et al., 2018). The present work provides further cardioprotective evidence of GYPs as a mitophagy inducer, potentially *via* PI3K/Akt/GSK-3β/Mcl-1 pathway regulation.

To date, 248 dammarane-type triterpenoid saponins have been identified from *G. pentaphyllum*. The structural diversity is based on the variety of sapogenins and glycosylations (Nguyen et al., 2021; Su et al., 2021). Although most saponins do not possess ideal OB and DL scores, the oral administration of *G. pentaphyllum* or GYPs has a substantial pharmacodynamic basis and clinical data support that it potentially benefits from their biotransformation *in vivo*. The biotransformation of GYPs primarily involves deglycosylation and dehydration processes, leading to reduced molecular weight and improved lipid-water partition coefficient, thereby enhancing their OB and DL properties (He et al., 2019; Zhang et al., 2023). For instance, the hydrolysis of Gypenoside I results in the formation of Gypensapogenin I (Gypenoside I_qt), which significantly increases oral bioavailability from 7.36% to 29.69%, enhances drug-like properties from a DL value of 0.02 to 0.77, and expands the predicted number of targets from 1 to 27 (Table 1 and Fig. 1). A recent study demonstrated that Gypensapogenin I effectively mitigated myocardial damage both in *vitro* and in *vivo*, highlighting the potential of GYPs and its monomeric saponins and sapogenins for myocardial protection (Li et al., 2022).

In this study, 88 potential components were selected for network pharmacology analysis. Among them, GYP VII, GYP A, and Gypensapogenin I had experimental evidence proving their efficacy against HF. These components can protect the heart from ischemia/reperfusion and isoproterenol-induced injuries and maintain mitochondrial homeostasis (Chang et al., 2020; Su et al., 2022; Yu et al., 2021). Ginsenosides and their isomers, which comprise around 25% of the total GYPs in *G. pentaphyllum*, also have strong potency in treating HF (Lu et al., 2023). Under stress conditions, ginsenosides Rb1 (Li et al., 2017), Rb3 (Chen et al., 2019), Rg1 (Dong et al., 2016; Liu et al., 2022), Rg3 (Ni et al., 2022), and Rd (Wang et al., 2013b) can regulate mitochondrial pathway networks, thus preserving mitochondrial homeostasis and heart function. Better understanding of the mechanisms of GYPs in governing mitochondrial homeostasis will facilitate their development as cardiovascular medications.

The PPI network and enrichment analysis predicted the potential mechanisms of GYPs in managing mitochondria and treating HF. The highly enriched processes and pathways included protein phosphorylation, regulation of calcium homeostasis, response to growth factor, mitophagy, apoptosis, and signalings of MAPK, PI3K/Akt, Ras, Rap, and FOXO. The potential hub targets included EGFR, MAPK, Src, VEGF, PI3KCA, and Mcl-1. Some of them play a significant role in governing mitochondrial homeostasis processes, including protein phosphorylation, mitophagy, and calcium homeostasis; as well as signaling pathways and targets of MAPK, PI3K/Akt, FOXO, and Mcl-1 (Ajzashokouhi et al., 2023; Javadov, Jang & Agostini, 2014; Kim & Koh, 2017; Moyzis et al., 2019; Popov, 2023; Yang et al., 2017). Therefore, we further investigated the cardioprotective effect and potential mechanisms in Dox-stimulated rat cardiomyocytes based on the results from network pharmacology analysis. The embryonic cardiomyocyte cell line H9c2 is widely utilized for the investigation of cardioprotective drugs. Our present cell study shows that GYPs can preserve ATP production, mitochondrial oxygen consumption, and MMP in Dox-stimulated H9c2 cardiomyocytes. However, they do not have a defensive effect against excessive ROS. These results demonstrate that the protective role of GYPs primarily relies on preserving mitochondrial homeostasis rather than eliminating oxygen radical formation. To date, no studies have reported that GYPs exert antioxidant effects in cells and animals. The antioxidant capacity of GYPs has solely been confirmed through *in vitro* free radical scavenging assays, with drug concentrations much higher than those commonly employed in cell-based investigations (Jiang et al., 2017; Wang, Shi & Jiang, 2018).

Mitochondrial quality control systems are responsible for maintaining integrated mitochondrial structure and function, which includes mitochondrial biogenesis, mitochondrial fission/fusion dynamics, and mitophagy (Fajardo et al., 2022). Mitophagy is a specialized autophagic process that facilitates the lysosomal clearance of damaged mitochondria. Though there is currently some debate as to the role of mitophagy at different stages of HF, most research supports that moderate mitophagy is crucial for cardiac adaptations to stress by deterring the accumulation of damaged mitochondria (Morales et al., 2020; Tu et al., 2022). Facing Dox stimulation, GYPs in this study restored the PINK/parkin-mediated mitophagy. The PINK/parkin pathway controls the specific elimination of dysfunctional mitochondria. Stressors such as membrane depolarization, mitochondrial damage, and complex dysfunction result in the accumulation of PINK1 on the outer mitochondrial membrane (OMM). Subsequent homodimerization of PINK1 on the OMM causes autophosphorylation, activating itself and facilitating parkin activation along with its substrate ubiquitin. The ubiquitin-labeled mitochondria recruit autophagy factors and anchor into autophagosomes by interacting with LC3 (Georgakopoulos, Wells & Campanella, 2017). Interestingly, we found that GYPs could prevent the decline of PINK-1 and parkin without affecting the LC3 level. This finding indicates that specific molecular determinants of mitophagy are involved in the cardioprotective effect of GYPs.

The PI3K/Akt pathway is well known for its preventive roles in HF progression, supporting mitochondrial homeostasis, and cardiomyocyte survival. It coordinates multiple intracellular signals through protein phosphorylation. Activation of PI3K leads to the phosphorylation of Akt, which subsequently phosphorylates downstream targets, including GSK-3β, mTOR, FOXO, and glucose transporters (Ghafouri-Fard et al., 2022). GSK-3β is a constitutively active protein kinase with a number of physiological functions. Akt-mediated phosphorylation of serine nine residue on GSK-3β leads to its inhibition (Atkins et al., 2012). According to investigations conducted on rats with myocardial ischemia-reperfusion and ApoE^−/−^ mice, GYPs exert cardiovascular protective effects by activating the PI3K/Akt pathway (Song et al., 2020; Yu et al., 2016b). Recently, the anti-apoptotic protein Mcl-1 has been defined as a mitophagy receptor that participates in efficiently removing mitochondria through its functional LC3-interacting region motif (Cen et al., 2020). Loss of Mcl-1 in cardiomyocytes leads to rapid mitochondrial malfunction and HF development (Moyzis et al., 2020). Mcl-1 has a short half-life and is rapidly eliminated by proteasome-mediated degradation (Senichkin et al., 2020). Phosphorylation of Mcl-1 by active GSK-3β facilitates its ubiquitination and proteasomal degradation, while sustained activation of Akt withdraws this process and increases the Mcl-1 protein level (Longo et al., 2008; Senichkin et al., 2020; Wang et al., 2013a). In this sense, PI3K/Akt/GSK-3β is a potential mitophagic signaling pathway that prevents Mcl-1 degradation. Consistent with the predicted data in network pharmacology, our cell study confirmed that GYPs activated PI3K/Akt/GSK-3β transduction and increased the Mcl-1 protein level in Dox-stimulated rat cardiomyocytes. Elevated Mcl-1 may facilitate mitophagy and direct the cardioprotective effects of GYPs, which needs further investigation.

## Conclusion

In this study, we employed a multidisciplinary approach by integrating network pharmacology analysis with cell studies to investigate the cardioprotective effect and molecular mechanisms of GYPs. Our findings suggest that GYPs exert cardioprotective effects by rescuing defective mitophagy, and PI3K/Akt/GSK-3β mediated Mcl-1 elevation potentially contributes to this process. This finding expands our understanding of the cellular and molecular mechanisms underlying the cardioprotective benefits of GYPs and highlights the potential value of modulating mitophagy in heart disease interventions.

##  Supplemental Information

10.7717/peerj.17538/supp-1 Supplemental Information 1 Network pharmacology

10.7717/peerj.17538/supp-2 Supplemental Information 2 Cell experiment

10.7717/peerj.17538/supp-3 Supplemental Information 3 WB blots in Fig. 6

10.7717/peerj.17538/supp-4 Supplemental Information 4 WB blots in Fig. 7

## Abbreviations

Ad: adenovirus
DL: drug-like indexes
Dox: Doxorubicin
Drp-1: dynamin-related protein-1
EGFR: epidermal growth factor receptor
Fis 1: fission protein 1
FoxO: transcription factor forkhead box O
*G. pentaphyllum*: *Gynostemma pentaphyllum*
GO: Gene Ontology
GSK-3β: glycogen synthase kinase-3β
GYPs: Gypenosides
HCS: High content screening
HF: heart failure
KEGG: Kyoto Encyclopedia of Genes and Genomes
MAPK: mitogen-activated protein kinase
Mcl-1: myeloid cell leukemia-1
MFN: mitofusin
MMP: mitochondrial membrane potential
PINK-1: PTEN-induced putative kinase 1
PI3K: phosphatidylinositol-4,5-bisphosphate 3-kinase
PI3KCA: phosphatidylinositol-4,5-bisphosphate 3-kinase catalytic subunit alpha
OB: oral bioavailability
OCR: oxygen consumption rate
OMM: outer mitochondrial membrane
PPAR *γ*: peroxisome proliferator-activated receptor gamma
PPI: protein-protein interaction
ROS: reactive oxygen species
TCM: traditional Chinese medicine
VEGF: vascular endothelial growth factor

## Additional Information and Declarations

### Data Availability

The following information was supplied regarding data availability: The raw measurements are available in the Supplementary Files.
