# Supplementary figures and images for: Gypenosides exert cardioprotective effects by promoting mitophagy and activating PI3K/Akt/GSK-3β/Mcl-1 signaling
Source: PeerJ. 2024 Jun 20;12:e17538. doi: 10.7717/peerj.17538 (PMC11193969; doi:10.7717/peerj.17538)

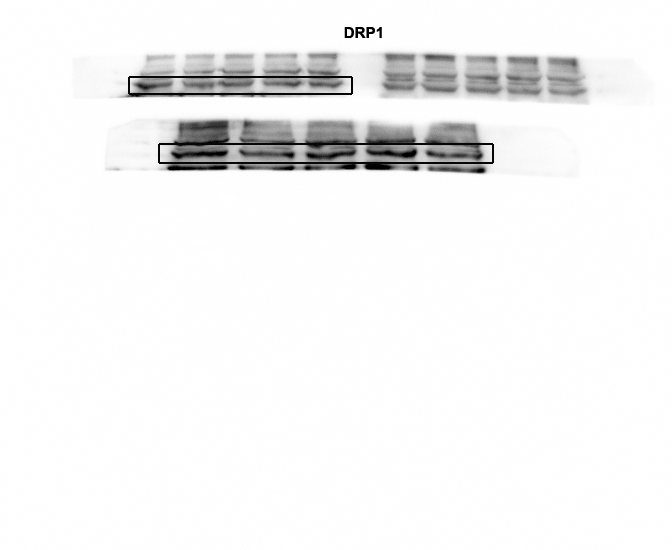

Supplement: Supplemental Information 3 [file peerj-12-17538-s003.zip › raw data-WB blots in Figure 6/drp/011-shine.tif]

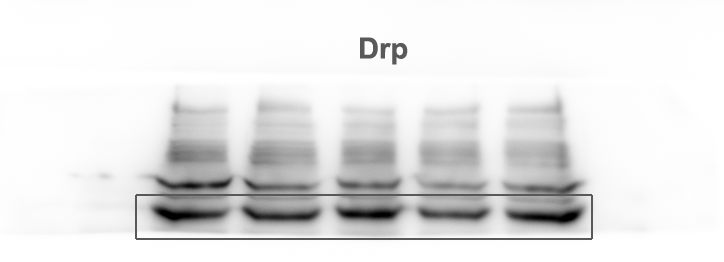

Supplement: Supplemental Information 3 [file peerj-12-17538-s003.zip › raw data-WB blots in Figure 6/drp/022-shine.tif]

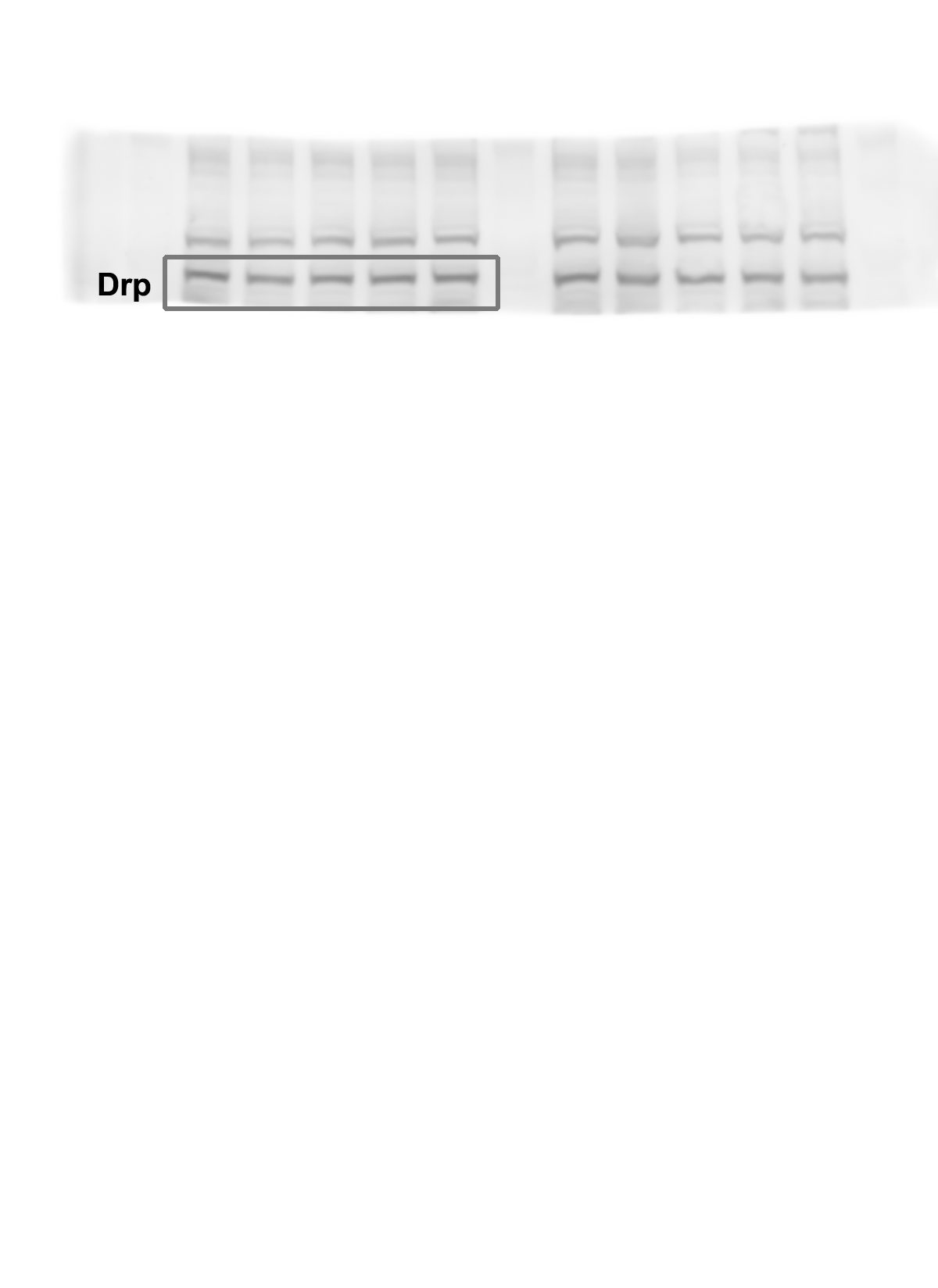

Supplement: Supplemental Information 3 [file peerj-12-17538-s003.zip › raw data-WB blots in Figure 6/drp/drp1_155615_Ch_Chemi samples 1~5.tif]

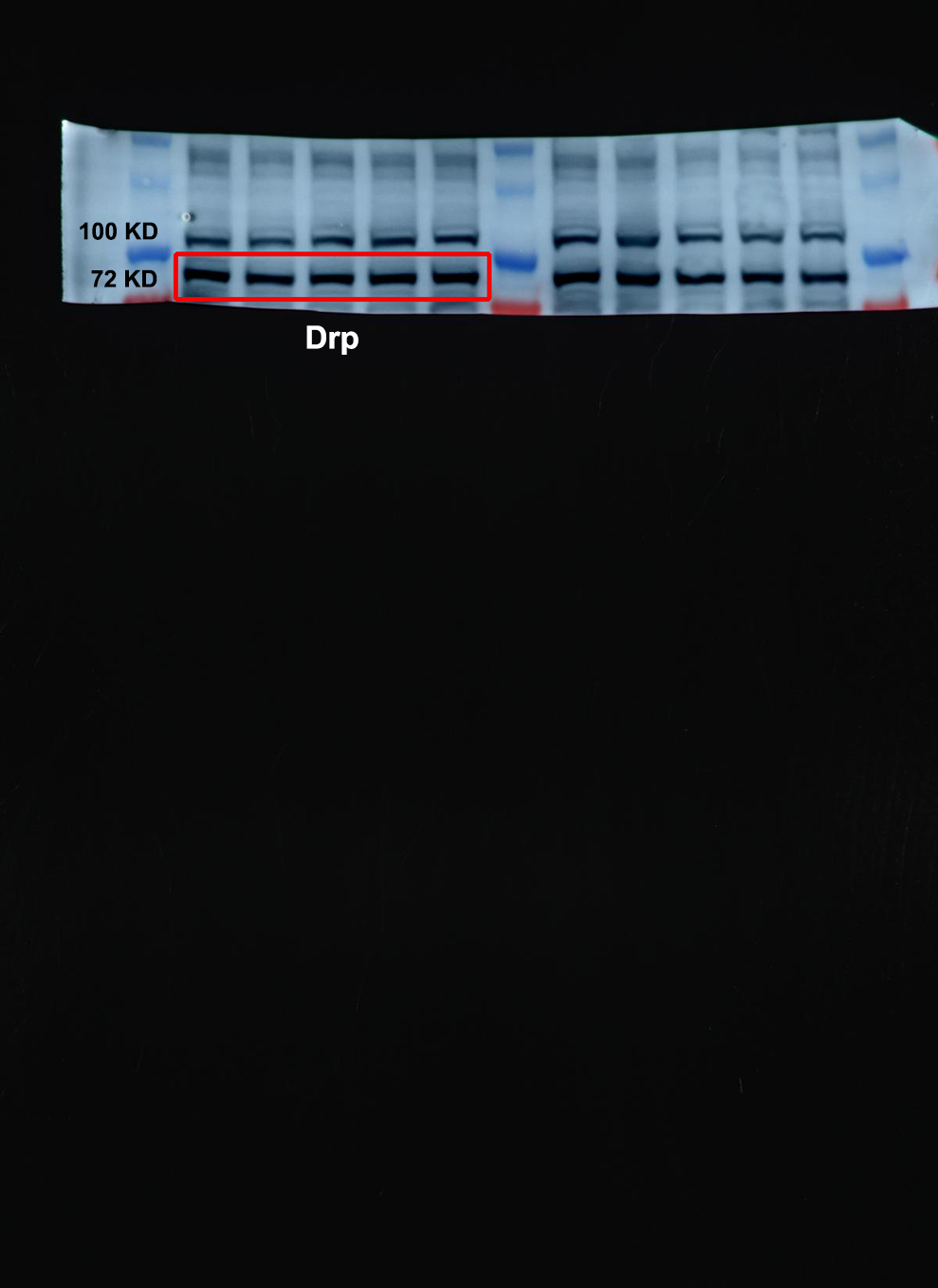

Supplement: Supplemental Information 3 [file peerj-12-17538-s003.zip › raw data-WB blots in Figure 6/drp/drp1_155615_Ch_Chemi-marker.tif]

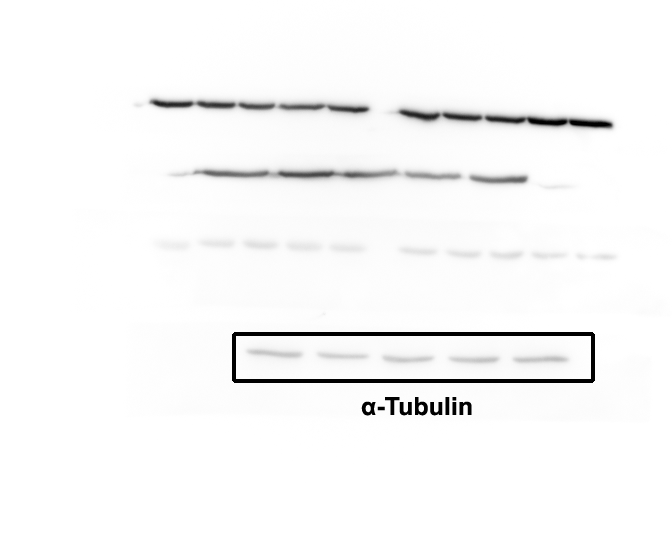

Supplement: Supplemental Information 3 [file peerj-12-17538-s003.zip › raw data-WB blots in Figure 6/drp/tubulin-below.tif]

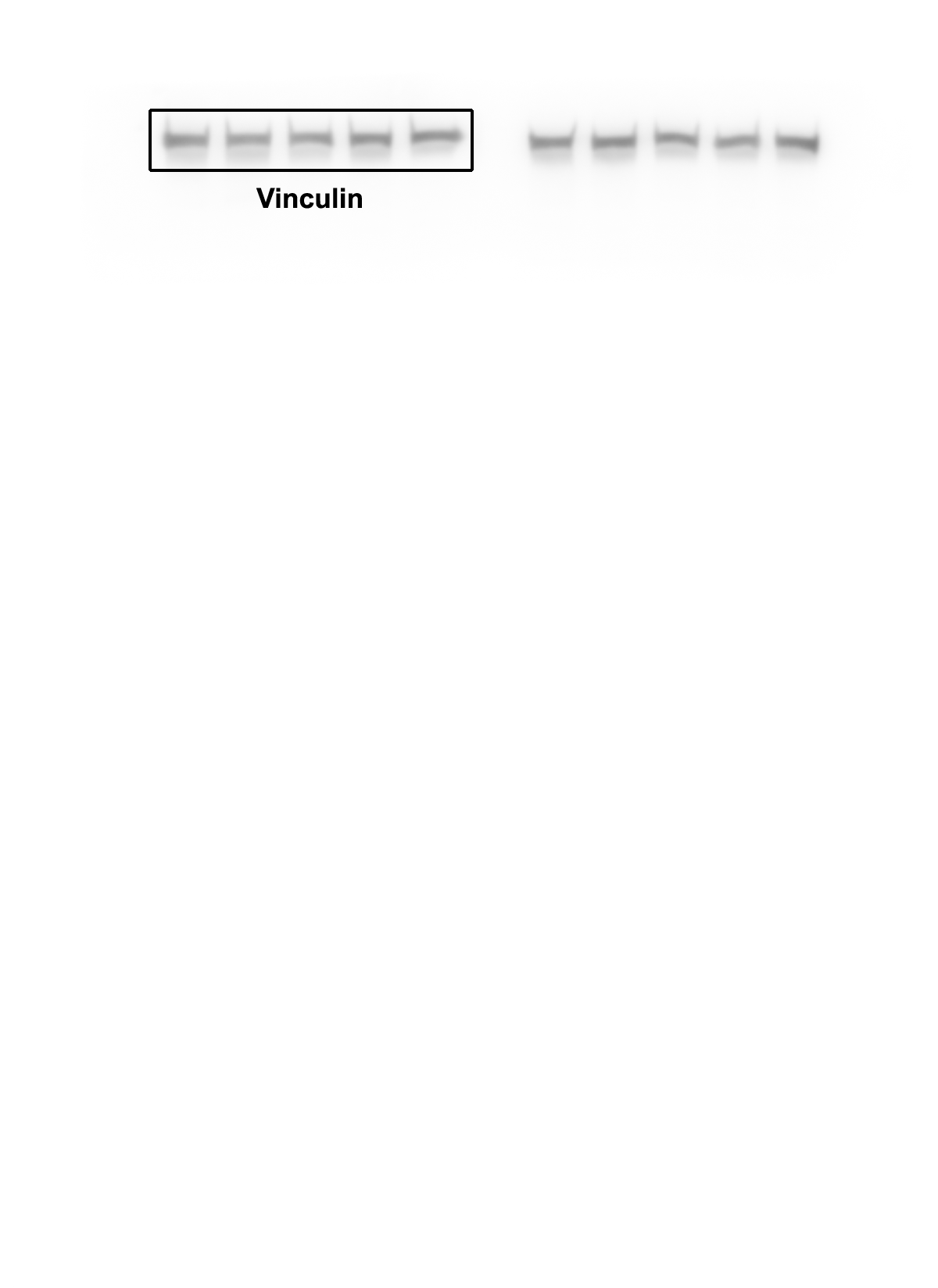

Supplement: Supplemental Information 3 [file peerj-12-17538-s003.zip › raw data-WB blots in Figure 6/drp/vinculin-c_134806_Ch_Chemi samples 1~5.tif]

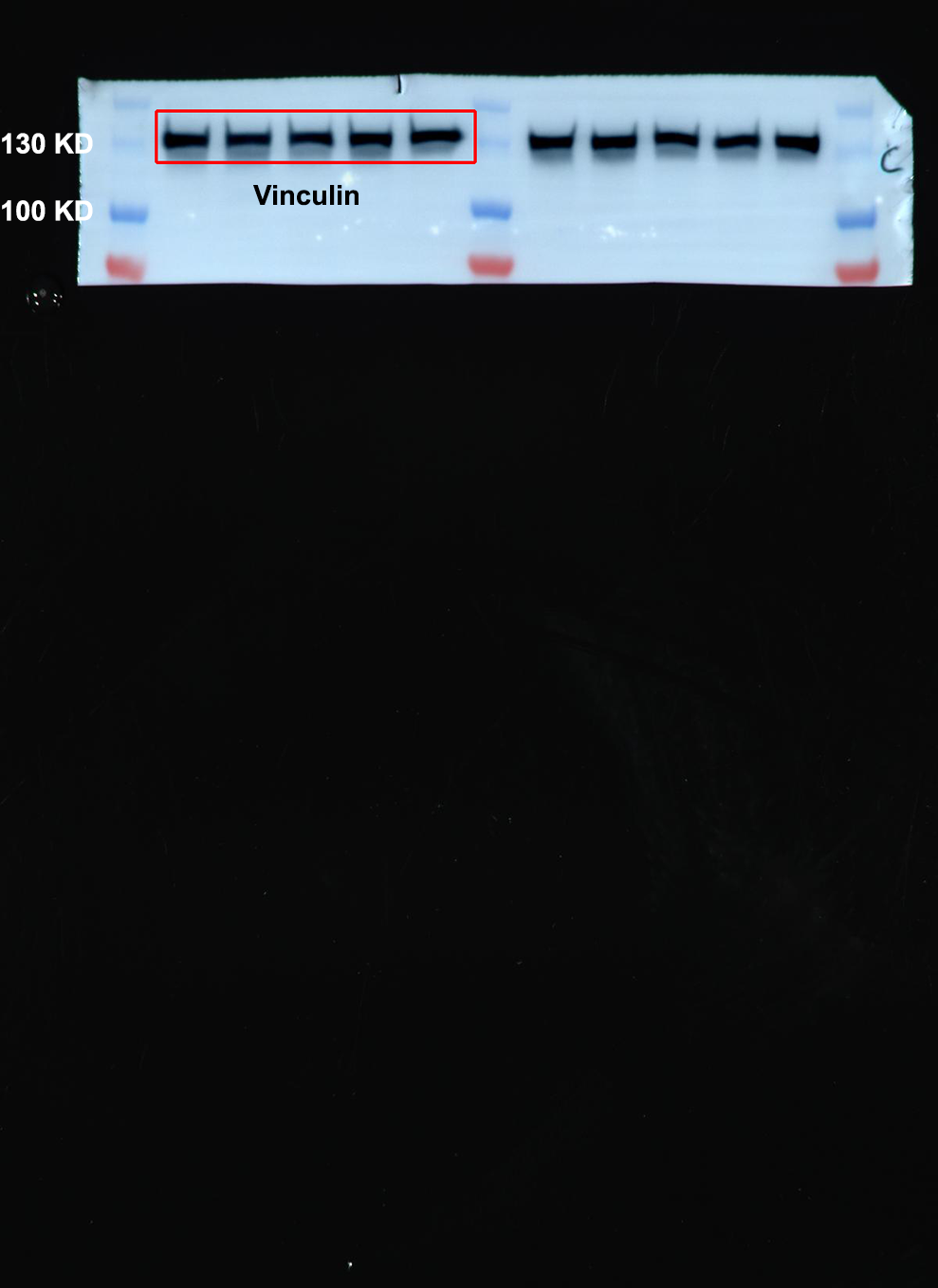

Supplement: Supplemental Information 3 [file peerj-12-17538-s003.zip › raw data-WB blots in Figure 6/drp/vinculin-c_134806_Ch_Chemi+Marker.tif]

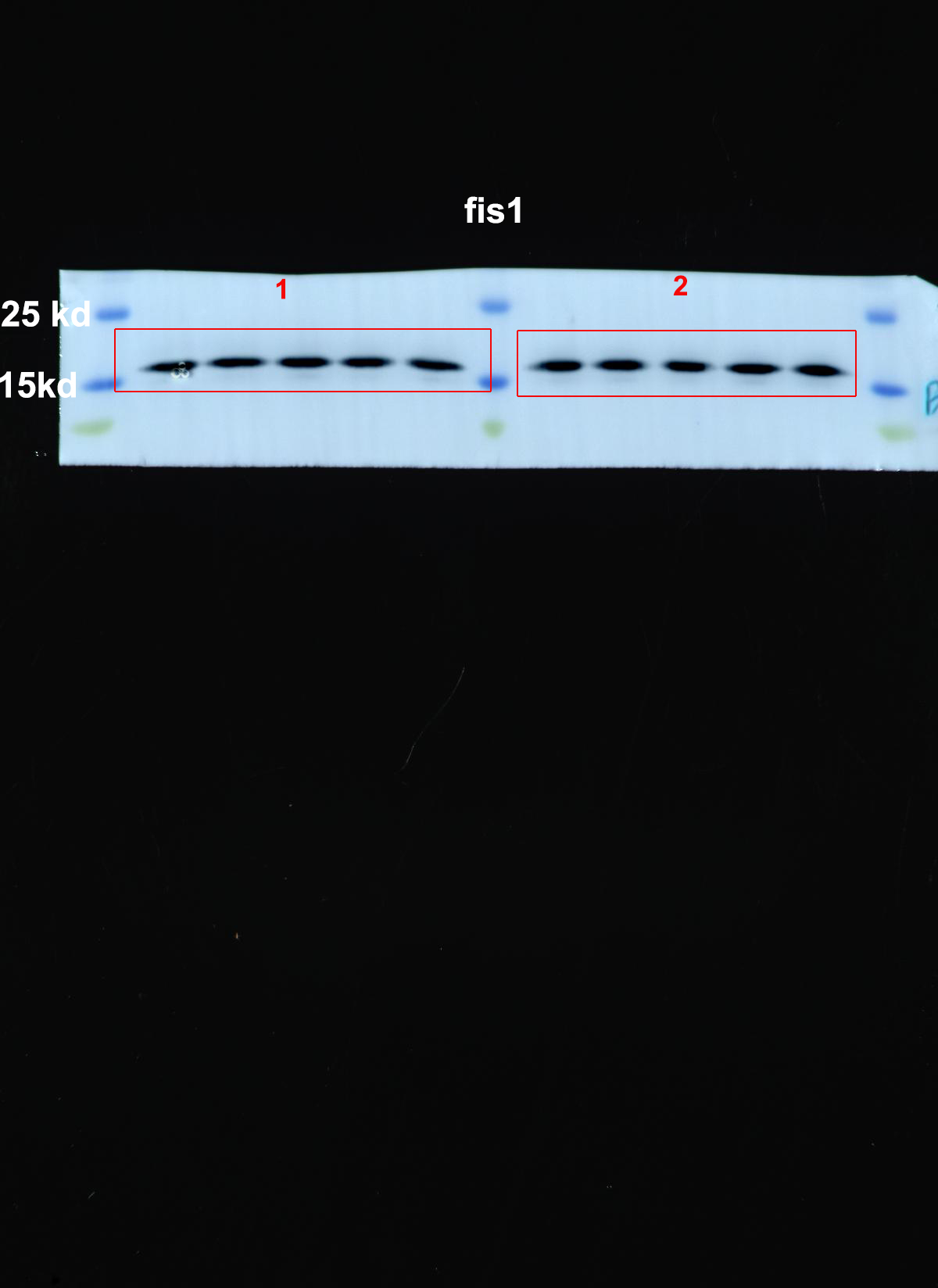

Supplement: Supplemental Information 3 [file peerj-12-17538-s003.zip › raw data-WB blots in Figure 6/fis/fis 20231104_181700_Ch_Chemi+Marker.jpg]

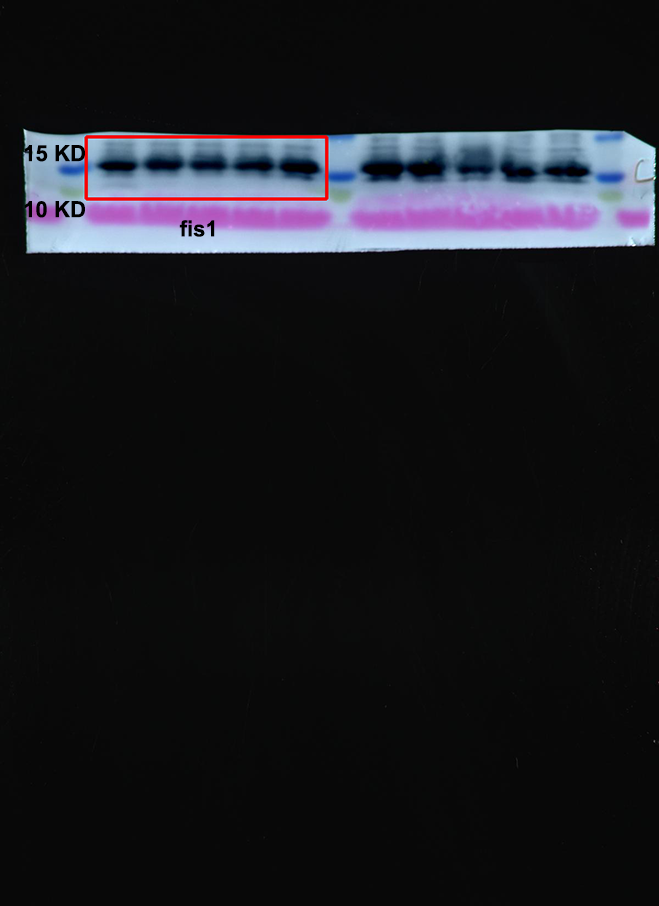

Supplement: Supplemental Information 3 [file peerj-12-17538-s003.zip › raw data-WB blots in Figure 6/fis/fis1_162232_Ch_Chemi+Marker.tif]

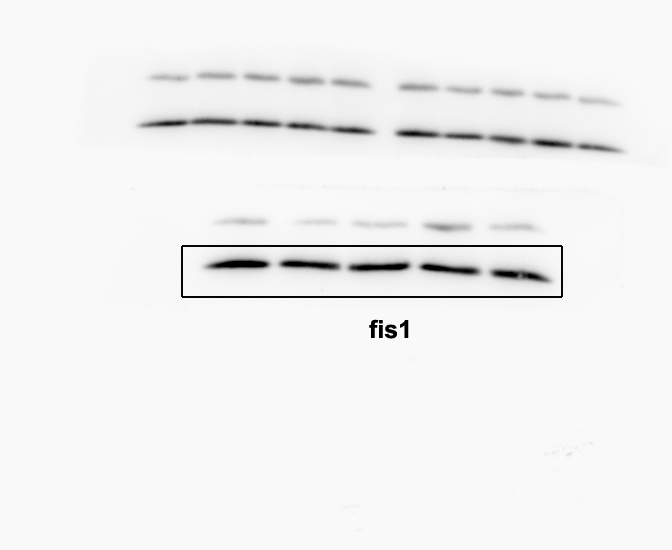

Supplement: Supplemental Information 3 [file peerj-12-17538-s003.zip › raw data-WB blots in Figure 6/fis/fis-2022.tif]

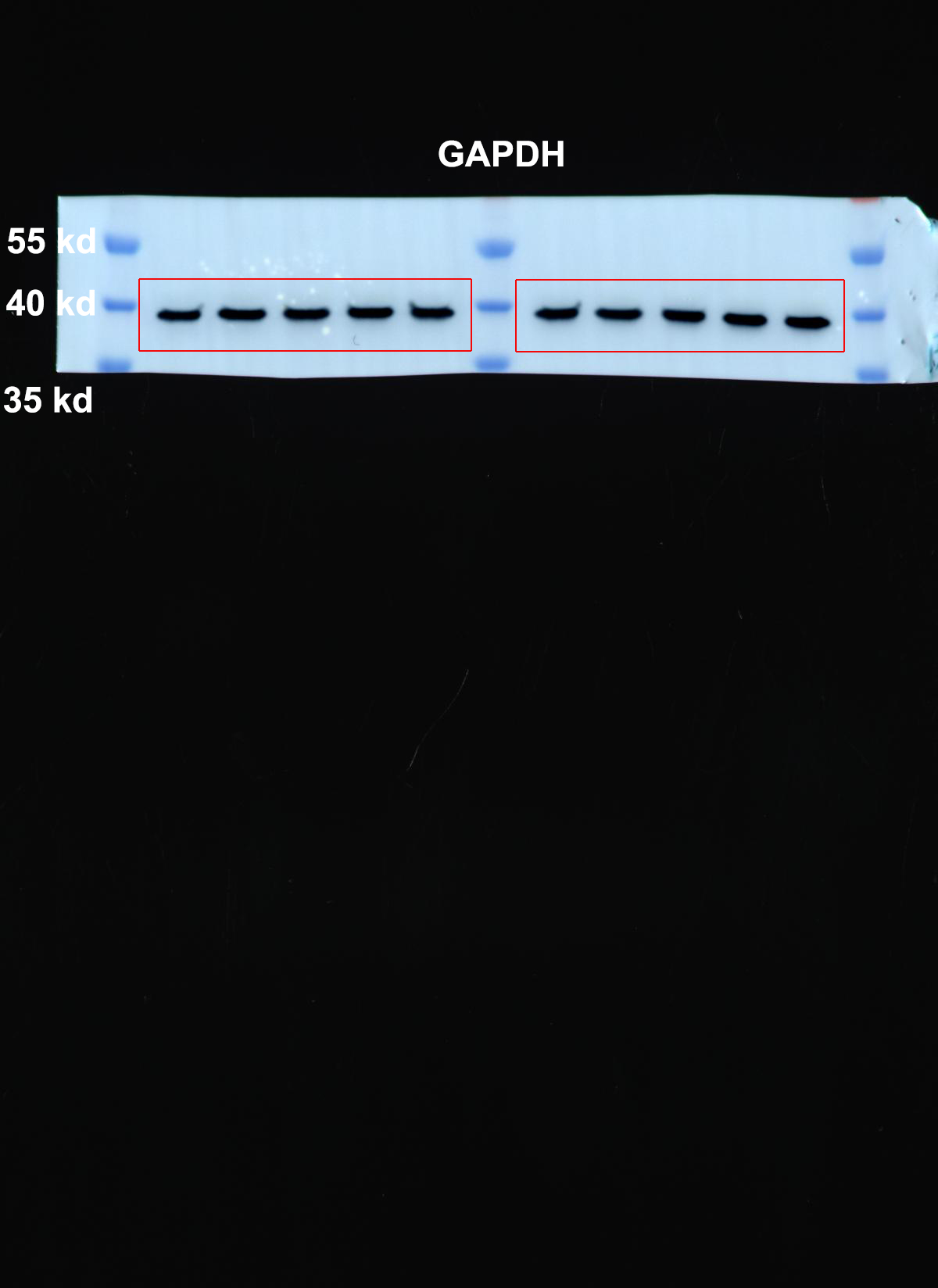

Supplement: Supplemental Information 3 [file peerj-12-17538-s003.zip › raw data-WB blots in Figure 6/fis/gapdh 20231106_154311_Ch_Chemi+Marker.jpg]

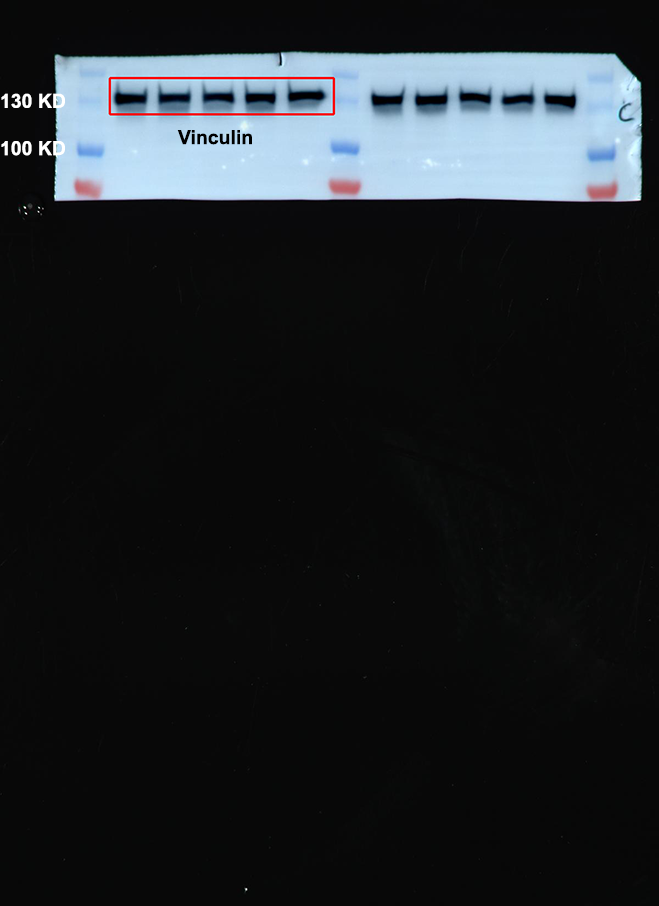

Supplement: Supplemental Information 3 [file peerj-12-17538-s003.zip › raw data-WB blots in Figure 6/fis/vinculin-c_134806_Ch_Chemi+Marker.tif]

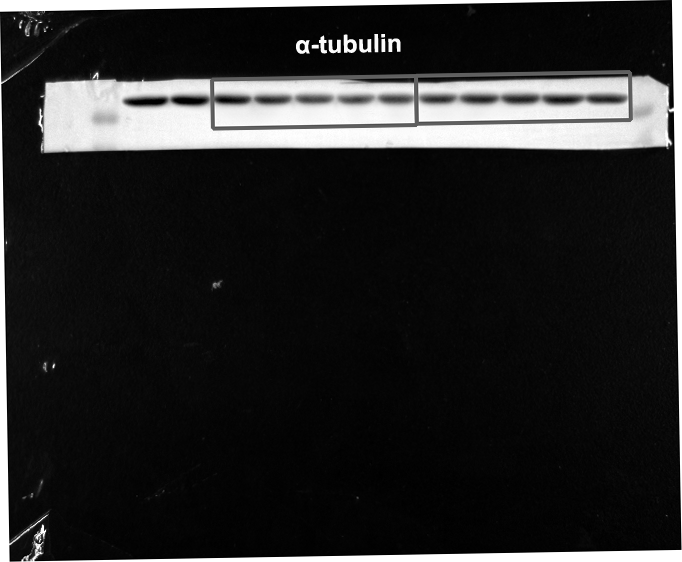

Supplement: Supplemental Information 3 [file peerj-12-17538-s003.zip › raw data-WB blots in Figure 6/LC3/GAPDH-A.tif]

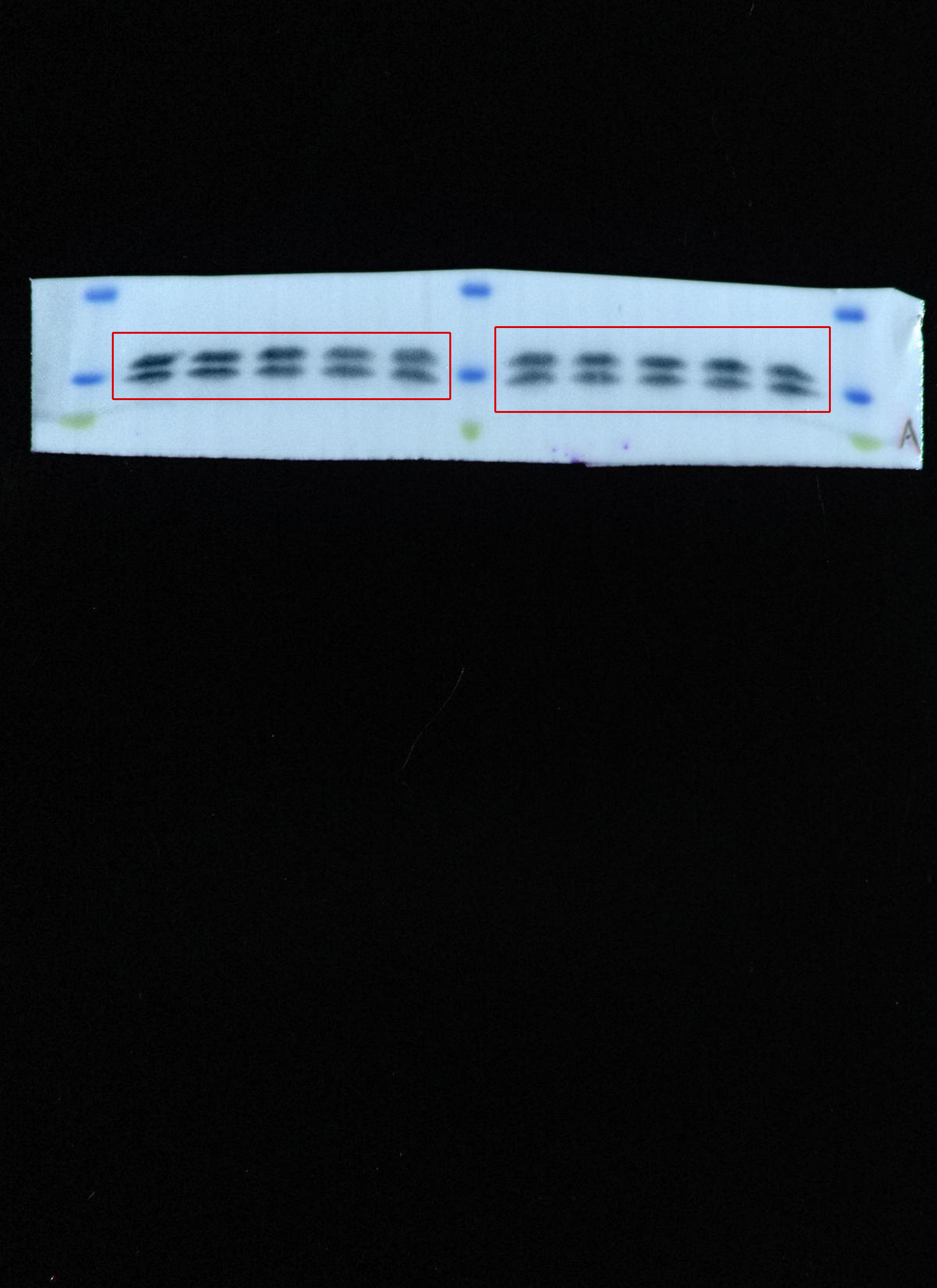

Supplement: Supplemental Information 3 [file peerj-12-17538-s003.zip › raw data-WB blots in Figure 6/LC3/lc3 20231104_181110_Ch_Chemi+Marker.jpg]

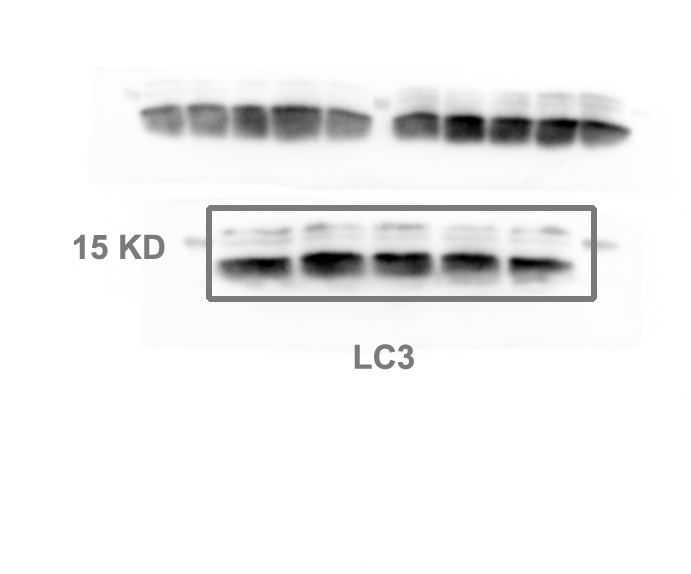

Supplement: Supplemental Information 3 [file peerj-12-17538-s003.zip › raw data-WB blots in Figure 6/LC3/lc3-below.tif]

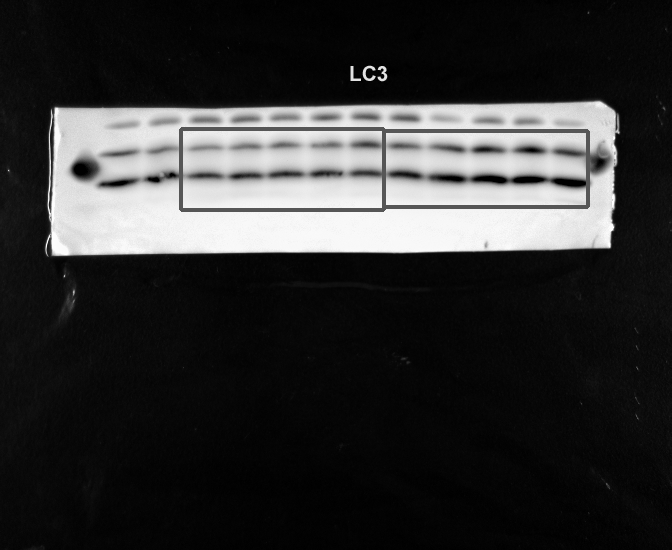

Supplement: Supplemental Information 3 [file peerj-12-17538-s003.zip › raw data-WB blots in Figure 6/LC3/LC3-C.tif]

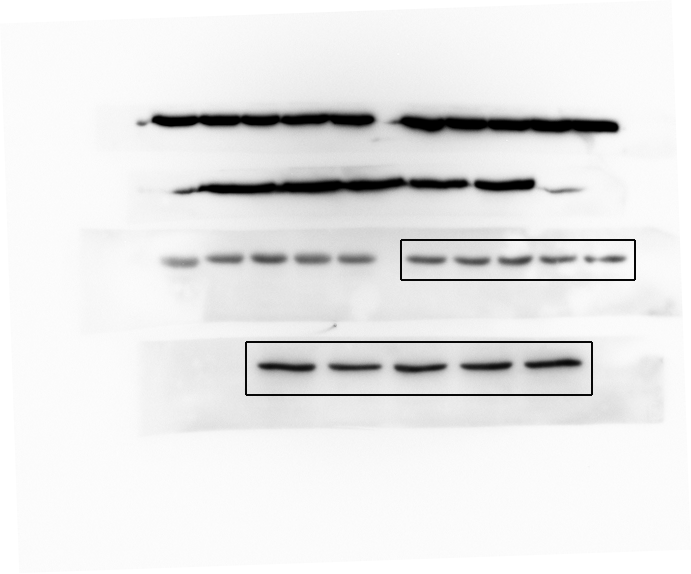

Supplement: Supplemental Information 3 [file peerj-12-17538-s003.zip › raw data-WB blots in Figure 6/LC3/tubulin-20220705.tif]

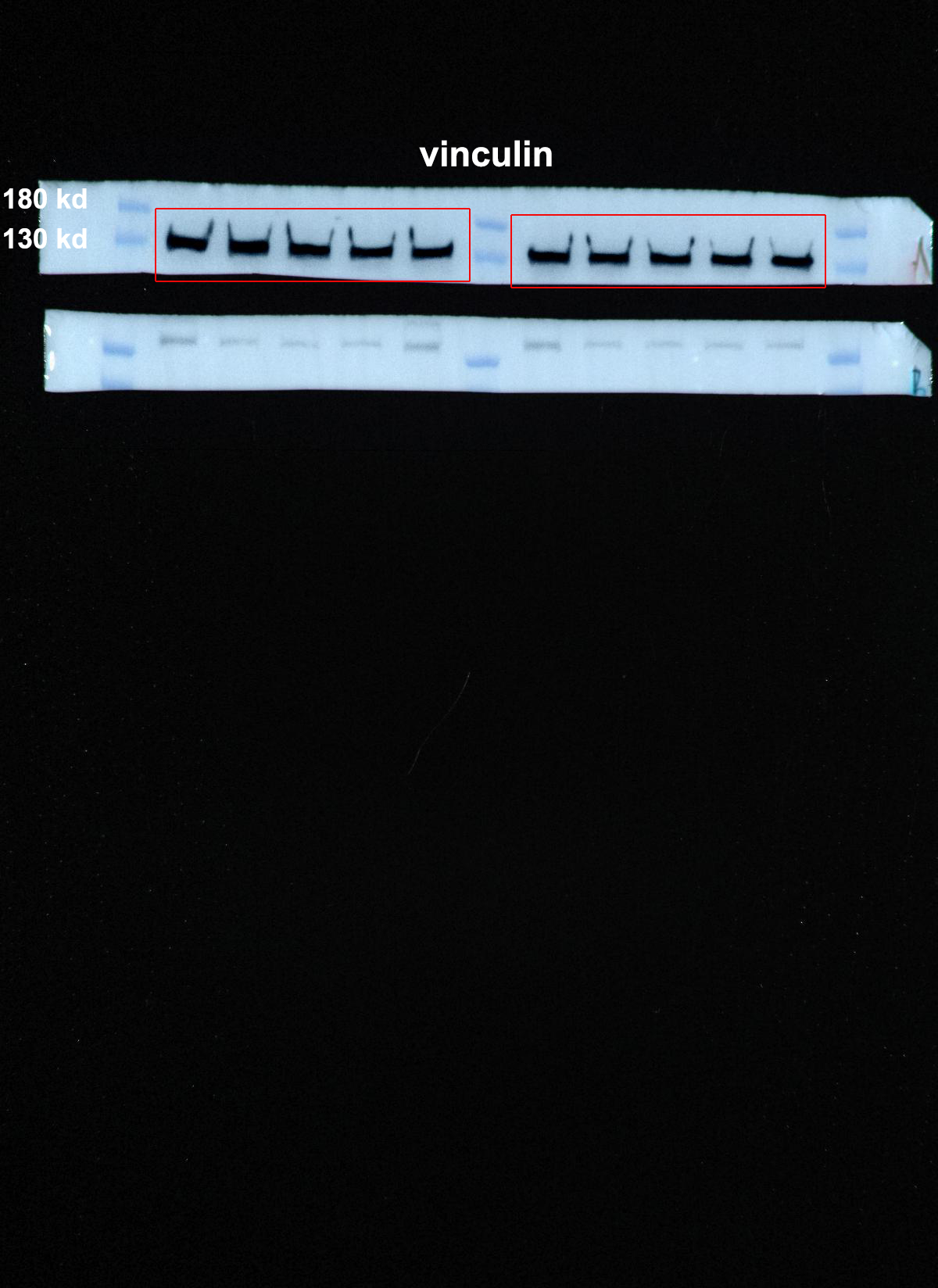

Supplement: Supplemental Information 3 [file peerj-12-17538-s003.zip › raw data-WB blots in Figure 6/LC3/vinculin 20231104_173949_Ch_Chemi+Marker.jpg]

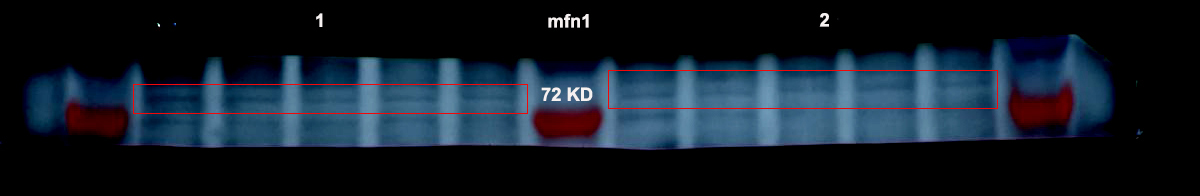

Supplement: Supplemental Information 3 [file peerj-12-17538-s003.zip › raw data-WB blots in Figure 6/mfn/mfn1 20230905_151923_Ch_Chemi+Marker.jpg]

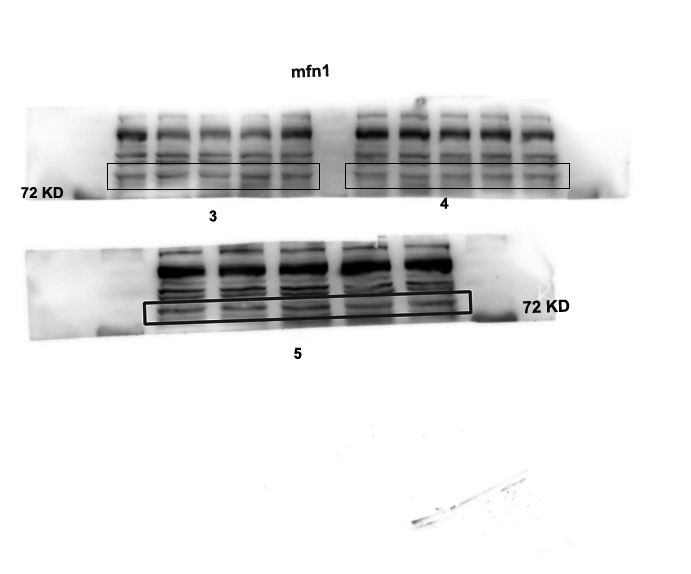

Supplement: Supplemental Information 3 [file peerj-12-17538-s003.zip › raw data-WB blots in Figure 6/mfn/mfn1-2022.tif]

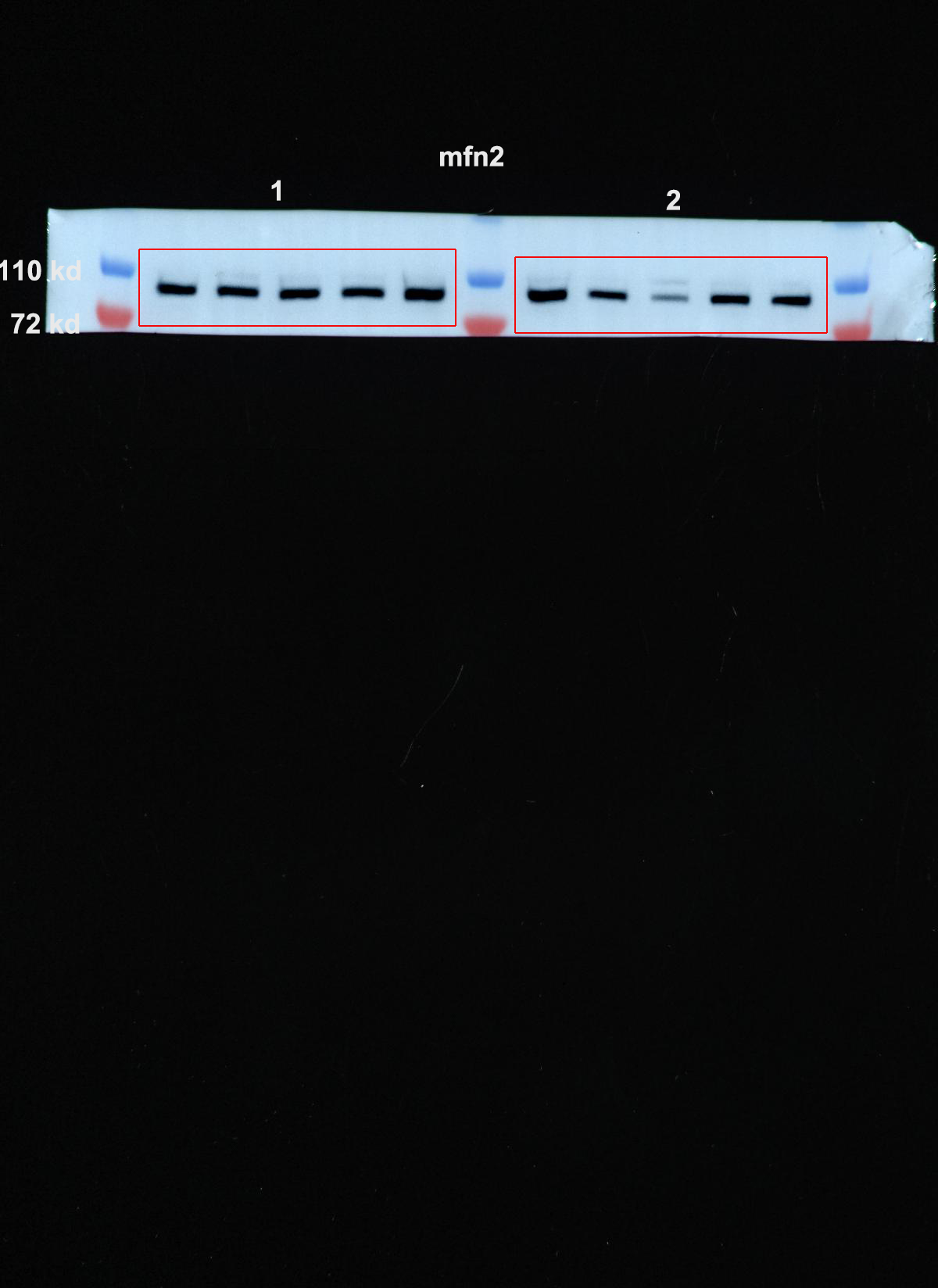

Supplement: Supplemental Information 3 [file peerj-12-17538-s003.zip › raw data-WB blots in Figure 6/mfn/mfn2 20231106_153252_Ch_Chemi+Marker.jpg]

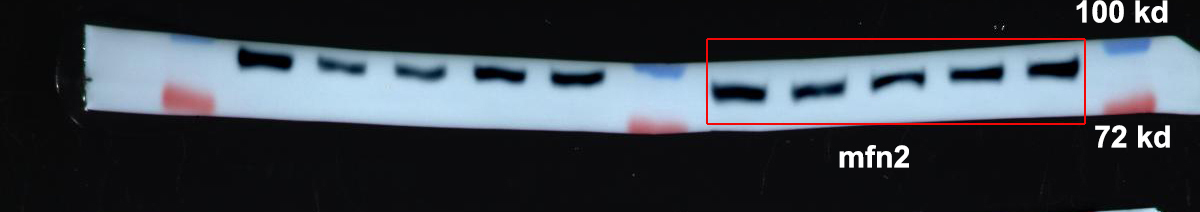

Supplement: Supplemental Information 3 [file peerj-12-17538-s003.zip › raw data-WB blots in Figure 6/mfn/mfn2_150857_Ch_Chemi+Marker.jpg]

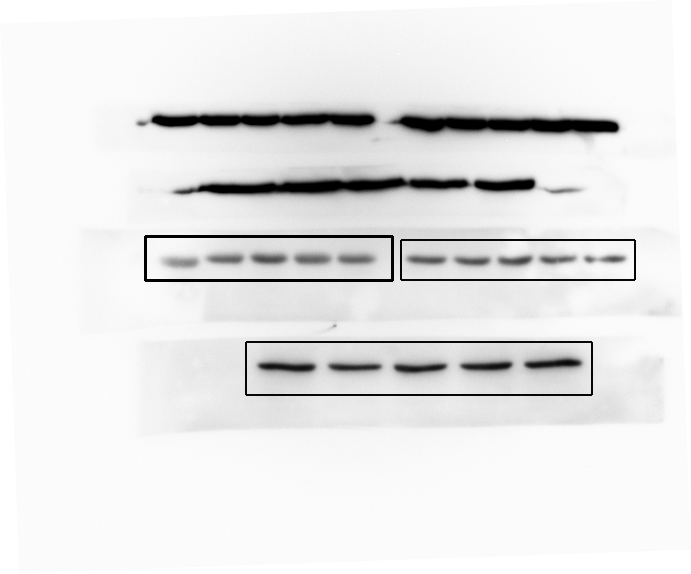

Supplement: Supplemental Information 3 [file peerj-12-17538-s003.zip › raw data-WB blots in Figure 6/mfn/tubulin-20220705.tif]

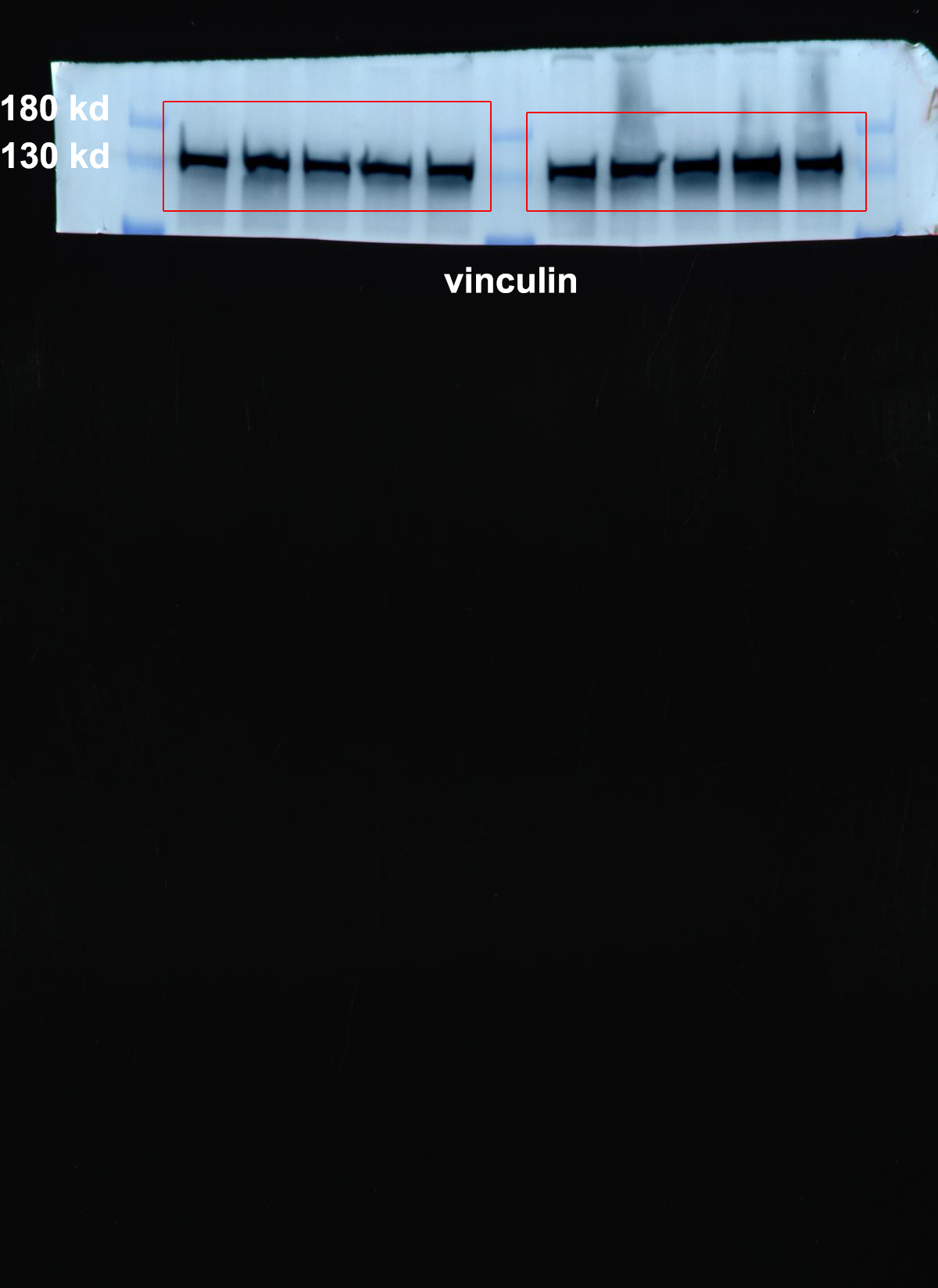

Supplement: Supplemental Information 3 [file peerj-12-17538-s003.zip › raw data-WB blots in Figure 6/mfn/vinculin 20230906_144940_Ch_Chemi+Marker.jpg]

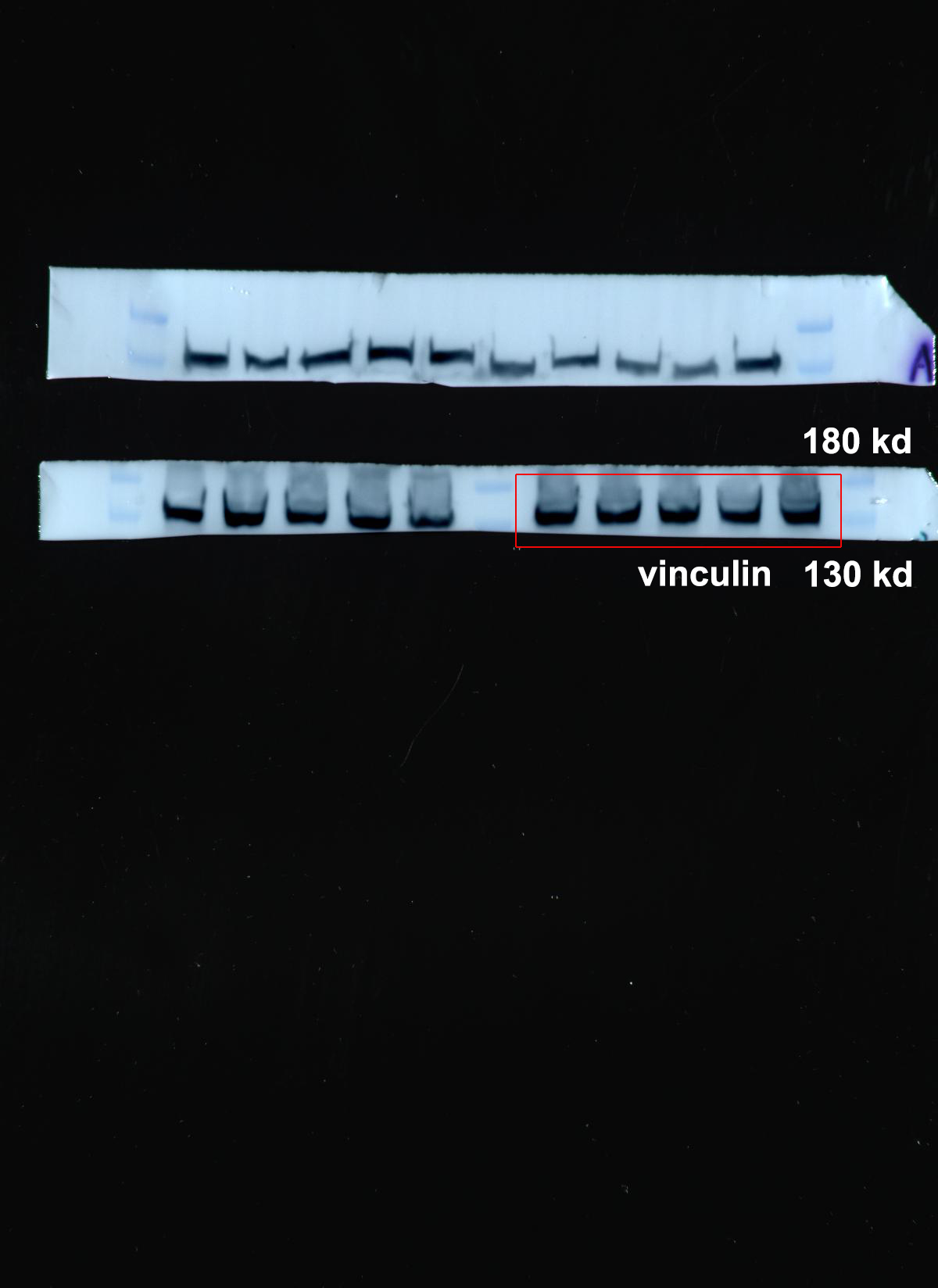

Supplement: Supplemental Information 3 [file peerj-12-17538-s003.zip › raw data-WB blots in Figure 6/mfn/vinculin 20231128_135807_Ch_Chemi+Marker.jpg]

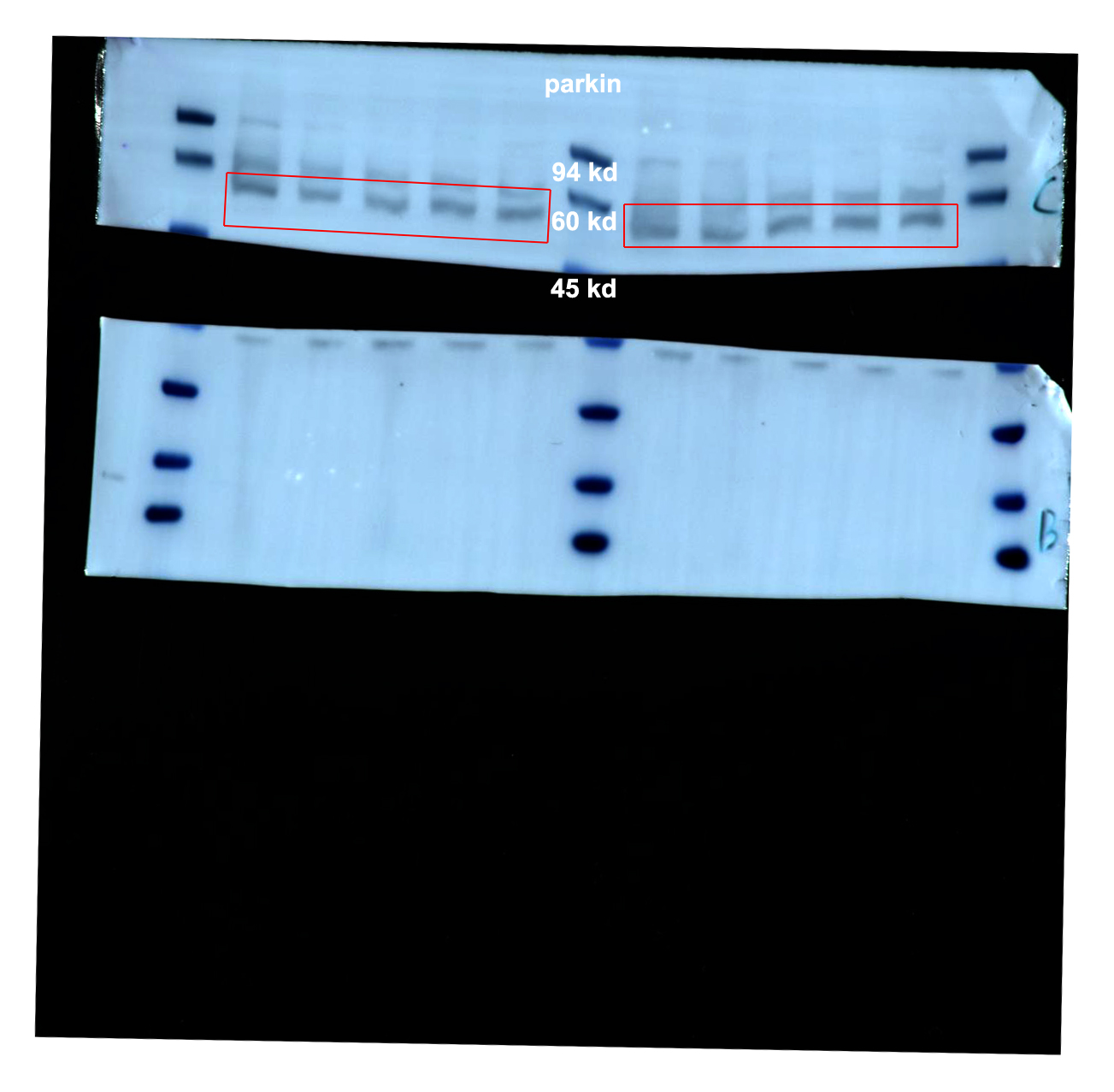

Supplement: Supplemental Information 3 [file peerj-12-17538-s003.zip › raw data-WB blots in Figure 6/parkin/parkin_182730_Ch_Chemi+Marker (1).jpg]

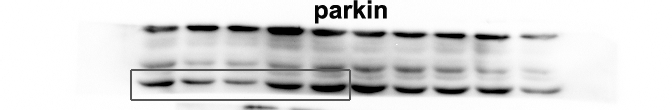

Supplement: Supplemental Information 3 [file peerj-12-17538-s003.zip › raw data-WB blots in Figure 6/parkin/parkin-20220703.tif]

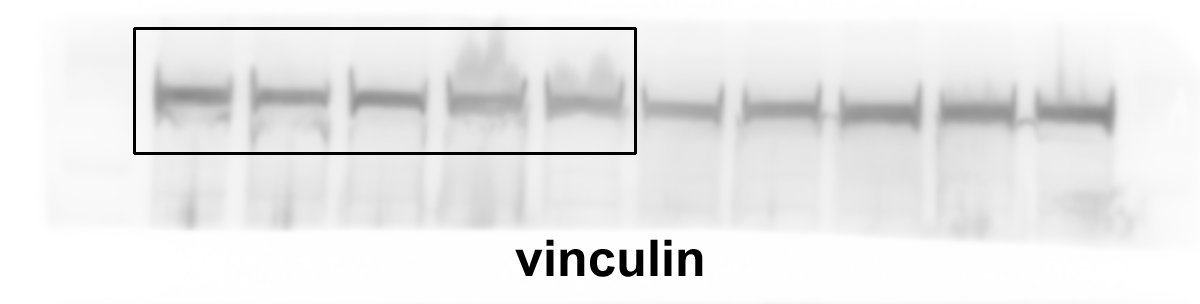

Supplement: Supplemental Information 3 [file peerj-12-17538-s003.zip › raw data-WB blots in Figure 6/parkin/vinculin 20220703_124918_Ch_Chemi.tif]

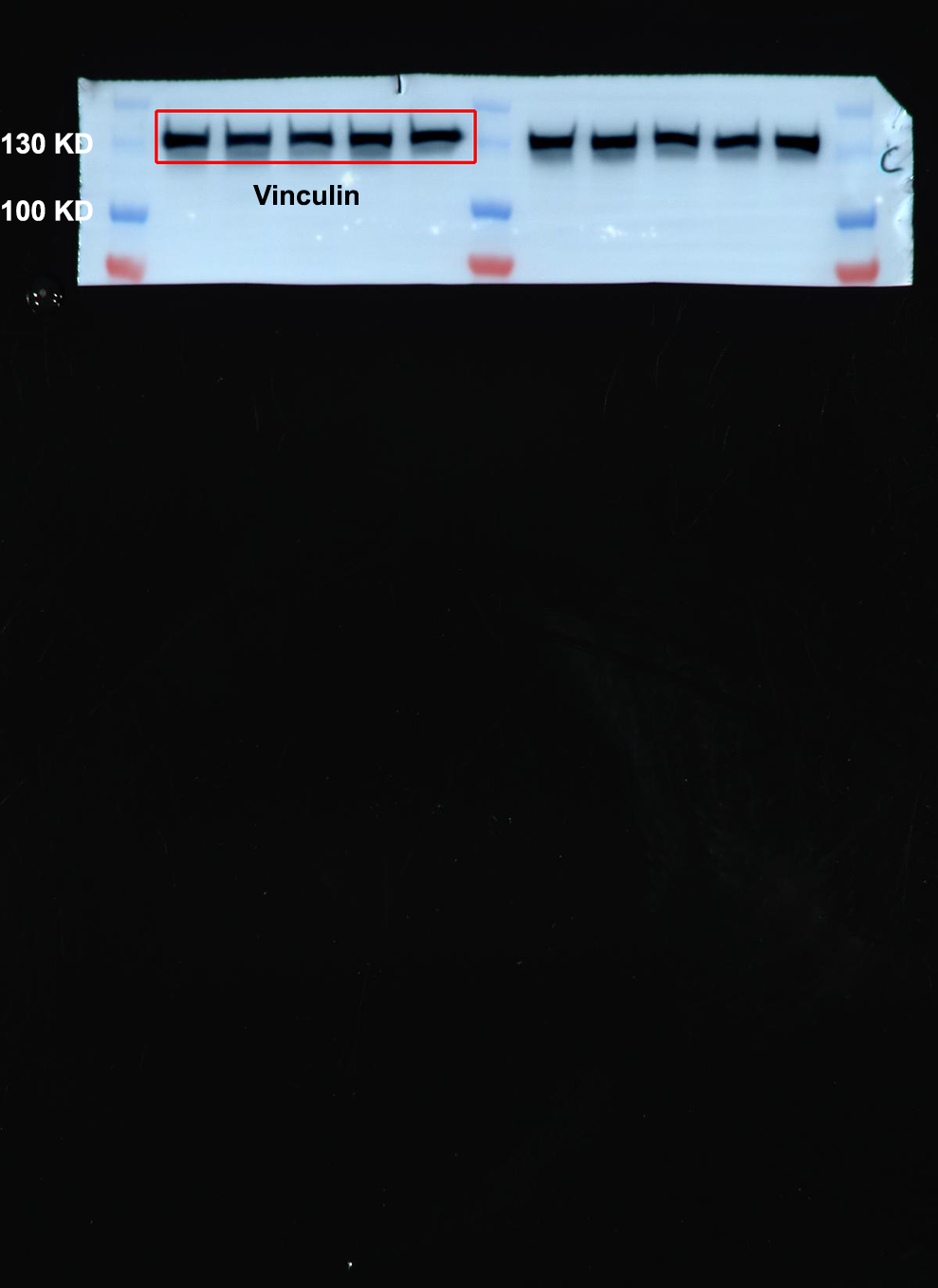

Supplement: Supplemental Information 3 [file peerj-12-17538-s003.zip › raw data-WB blots in Figure 6/parkin/vinculin-c_134806_Ch_Chemi+Marker.tif]

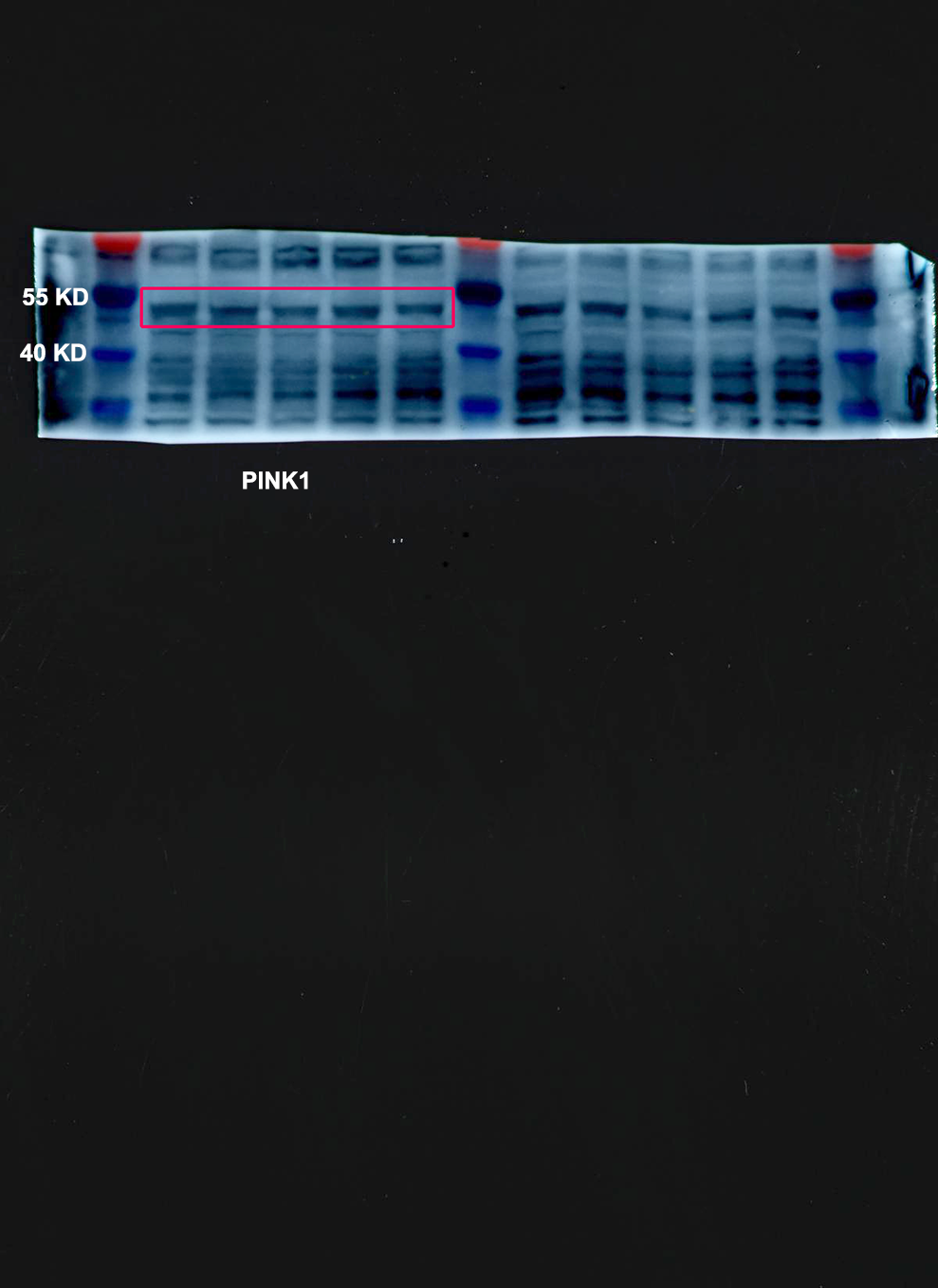

Supplement: Supplemental Information 3 [file peerj-12-17538-s003.zip › raw data-WB blots in Figure 6/pink/pink_163626_Ch_Chemi+Marker.tif]

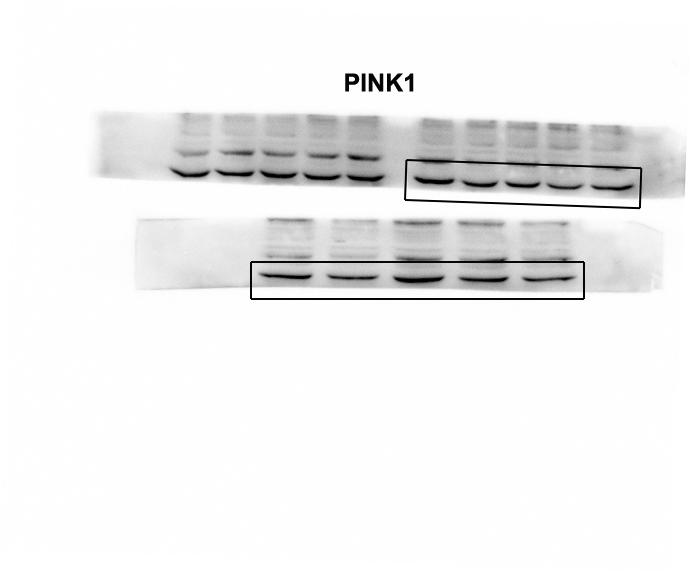

Supplement: Supplemental Information 3 [file peerj-12-17538-s003.zip › raw data-WB blots in Figure 6/pink/pink1-20220705.tif]

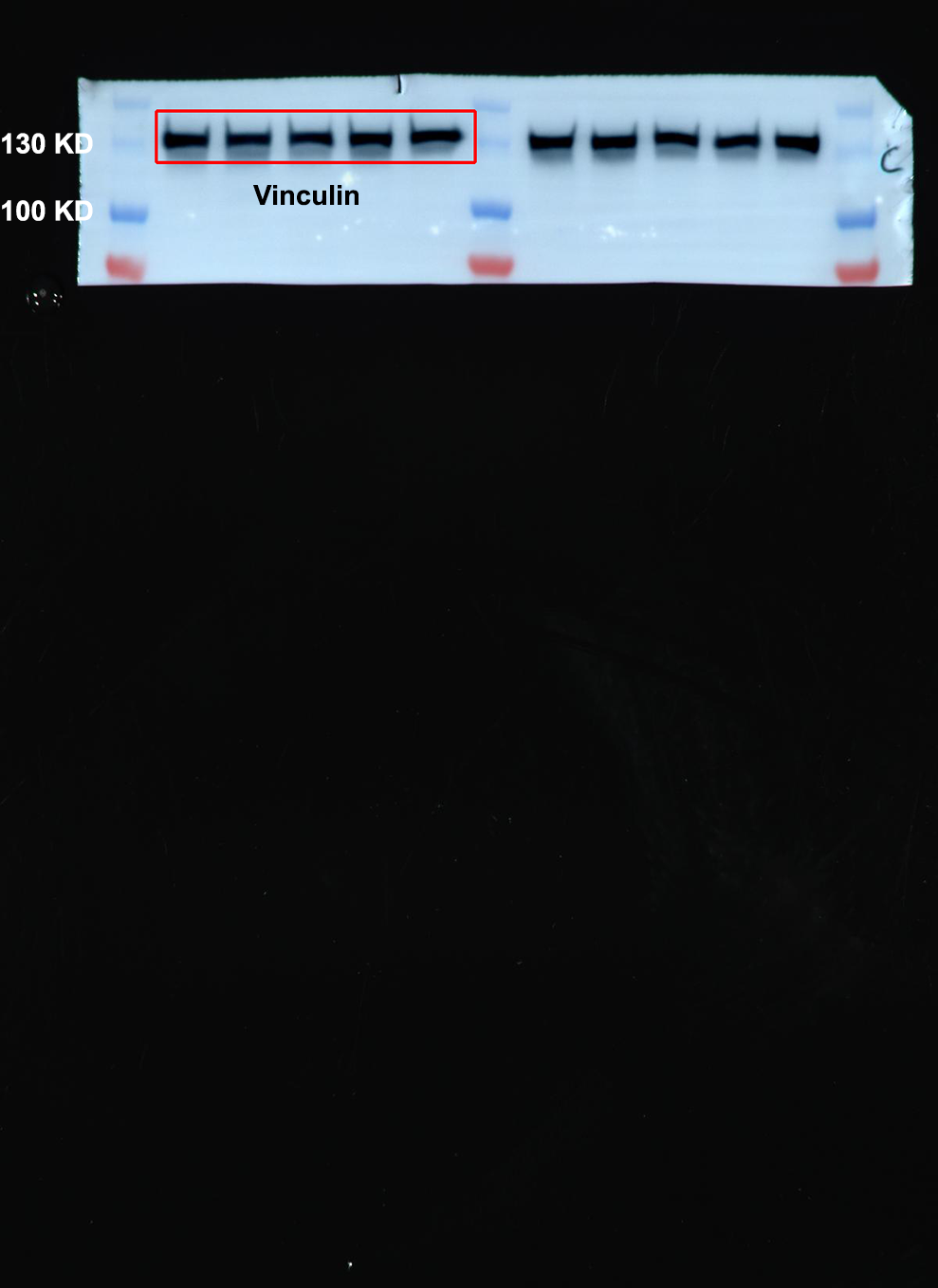

Supplement: Supplemental Information 3 [file peerj-12-17538-s003.zip › raw data-WB blots in Figure 6/pink/vinculin-c_134806_Ch_Chemi+Marker.tif]

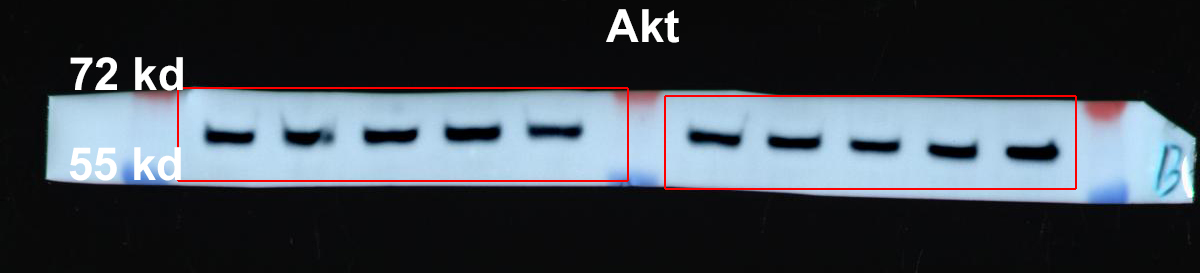

Supplement: Supplemental Information 4 [file peerj-12-17538-s004.zip › raw data-WB blots in Figure 7/akt/akt 20231128_144552_Ch_Chemi+Marker.jpg]

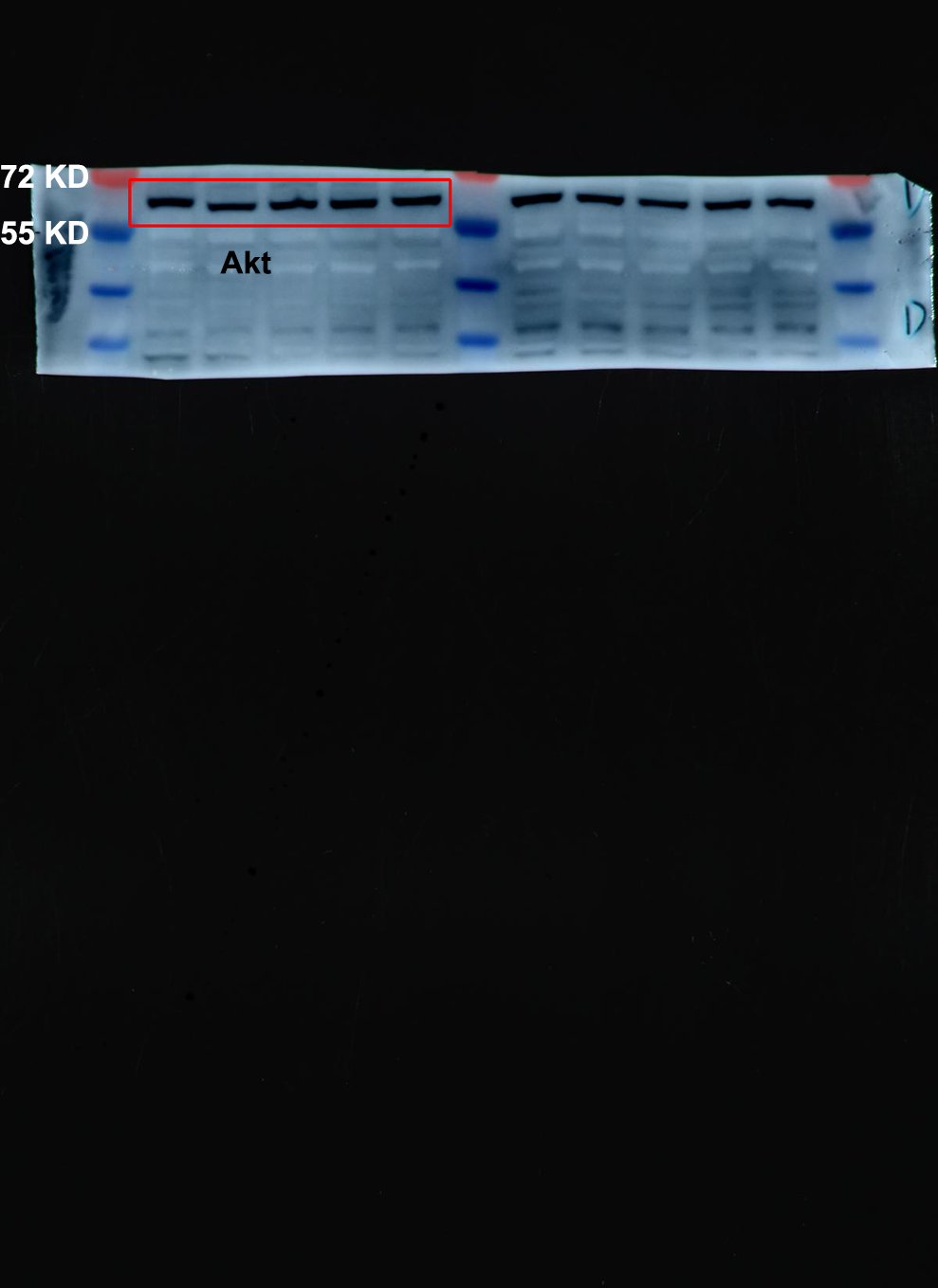

Supplement: Supplemental Information 4 [file peerj-12-17538-s004.zip › raw data-WB blots in Figure 7/akt/akt_144356_Ch_Chemi-marker.tif]

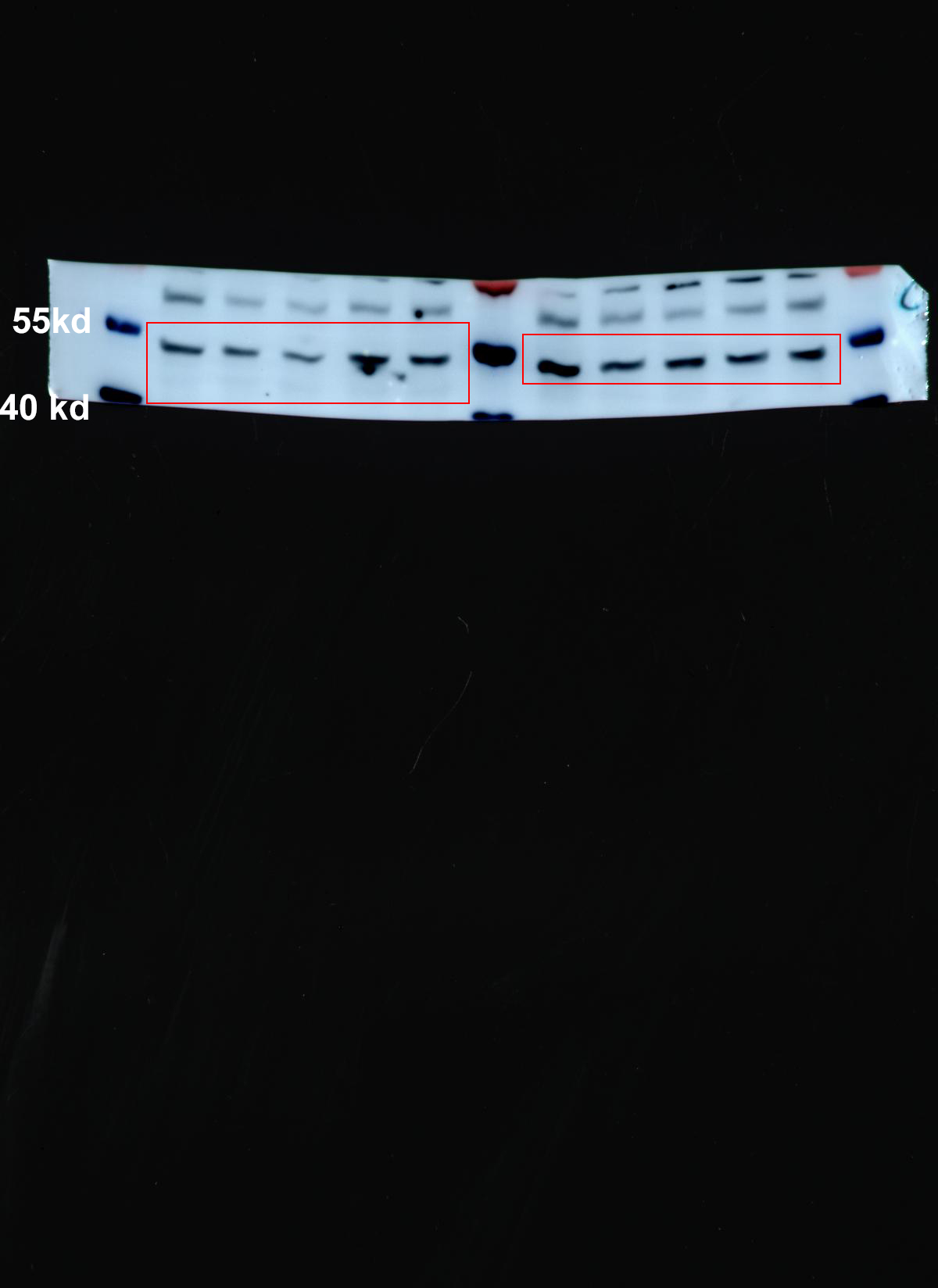

Supplement: Supplemental Information 4 [file peerj-12-17538-s004.zip › raw data-WB blots in Figure 7/akt/p-akt _143339_Ch_Chemi+Marker.jpg]

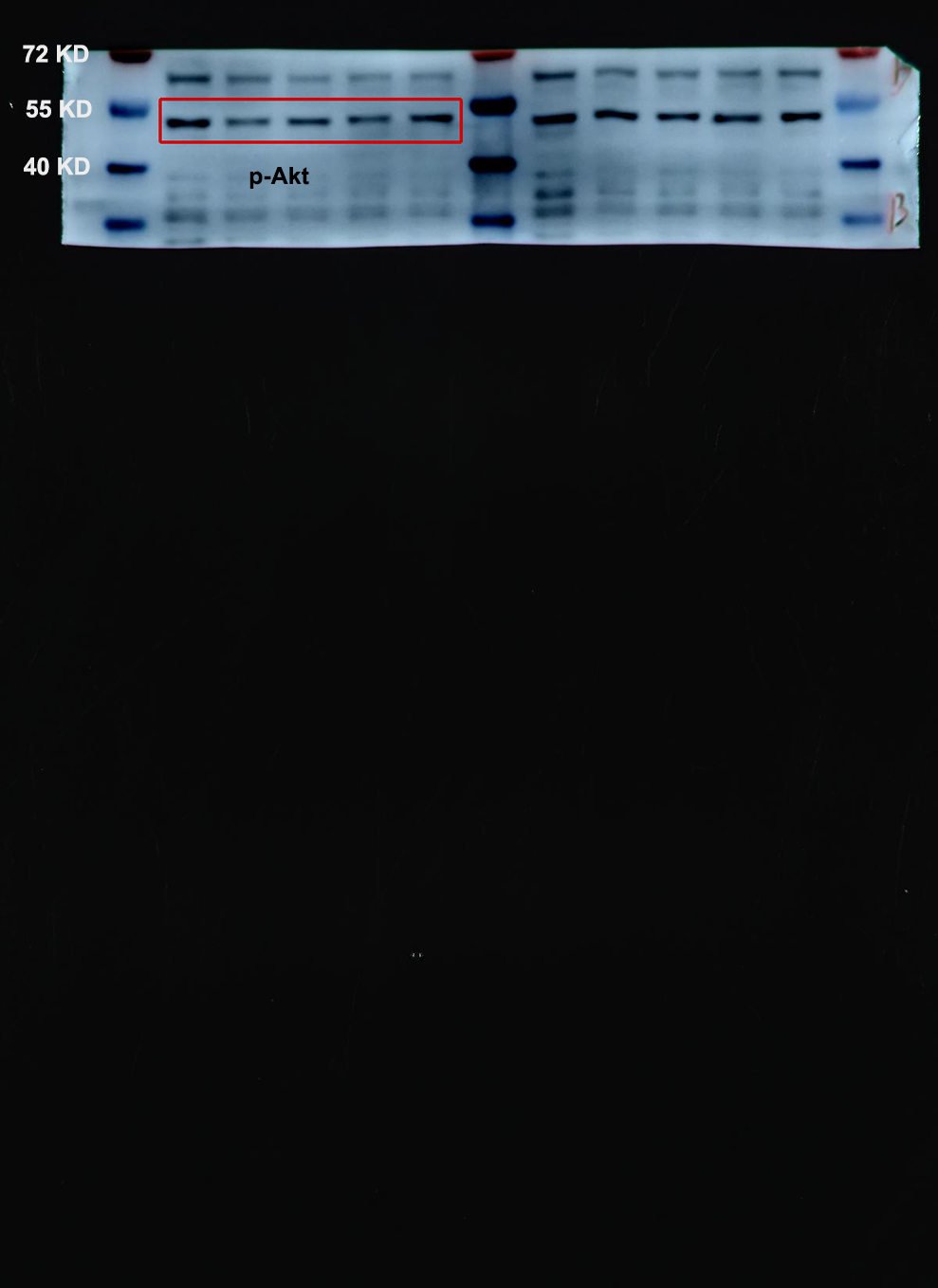

Supplement: Supplemental Information 4 [file peerj-12-17538-s004.zip › raw data-WB blots in Figure 7/akt/p-akt_143938_Ch_Chemi+Marker.tif]

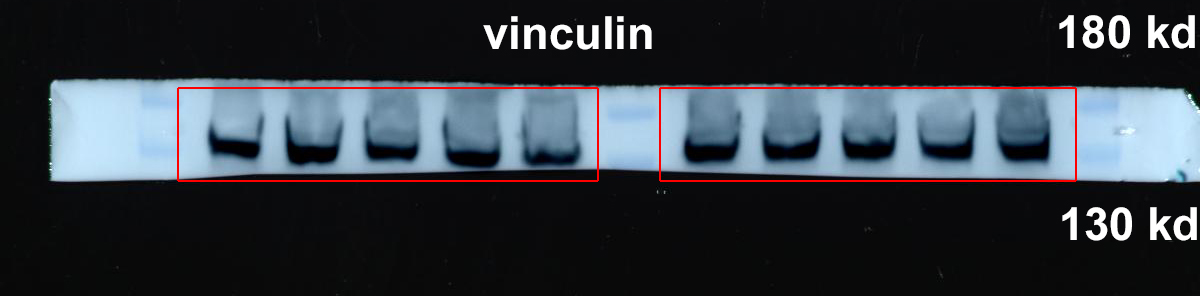

Supplement: Supplemental Information 4 [file peerj-12-17538-s004.zip › raw data-WB blots in Figure 7/akt/vinculin 135807_Ch_Chemi+Marker.jpg]

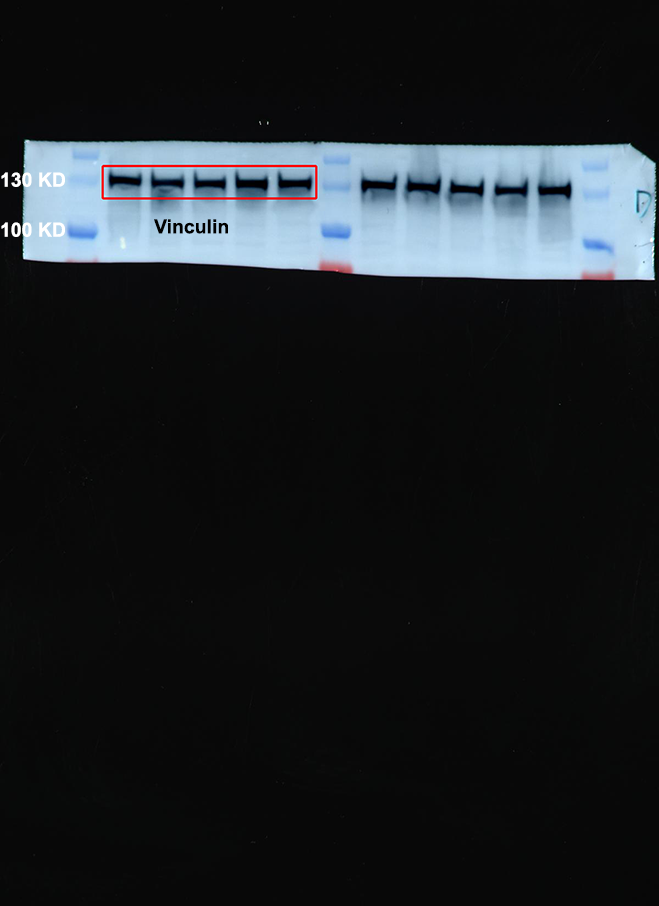

Supplement: Supplemental Information 4 [file peerj-12-17538-s004.zip › raw data-WB blots in Figure 7/akt/vinculin-d_145955_Ch_Chemi+Marker.tif]

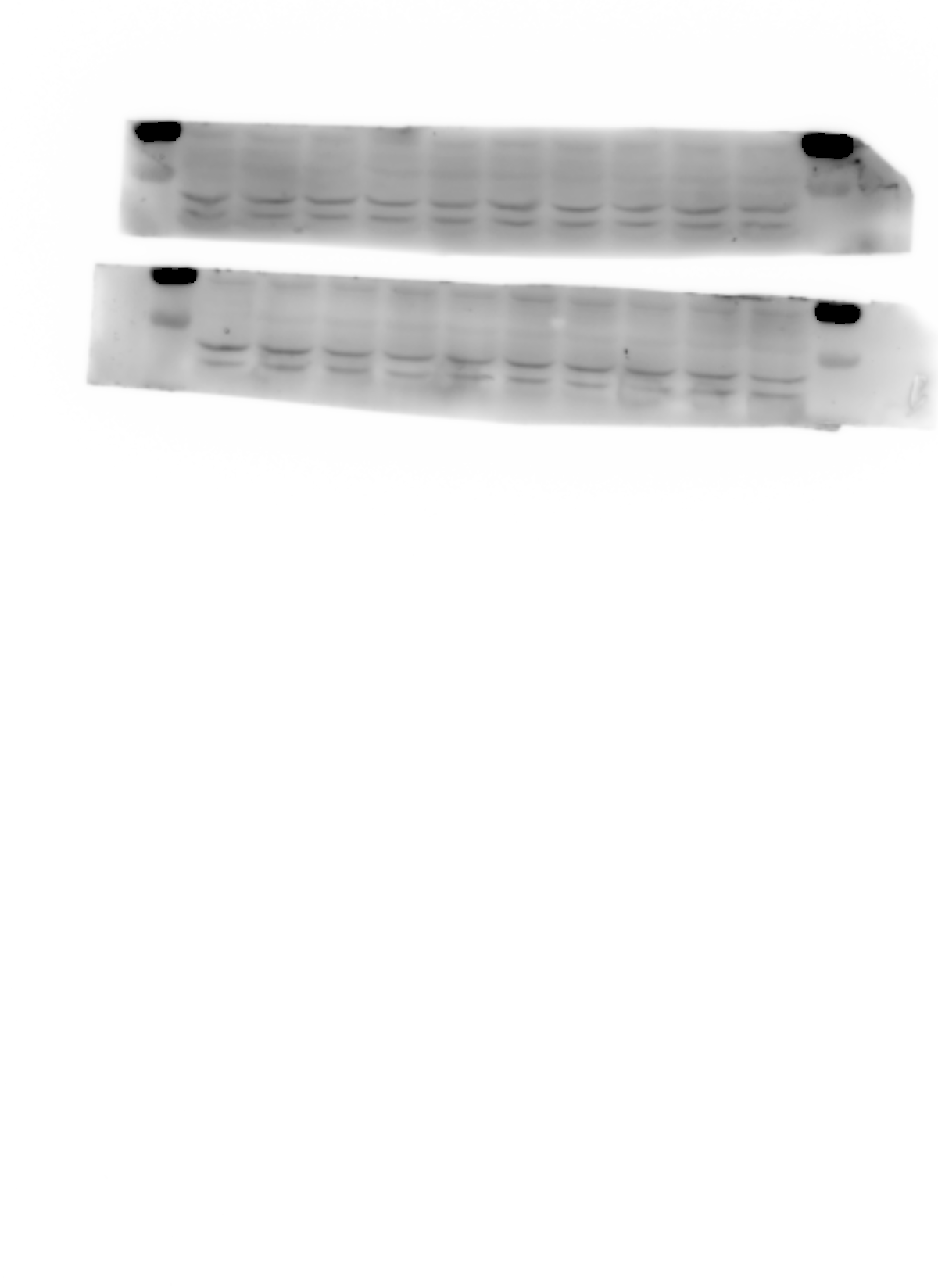

Supplement: Supplemental Information 4 [file peerj-12-17538-s004.zip › raw data-WB blots in Figure 7/gsk3a┬/gsk 20230713_141357_Ch_Chemi.tif]

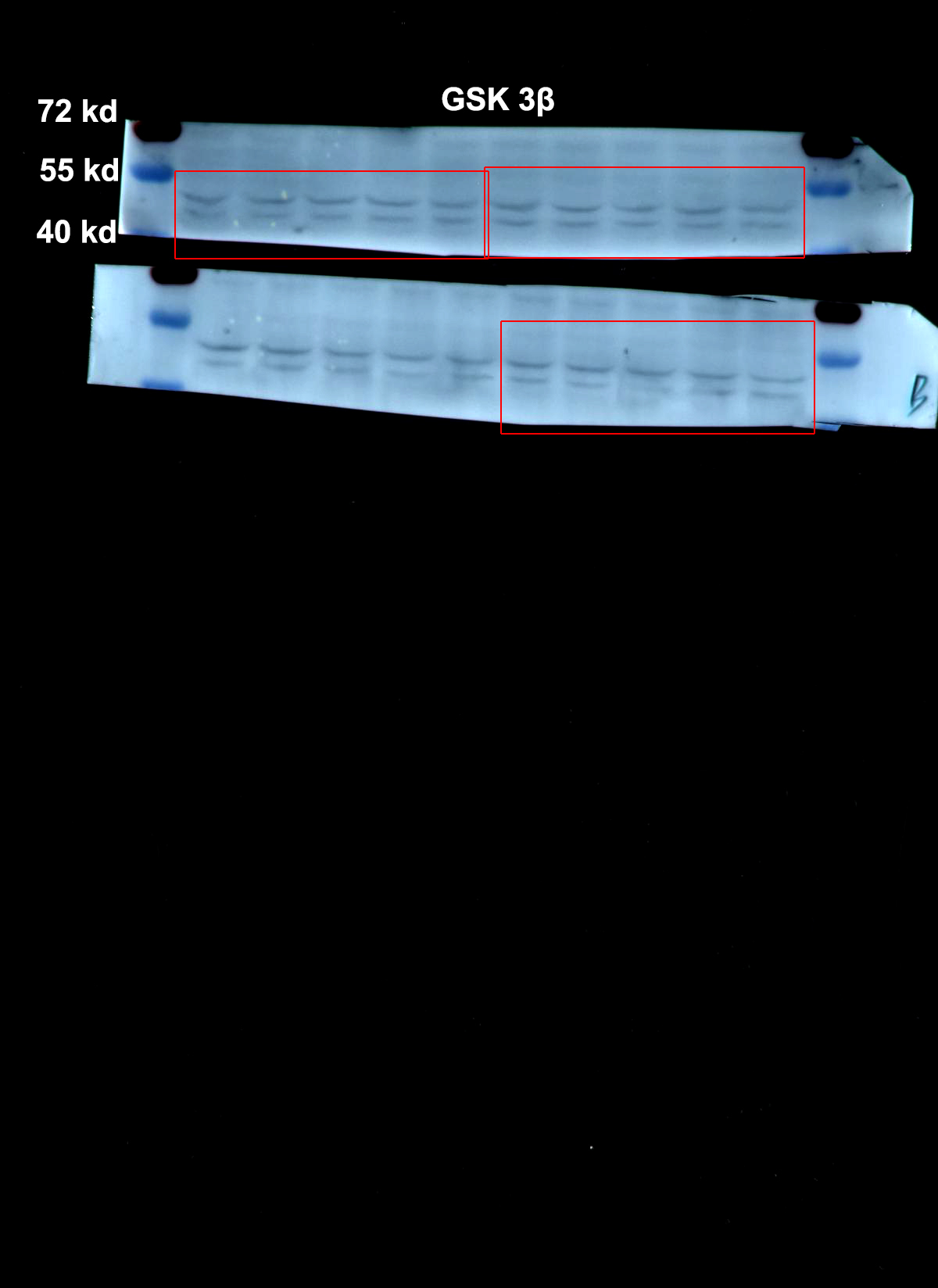

Supplement: Supplemental Information 4 [file peerj-12-17538-s004.zip › raw data-WB blots in Figure 7/gsk3a┬/gsk 20230713_141552_Ch_Chemi+Marker.jpg]

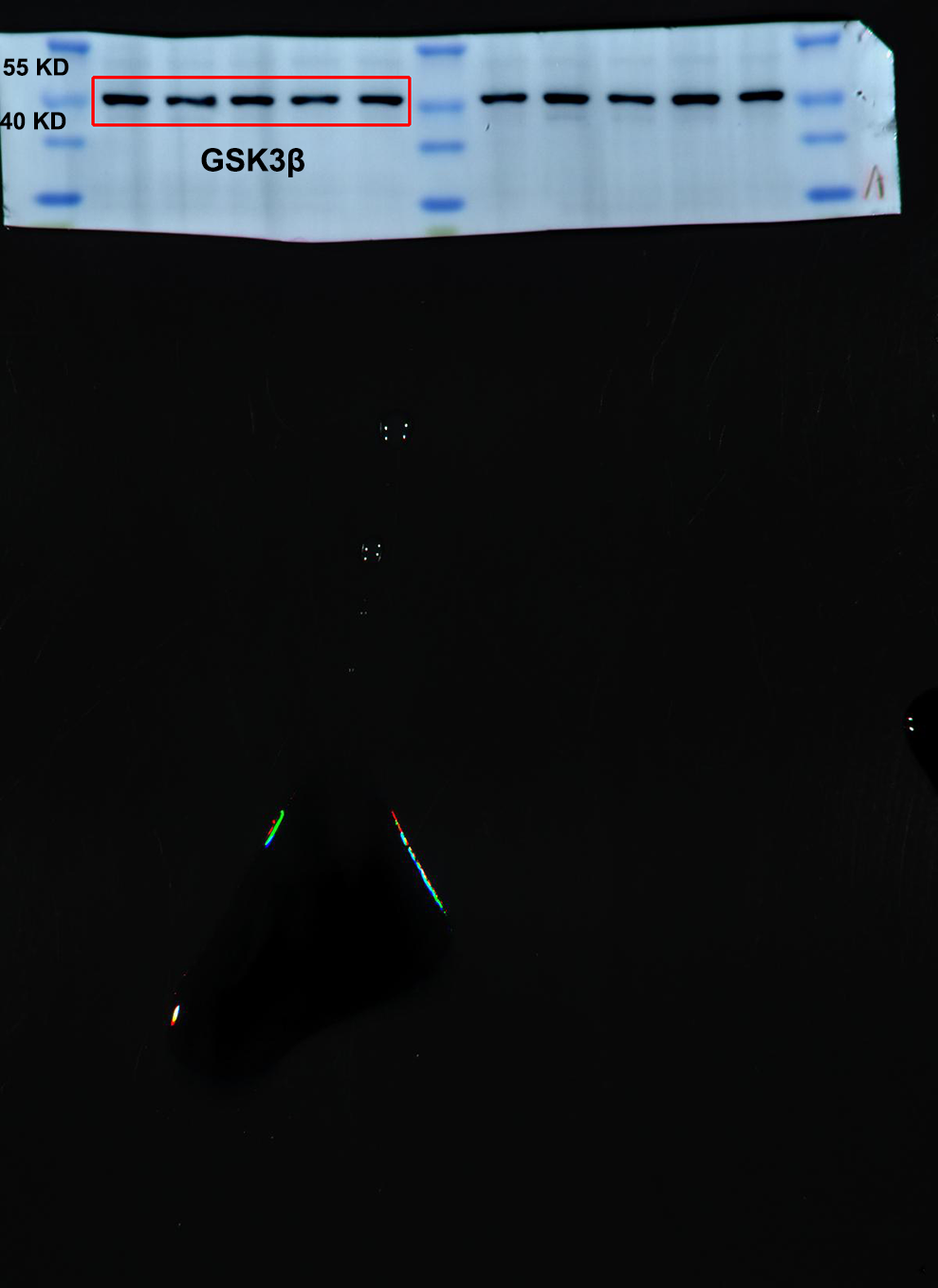

Supplement: Supplemental Information 4 [file peerj-12-17538-s004.zip › raw data-WB blots in Figure 7/gsk3a┬/gsk_152109_Ch_Chemi+Marker.tif]

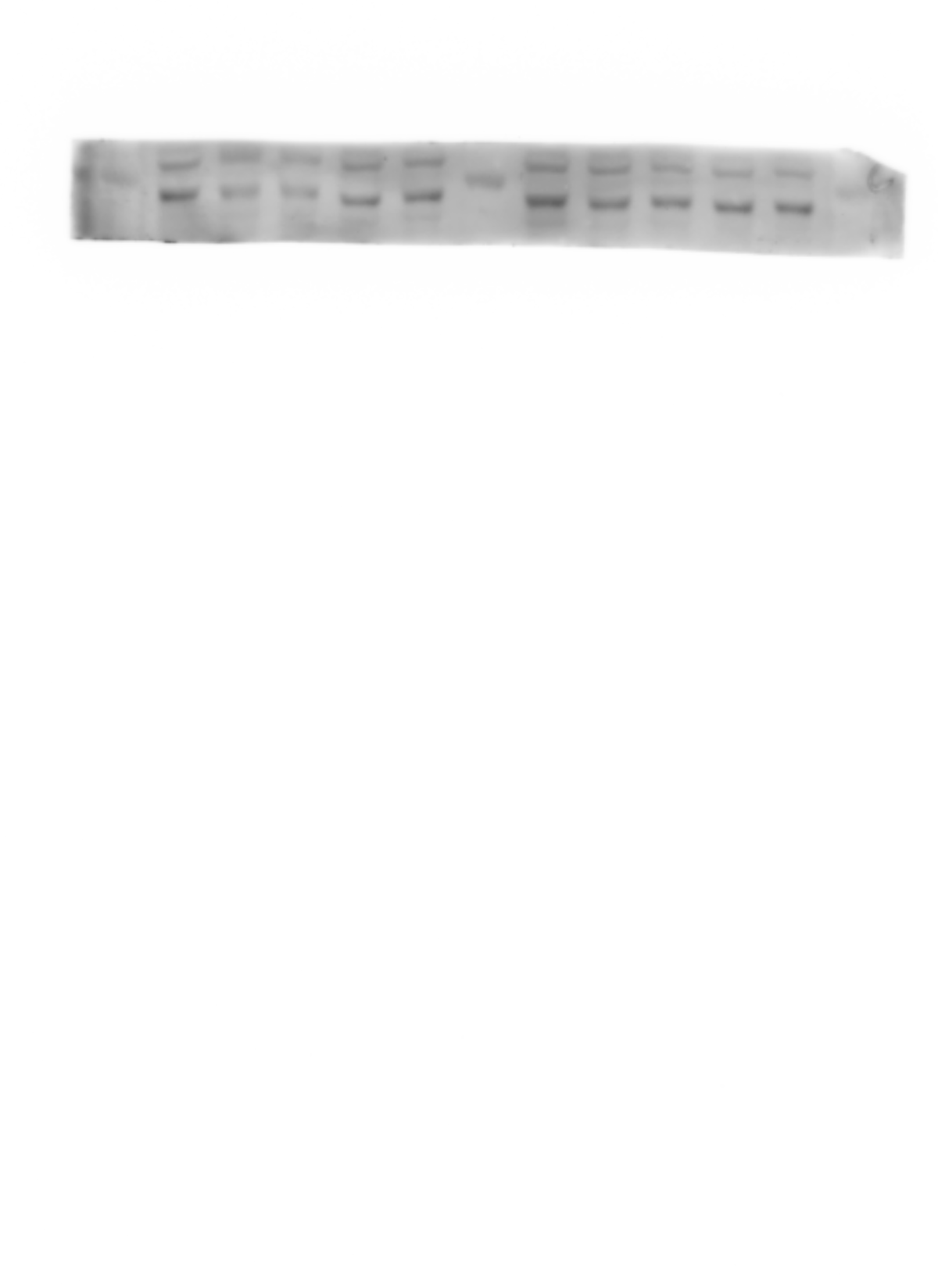

Supplement: Supplemental Information 4 [file peerj-12-17538-s004.zip › raw data-WB blots in Figure 7/gsk3a┬/p-gsk_145032_Ch_Chemi.tif]

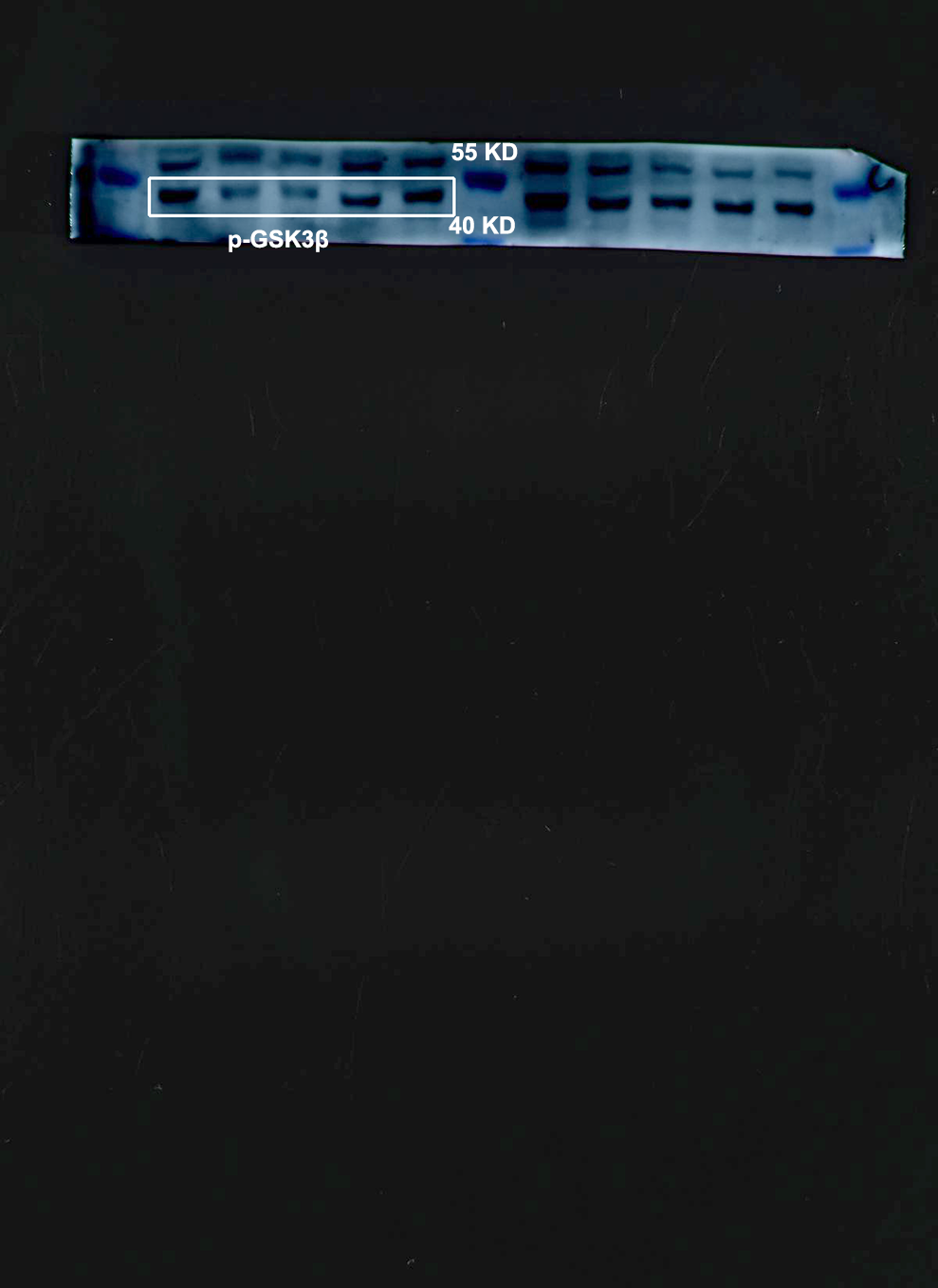

Supplement: Supplemental Information 4 [file peerj-12-17538-s004.zip › raw data-WB blots in Figure 7/gsk3a┬/p-gsk_145032_Ch_Chemi+Marker.tif]

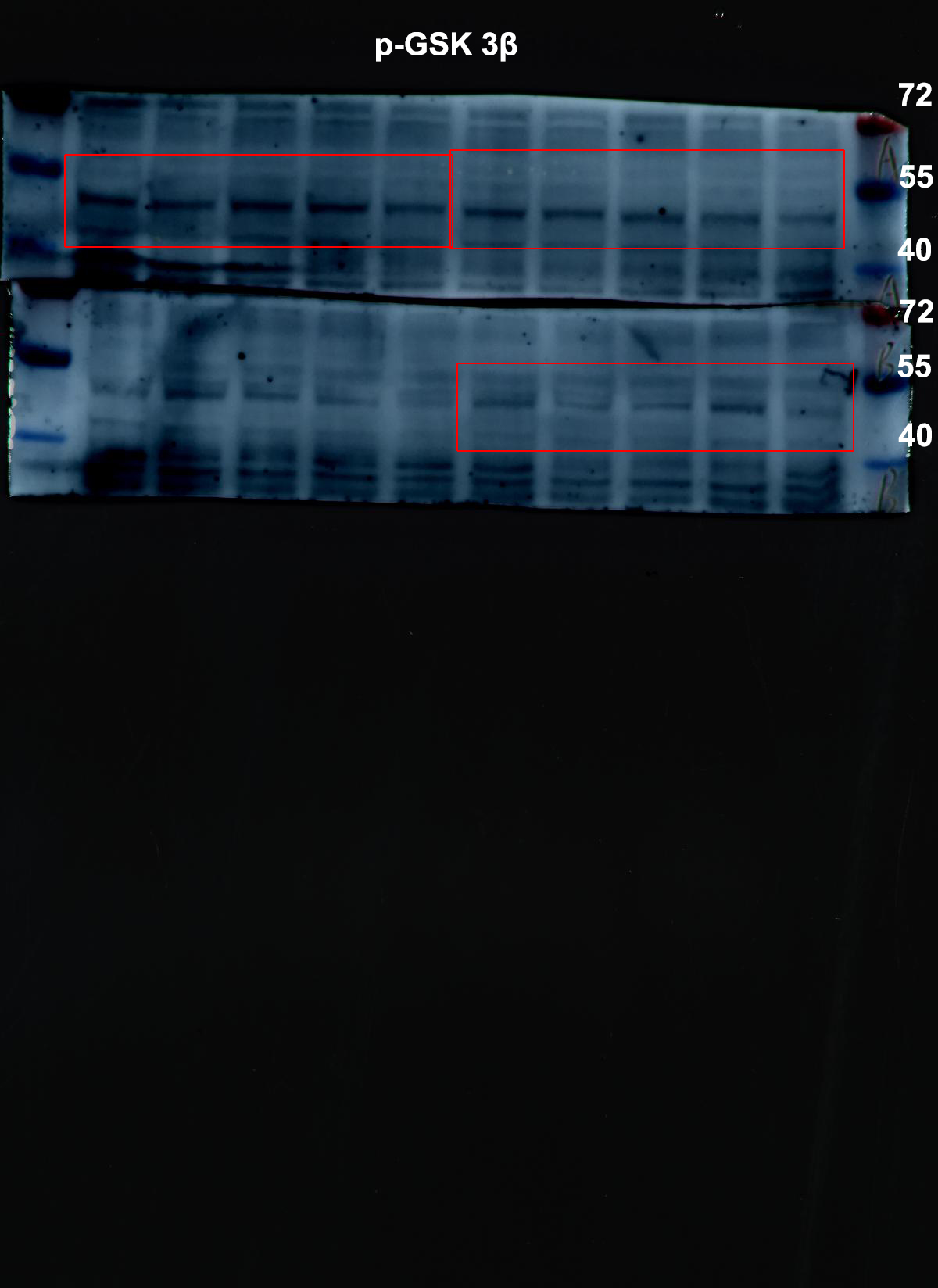

Supplement: Supplemental Information 4 [file peerj-12-17538-s004.zip › raw data-WB blots in Figure 7/gsk3a┬/p-gsk-3 _162528_Ch_Chemi+Marker.jpg]

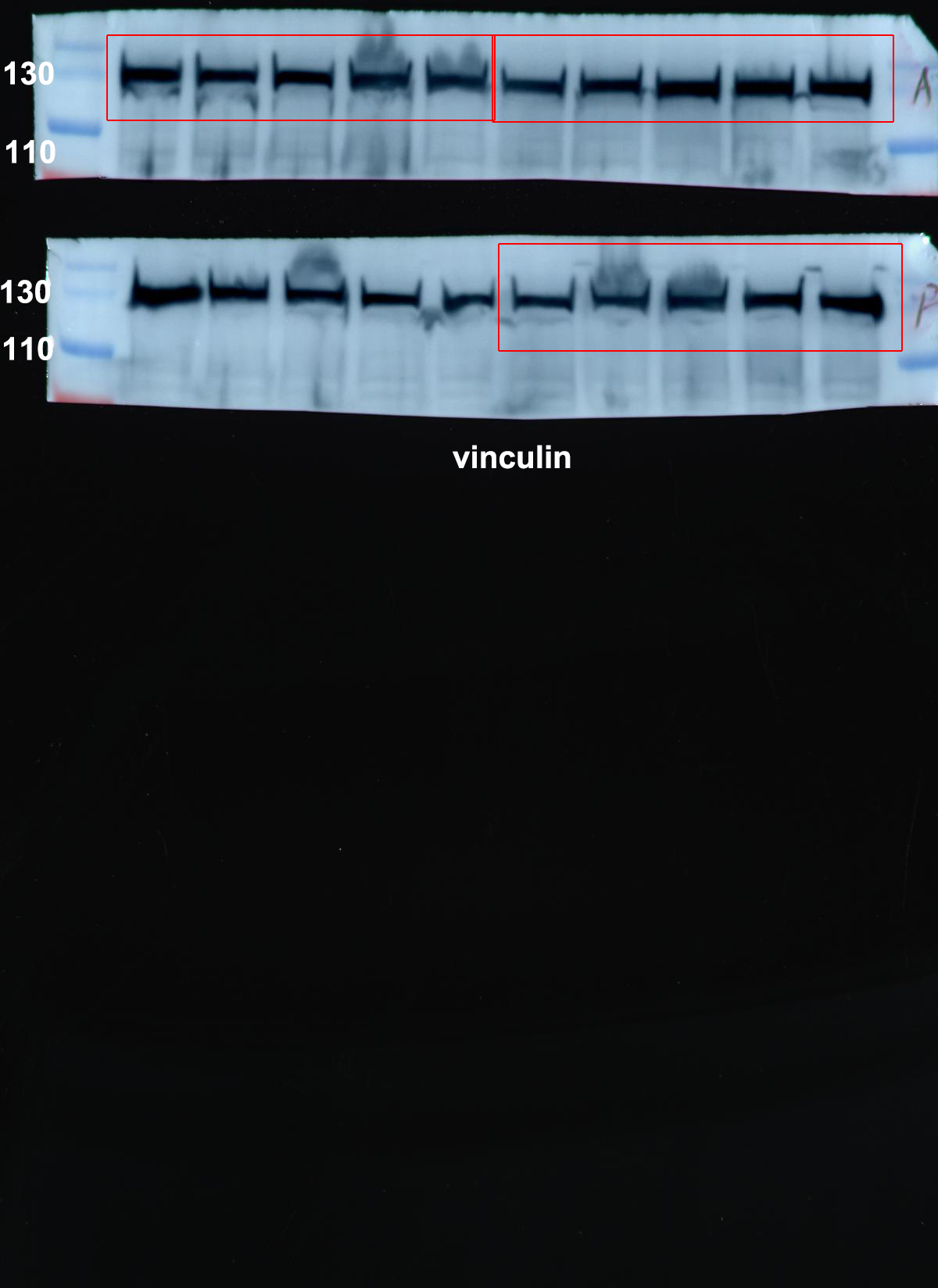

Supplement: Supplemental Information 4 [file peerj-12-17538-s004.zip › raw data-WB blots in Figure 7/gsk3a┬/vinculin _124918_Ch_Chemi+Marker.jpg]

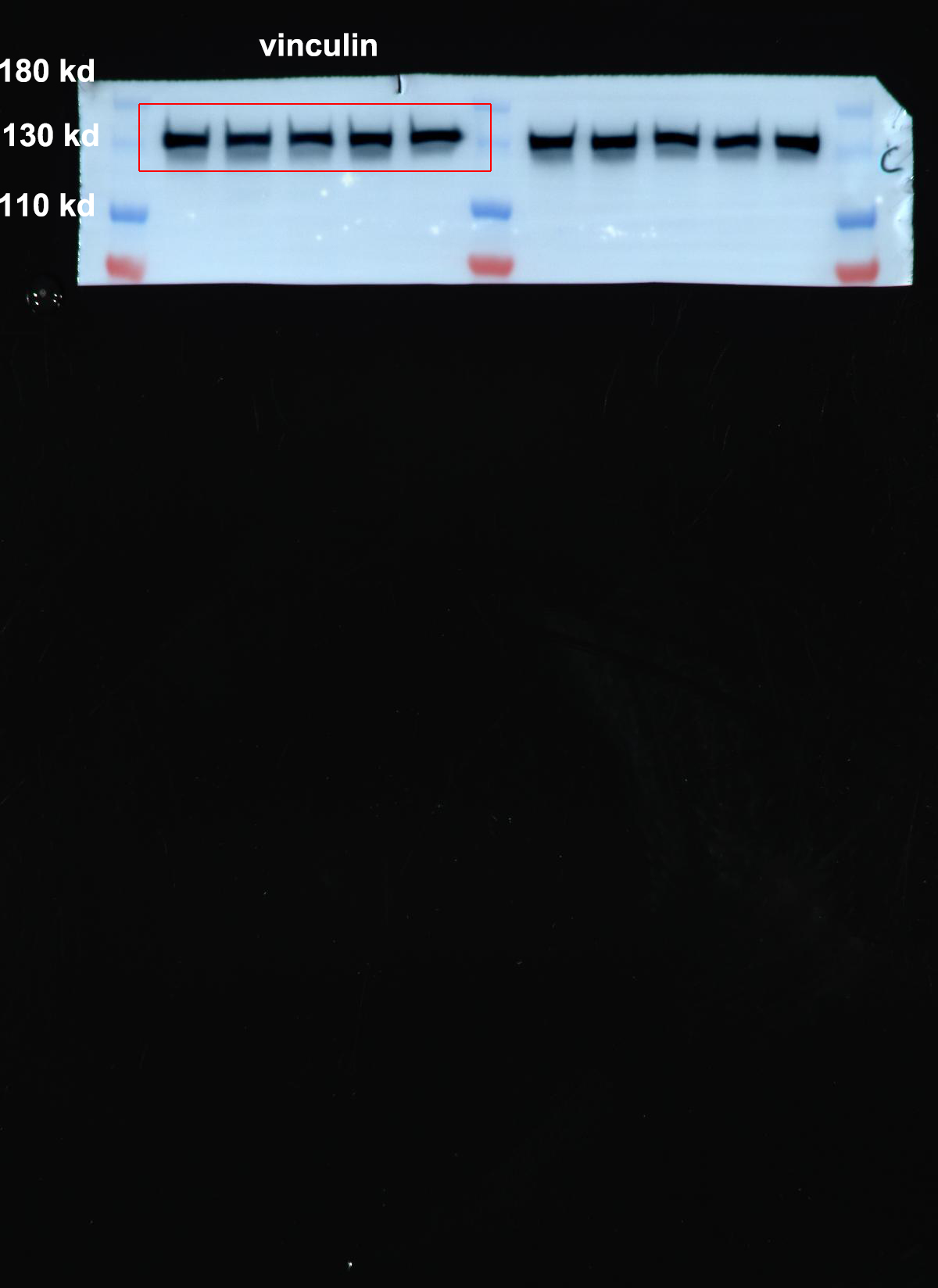

Supplement: Supplemental Information 4 [file peerj-12-17538-s004.zip › raw data-WB blots in Figure 7/gsk3a┬/vinculin-134806_Ch_Chemi+Marker.jpg]

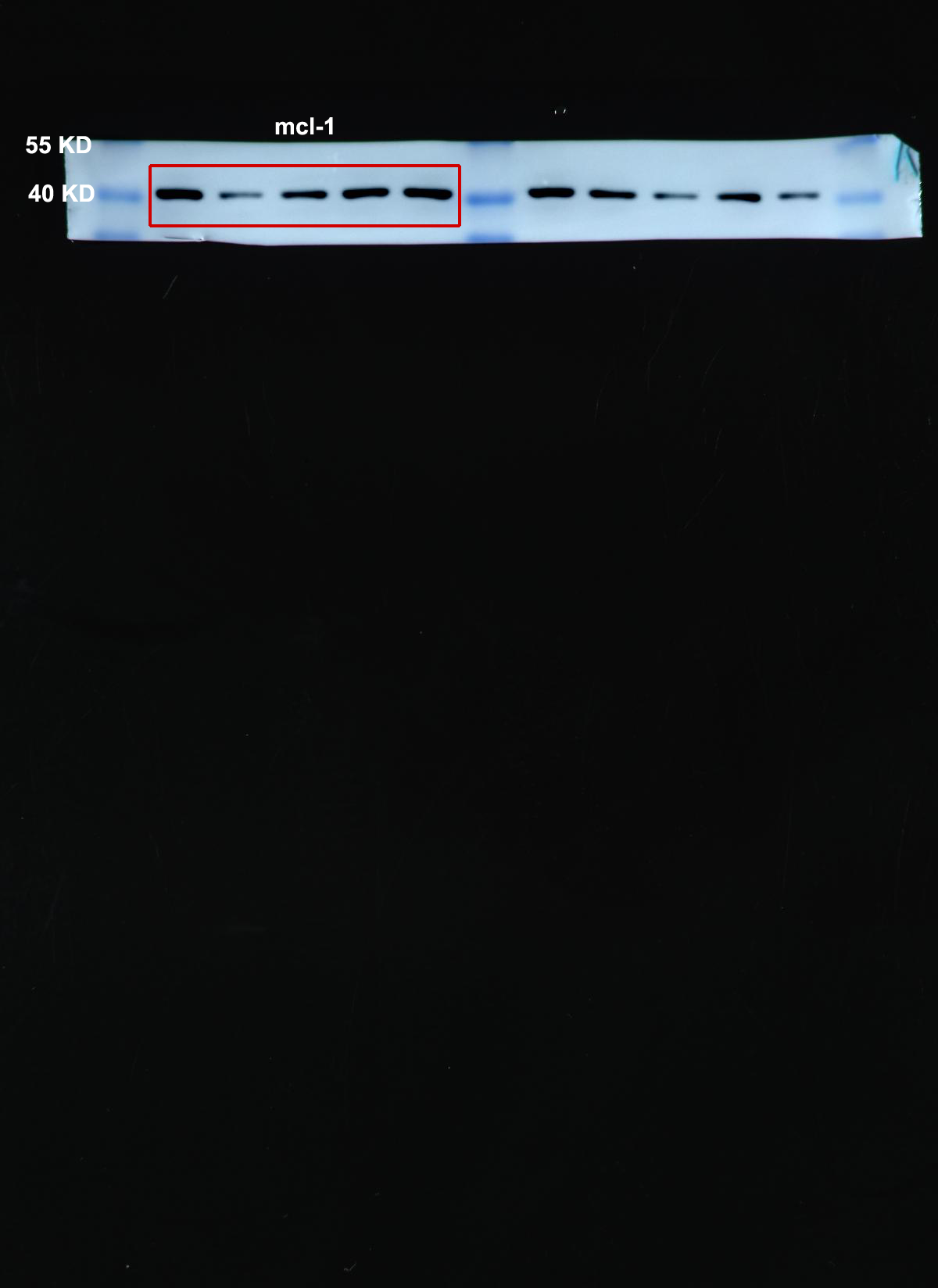

Supplement: Supplemental Information 4 [file peerj-12-17538-s004.zip › raw data-WB blots in Figure 7/mcl-1/mcl-1_140205_Ch_Chemi+Marker.tif]

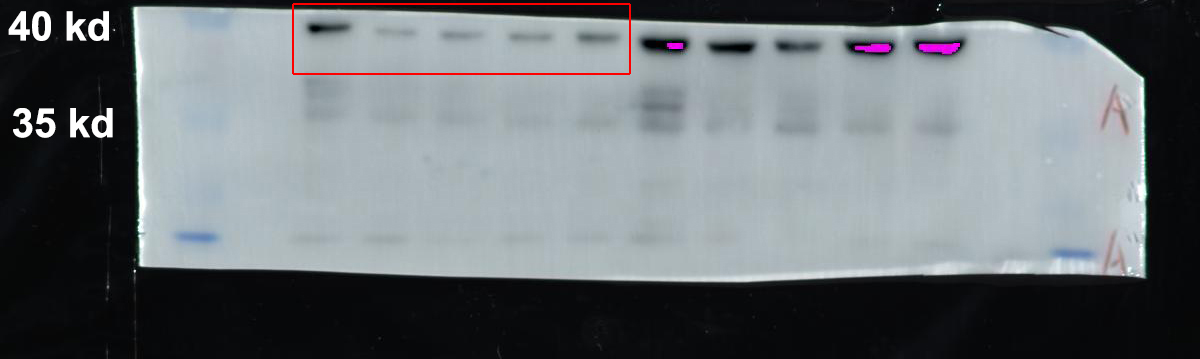

Supplement: Supplemental Information 4 [file peerj-12-17538-s004.zip › raw data-WB blots in Figure 7/mcl-1/mcl-1_144508-05_Ch_Chemi+Marker.jpg]

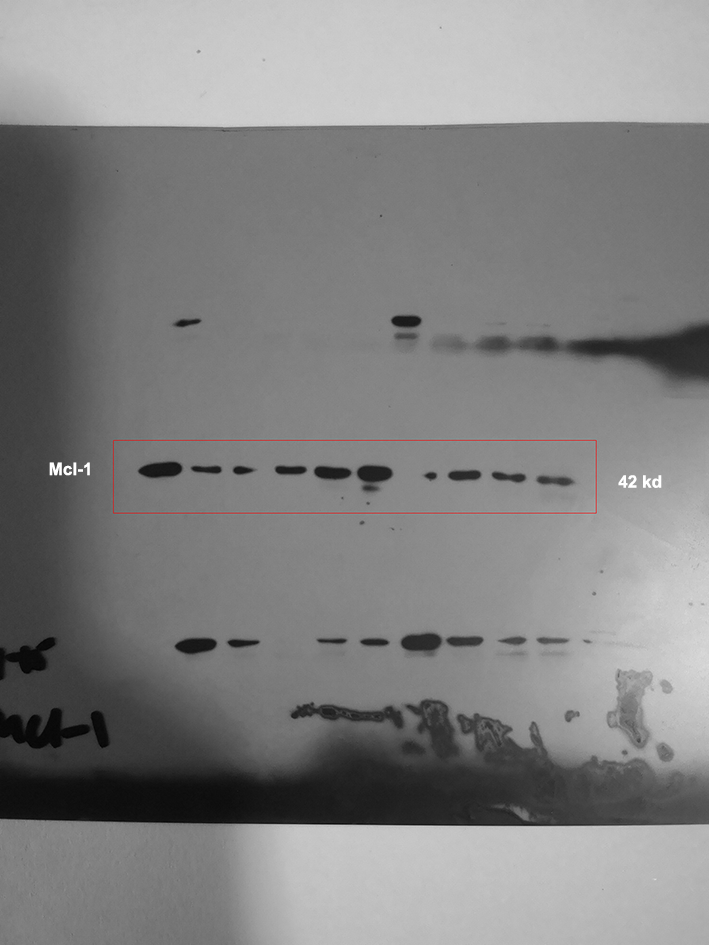

Supplement: Supplemental Information 4 [file peerj-12-17538-s004.zip › raw data-WB blots in Figure 7/mcl-1/mcl-1-20220115.tif]

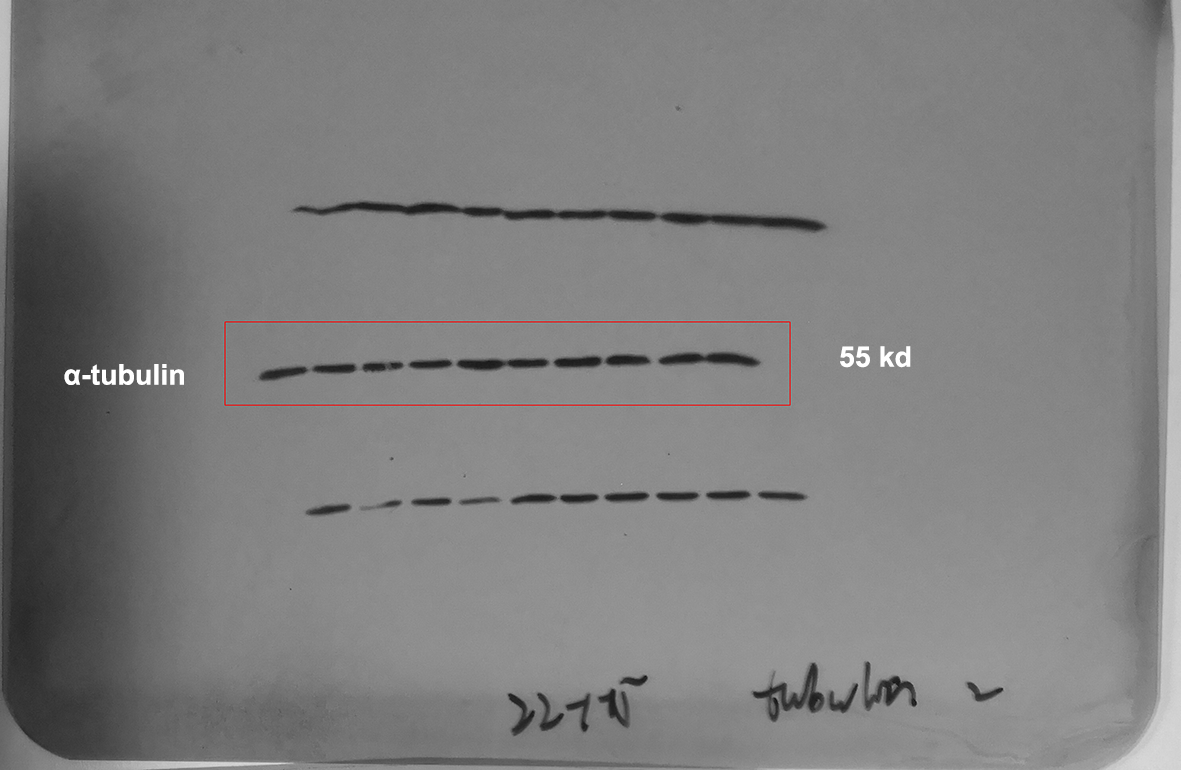

Supplement: Supplemental Information 4 [file peerj-12-17538-s004.zip › raw data-WB blots in Figure 7/mcl-1/tubulin-20220115.tif]

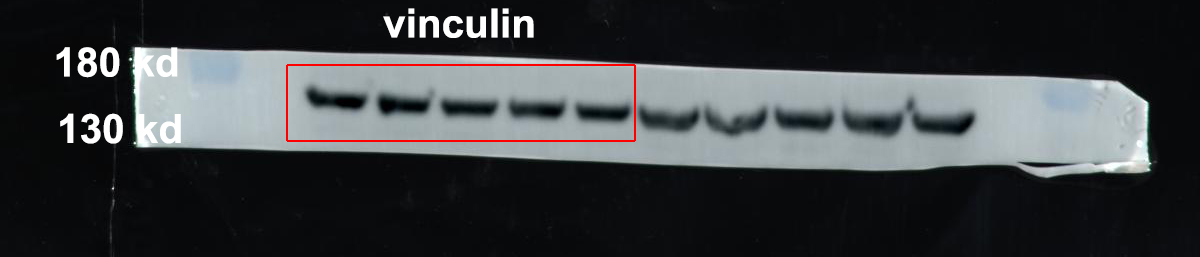

Supplement: Supplemental Information 4 [file peerj-12-17538-s004.zip › raw data-WB blots in Figure 7/mcl-1/vinculin_143202_Ch_Chemi+Marker.jpg]

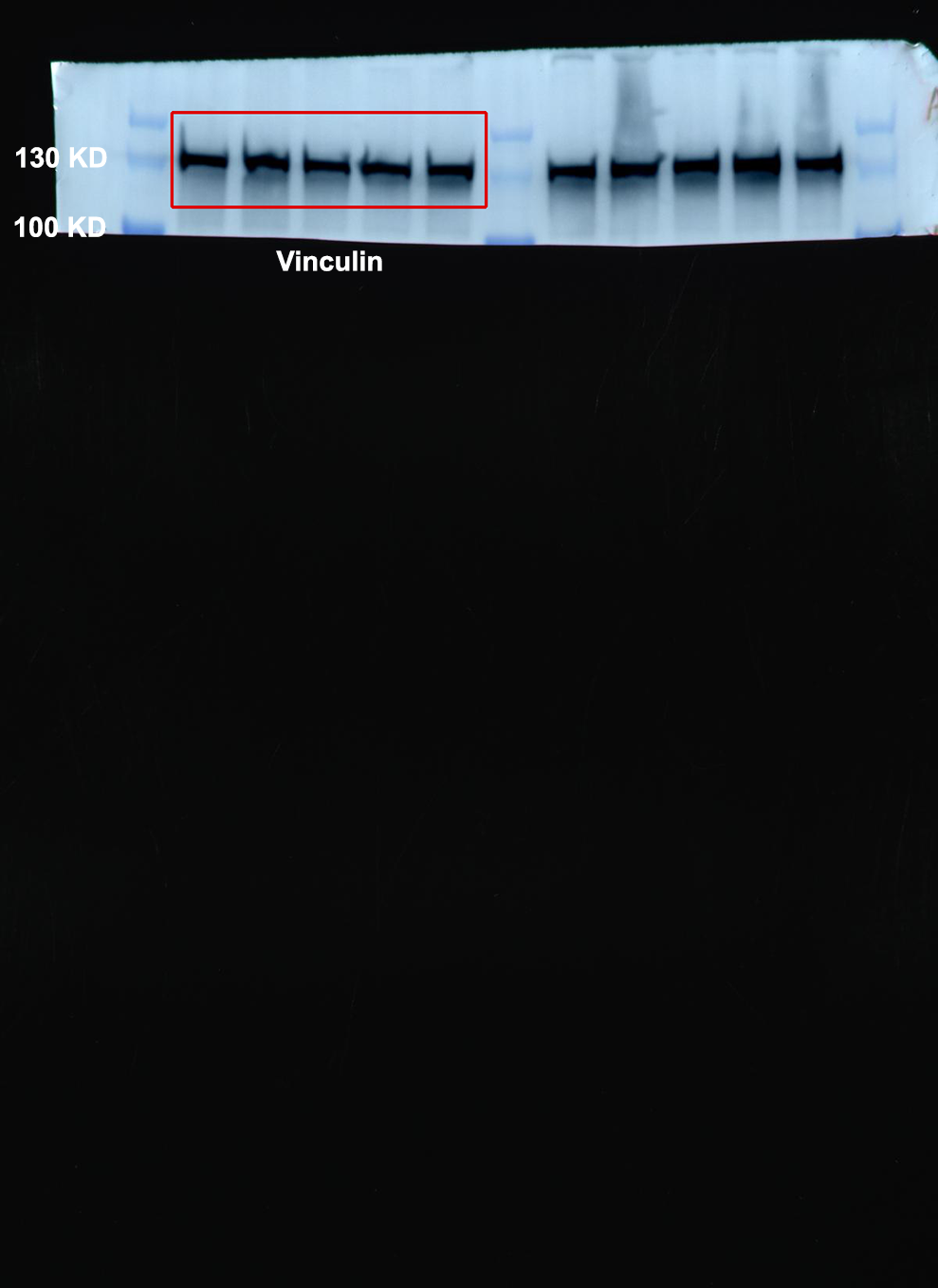

Supplement: Supplemental Information 4 [file peerj-12-17538-s004.zip › raw data-WB blots in Figure 7/mcl-1/vinculin_144940_Ch_Chemi+Marker.tif]

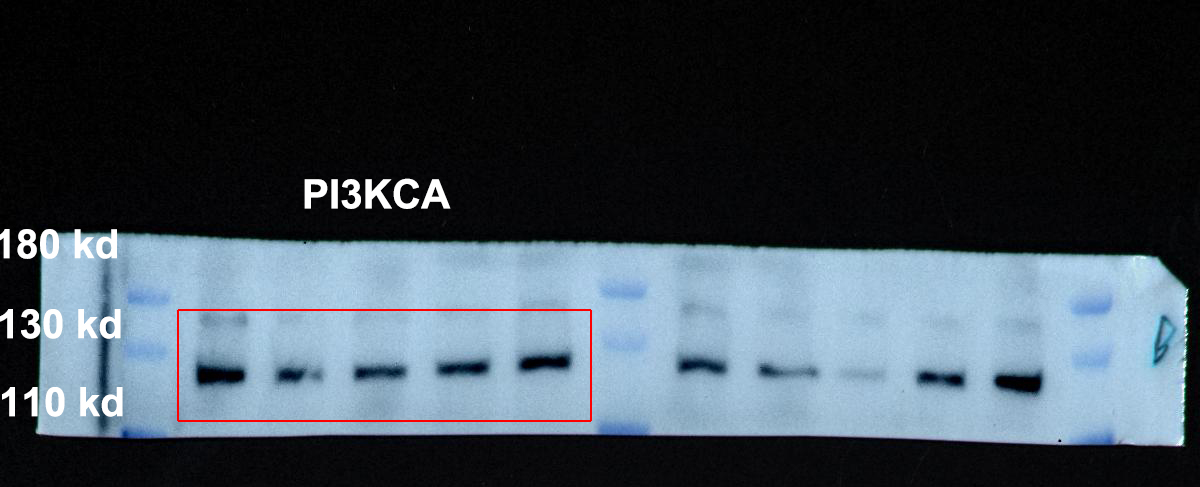

Supplement: Supplemental Information 4 [file peerj-12-17538-s004.zip › raw data-WB blots in Figure 7/pi3k/pi3kca _141545_Ch_Chemi+Marker.jpg]

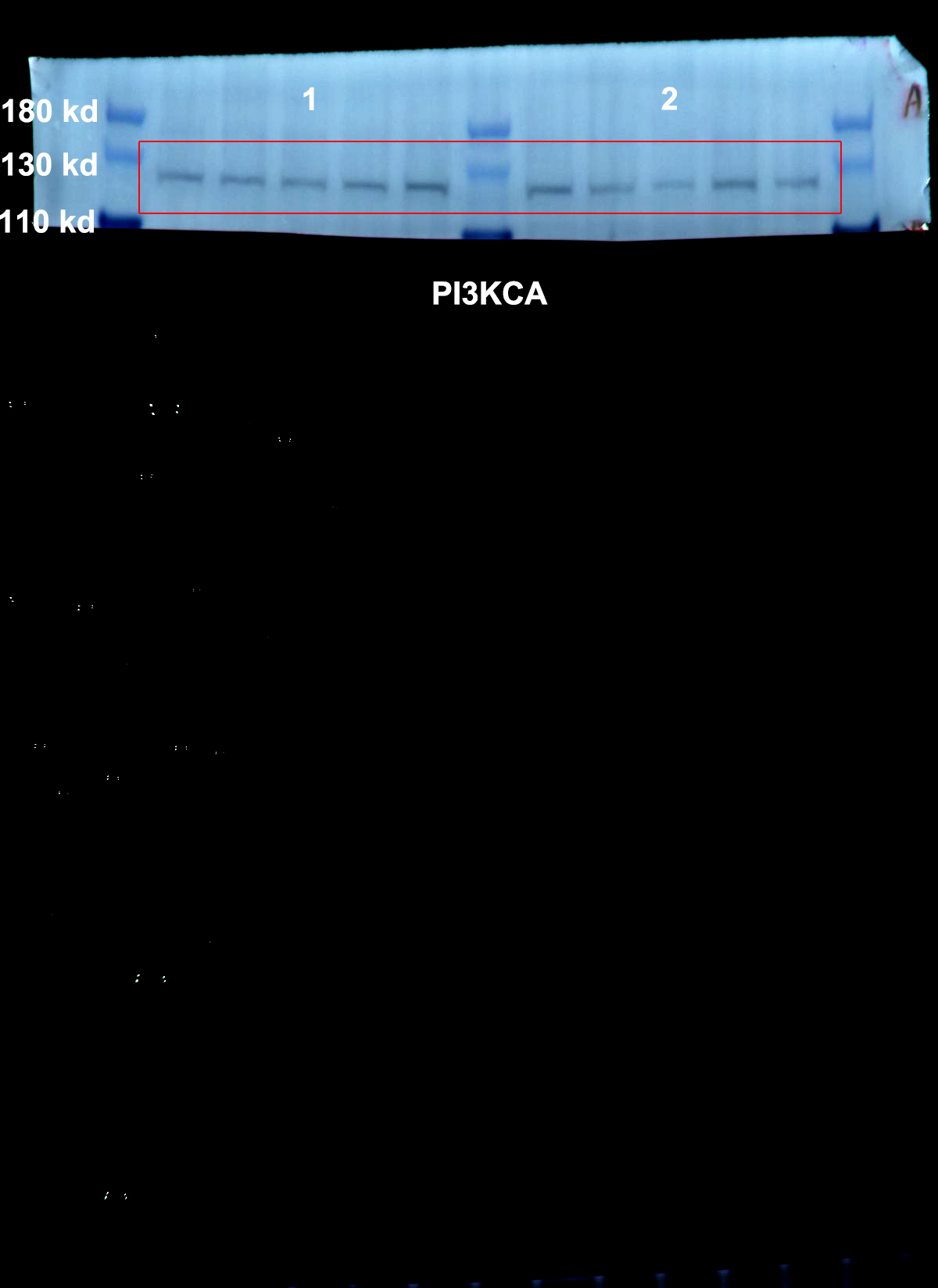

Supplement: Supplemental Information 4 [file peerj-12-17538-s004.zip › raw data-WB blots in Figure 7/pi3k/pi3kca _151736_Ch_Chemi+Marker.jpg]

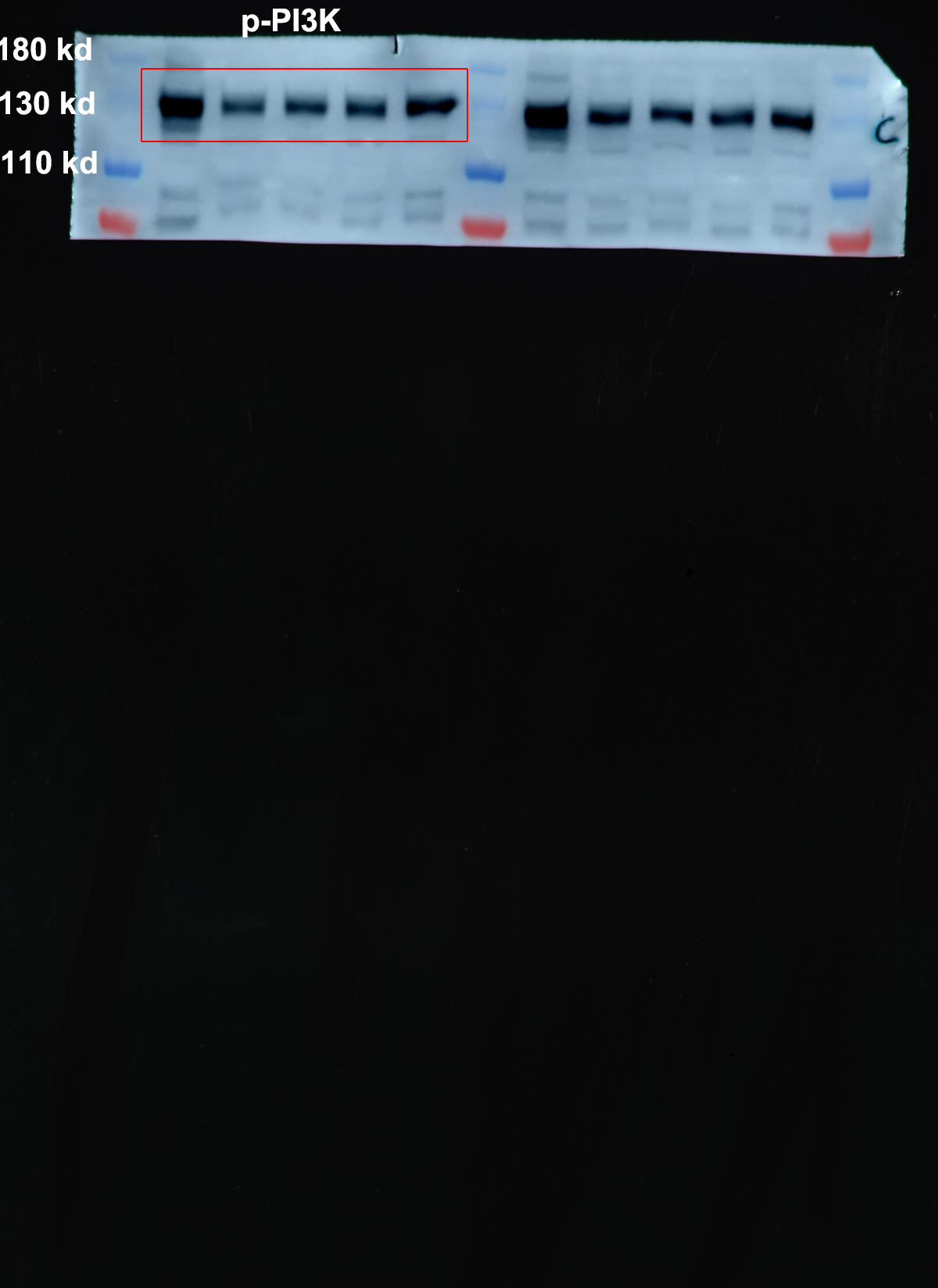

Supplement: Supplemental Information 4 [file peerj-12-17538-s004.zip › raw data-WB blots in Figure 7/pi3k/p-pi3k _142420_Ch_Chemi+Marker.jpg]

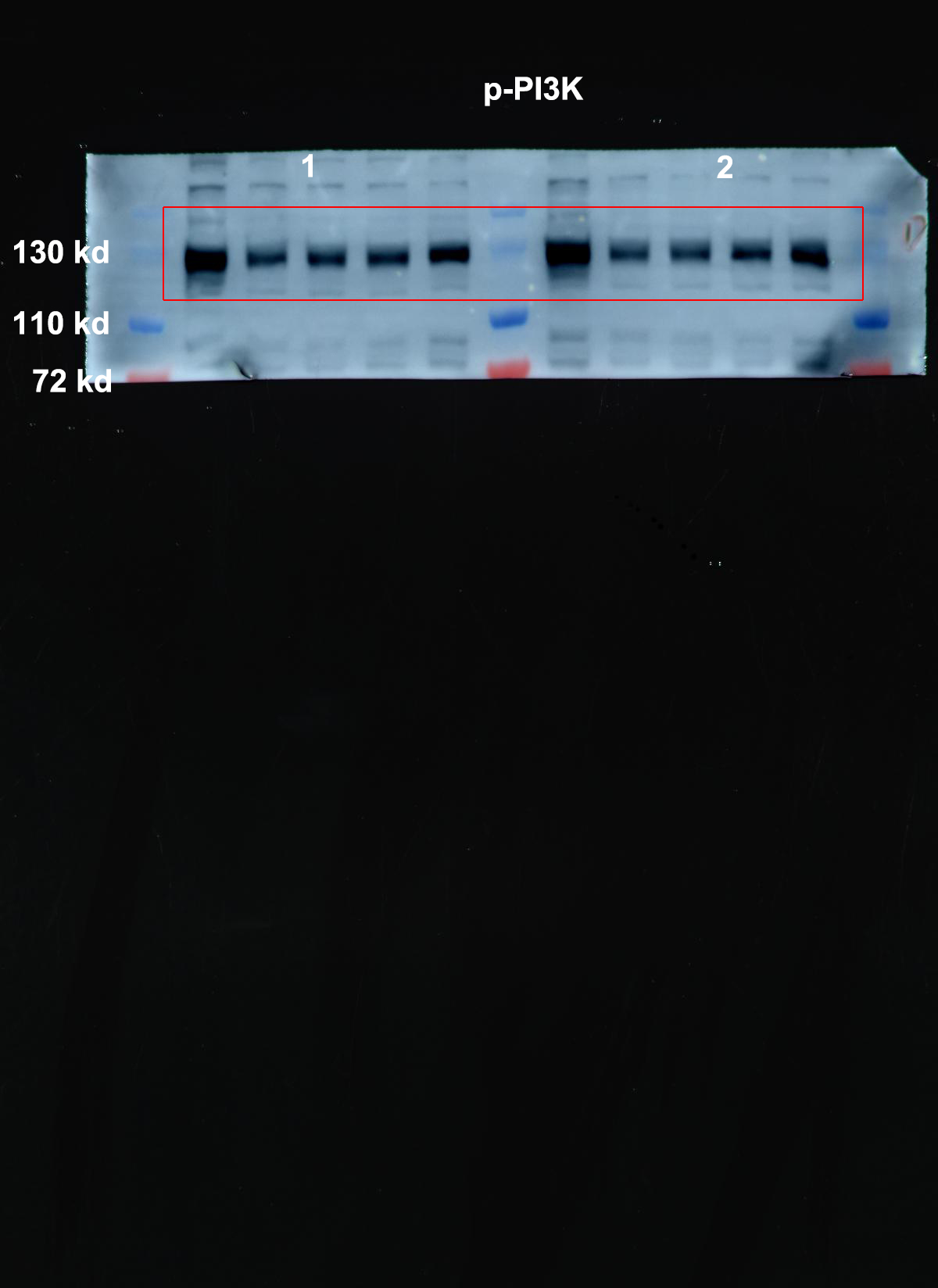

Supplement: Supplemental Information 4 [file peerj-12-17538-s004.zip › raw data-WB blots in Figure 7/pi3k/p-pi3k_143015_Ch_Chemi+Marker.jpg]

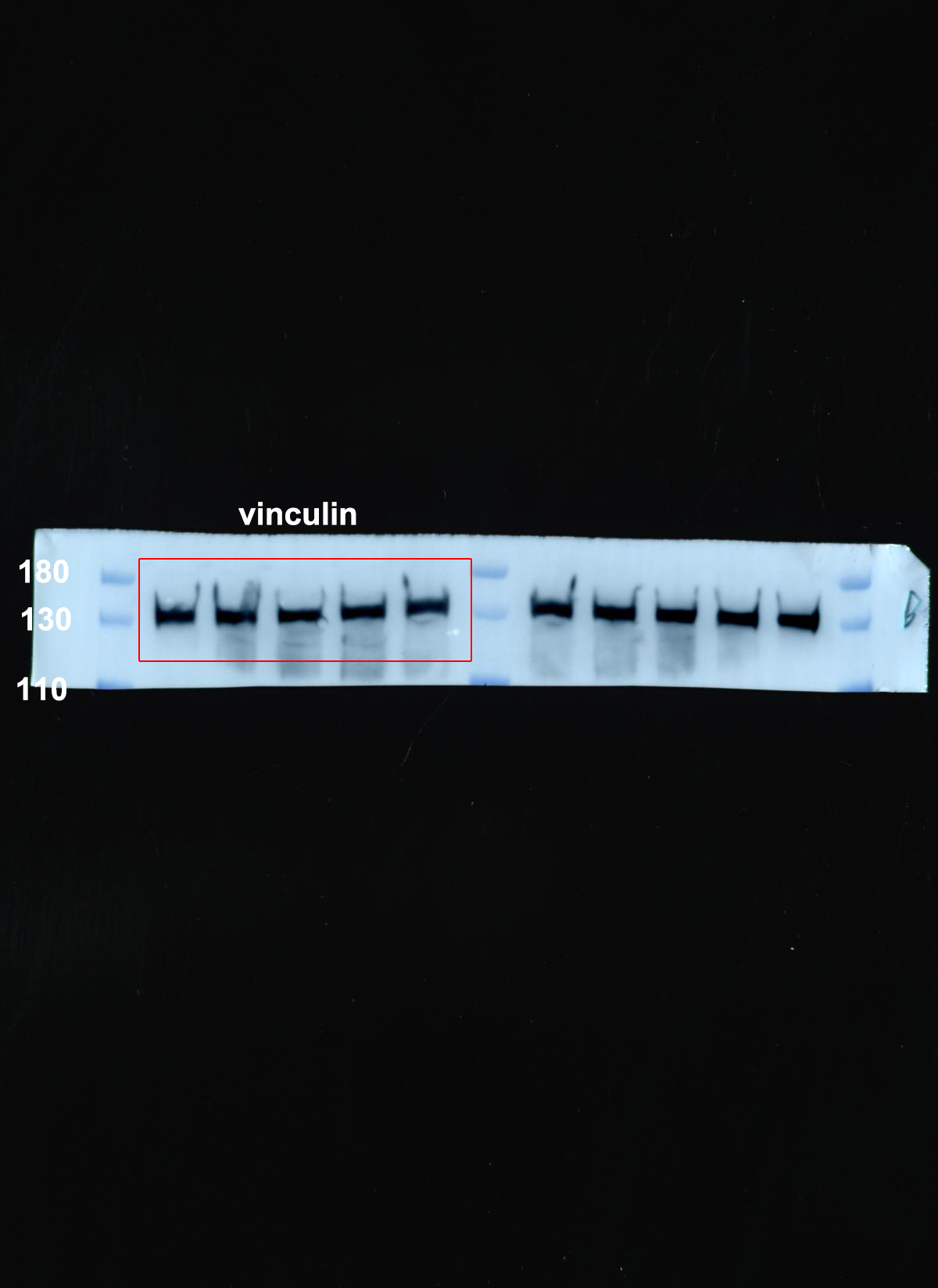

Supplement: Supplemental Information 4 [file peerj-12-17538-s004.zip › raw data-WB blots in Figure 7/pi3k/viculin_180312_Ch_Chemi+Marker.jpg]

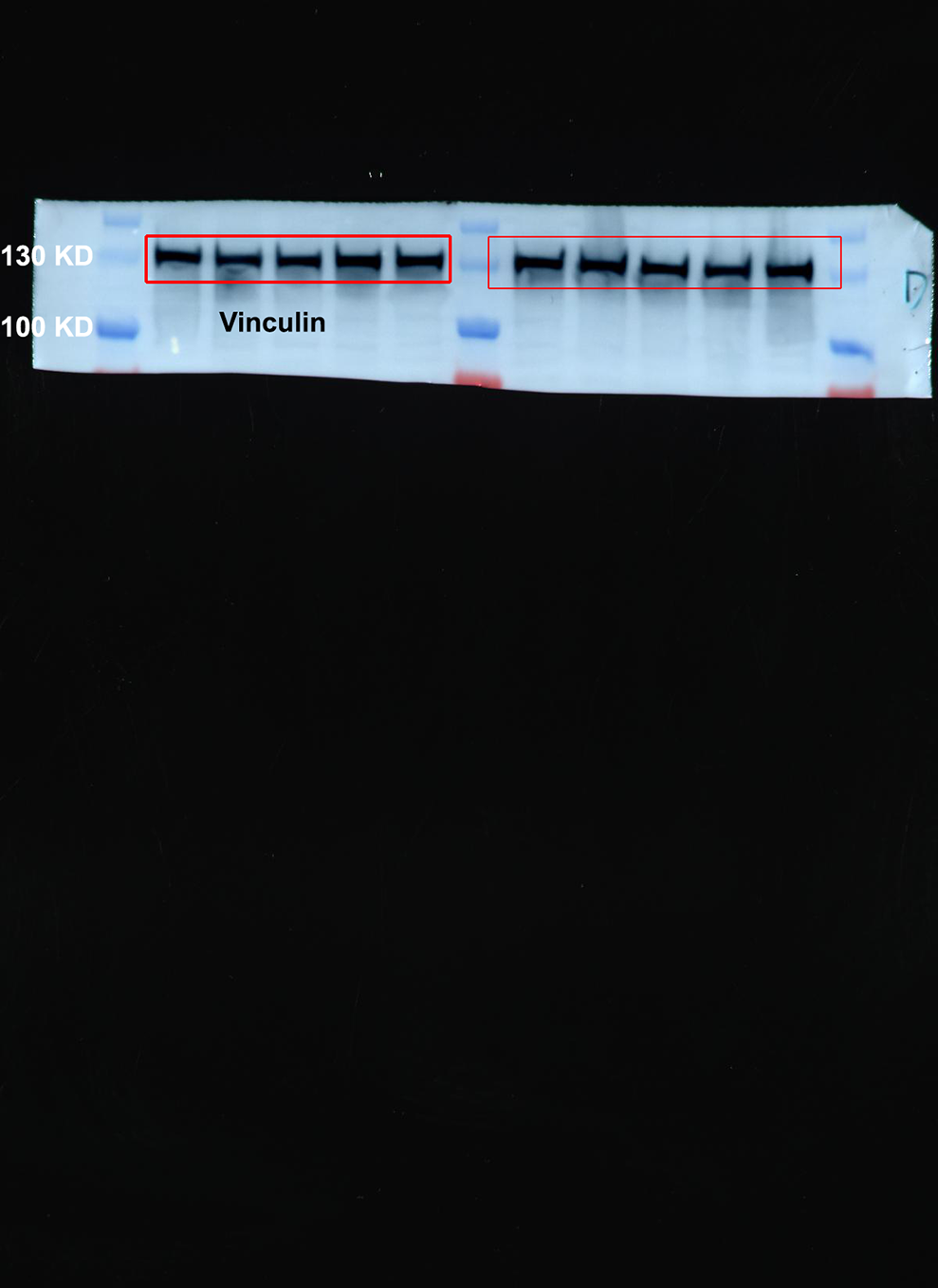

Supplement: Supplemental Information 4 [file peerj-12-17538-s004.zip › raw data-WB blots in Figure 7/pi3k/vinculin_145955_Ch_Chemi+Marker.tif]
